# Development and Implementation of a Pediatric Pulmonary–Focused Active Learning Curriculum

**DOI:** 10.15766/mep_2374-8265.11470

**Published:** 2024-12-06

**Authors:** Sanaz Vaziri, Fatima Neemuchwala, Janet Lim, Marilynn Chan

**Affiliations:** 1 Third-Year Fellow, Department of Pediatric Pulmonology, University of California, San Francisco, School of Medicine; 2 Assistant Professor, Department of Pediatric Pulmonology, University of California, San Francisco, School of Medicine; 3 Assistant Professor, Department of Pediatric Pulmonology, Loma Linda University School of Medicine; 4 Physician, Department of Pediatric Pulmonology, Kaiser Permanente

**Keywords:** Pediatric Pulmonary, Pediatrics, Chronic Cough, Asthma, Tracheostomy, Obstructive Sleep Apnea, Case-Based Learning

## Abstract

**Introduction:**

During the pediatric pulmonary rotation, trainees reported that while the clinical experience was robust, they desired more structured didactic sessions. Self-paced online modules can be an alternative but equally effective teaching tool. Multiple studies demonstrate the benefits of e-learning in medical education.

**Methods:**

Four interactive, case-based, e-learning modules on asthma, chronic cough, tracheostomy, and obstructive sleep apnea were created and piloted to pediatric residents and medical students in the pediatric pulmonology elective at the University of California, San Francisco and Oakland campus. Modules incorporated videos with experts, images, and multiple-choice questions to enhance learning. The modules were distributed via email on their first day of the rotation and were completed virtually by the trainees during their rotation. Progress was evaluated from December 2021 to July 2022 using pre- and postcourse questions assessing knowledge acquisition and decision-making skills.

**Results:**

A total of 22 participants took part in the elective and completed the modules to varying degrees. This group included six fourth-year medical students and three first-year, five second-year, and eight third-year pediatric residents. Data showed an improvement in posttest scores across all four modules, ranging from 9% to 21%, with a large improvement in the obstructive sleep apnea module and a small improvement in the chronic cough module. Trainees reported that the modules supplemented and increased their knowledge base.

**Discussion:**

Knowledge about managing common pulmonary conditions is critical for general pediatricians. Self-paced online modules are well accepted by trainees and are effective in improving knowledge and medical decision-making.

## Educational Objectives

By the end of this activity, learners will be able to:

1. Diagnose pulmonary conditions common in childhood such as chronic cough, asthma, and obstructive sleep apnea (OSA).
2. Manage the basics of chronic cough, asthma, OSA, and tracheostomy care.
3. Demonstrate an understanding of the proper techniques for metered dose inhalers, dry powder inhalers, and redihalers.

## Introduction

Respiratory conditions are one of the most common causes of acute primary care visits.^1^ General pediatricians often become the frontline providers for managing common respiratory symptoms prior to referral to a subspecialist. However, depending on practice location and the access to pediatric pulmonologists, many pediatricians become the medical home for these patients. Knowledge of common pulmonary diseases is critical for general pediatricians.

Although about 55% of pediatric residents go into general pediatrics,^2^ residency programs typically prioritize inpatient training, with residents spending less than 30% of their time in the outpatient setting.^3^ In addition, many residents receive little or no training in the management of chronic illness that often require interdisciplinary care, and these skills are difficult and expensive to obtain after residency.^4^ Standardization of learning outcomes and integrating formal knowledge and clinical experience can help fill in this training gap.

A needs assessment survey was completed by pediatric residents at our institution. Residents expressed a desire for structured education on common pulmonary topics. Based on the results of this survey, we developed and implemented four case-based, e-learning, interactive modules on common topics seen in pulmonology. E-learning offers an opportunity to overcome barriers to educational delivery, including accessibility and incorporation of novel instructional methods. E-learning has the added benefit of being asynchronous, which allows students to work at their own pace.^5^ Multiple studies have demonstrated it to be as effective as conventional didacticism.^6–8^ Although prior studies have implemented combined hands on training and visual presentations,^9,10^ this can often be challenging in a limited resource environment. In addition, with e-learning modules, trainees can access these during their rotation and even after completion of the rotation.

Our curriculum utilized a blended learning approach, which consisted of modules in conjunction with inpatient and outpatient experiences to foster knowledge and to train learners with the skills needed to provide quality care to their patients. These modules are primarily focused on outpatient care diagnosis and management. While e-learning modules have been created for asthma,^11^ no published reports exist on the effectiveness of e-learning modules in improving trainee knowledge of outpatient pediatric pulmonary conditions. Additionally, no similar case-based modules were found for learning about chronic cough, tracheostomy, and obstructive sleep apnea (OSA).

## Methods

### Needs Assessment Survey

A needs assessment survey was conducted in December 2020. The survey was distributed to pediatric residents at University of California, San Francisco (UCSF) and Oakland campus via the Qualtrics platform, with 66 responses collected. The residents were asked about what topics they would like to see covered in depth in the e-learning modules. The topics listed included asthma management, cystic fibrosis, chronic cough, basics of tracheostomies, OSA, pulmonary function testing, congenital lung malformations, and a fill-in response. The four topics of interest chosen were asthma, chronic cough, basics of tracheostomy management, and OSA. Based on these results, we developed and implemented four case-based, e-learning, interactive modules. These modules were created for medical students and pediatric residents participating in the pediatric pulmonary elective. Trainees were given time within their duty hours to complete the modules at their own pace throughout their 2- to 4-week elective. There were no other modules required during this rotation.

### Module Development

Modules were created in the Articulate Rise 360 (Articulate) e-learning platform. This software was used due to its user-friendly interface and wide range of interactive features including quizzes, simulations, and scenarios. A single user developed the modules, with each one requiring a 2-week period for creation, which included the time needed for revisions based on received feedback. Each case contained different interactive elements including videos, flashcards, and audio clips to enhance learning and maintain user engagement. Faculty recorded videos on Zoom Video Communications, which were then uploaded onto the Articulate platform. Continue button dividers were added to control the flow of the learner's navigation through the content, which required the learner to review all the content in one section before proceeding to the next.

### Access

The modules were published on the UCSF Learning Center website. A link to each module was created and added to the pediatric pulmonary curriculum, which was distributed via email in a Word document to the learners. Learners accessed the modules through the University of California learning website using their UCSF credentials. Upon publication of the modules on the website, information technology (IT) granted us access, allowing us to monitor completion status.

### Cases

The cases were created based on clinical scenarios that general pediatricians often face in an ambulatory setting. The goals of these modules were to help trainees develop practical skills in assessing and managing patients with different pulmonary conditions. The cases included:

• Alexa, a 7-year-old girl with a cough, diagnosed with asthma (Appendix A)
• Adam, a 13-month-old boy born prematurely with subsequent bronchopulmonary dysplasia, who is tracheostomy and ventilator dependent (Appendix B)
• Matteo, a 5-year-old boy with chronic cough, diagnosed with protracted bacterial bronchitis (Appendix C)
• Sean, a 6-year-old boy with snoring and gasping episodes, diagnosed with sleep apnea (Appendix D)

Each case began with a chief complaint and led the trainee through a conversation with the parent to obtain the history of present illness and the past medical, surgical, social, and family histories. An interactive physical exam, differential diagnosis, workup, and treatment were integrated into different subsections. The modules also incorporated videos of experts explaining key concepts (e.g., spirometry, proper technique for asthma inhalers).

### Feedback

Before implementing the modules, feedback was gathered from the pediatric pulmonology faculty to assess strengths and areas for improvement. Modules were shared with the faculty via email links. The faculty found the cases to be relevant and succinct. We also obtained feedback from the trainees who participated in the module, and changes were implemented following the monitoring period conducted from December 2021 to July 2022. Trainees also had an opportunity to provide qualitative feedback on the modules during one-on-one faculty review at the end of the elective.

### Assessment

Each module contained a pre- and posttest to assess knowledge and decision-making skills. The questions in the pre and posttest were identical and designed around the educational objectives of each module. Questions were designed by faculty and were made to resemble pediatric board-style questions centered around the diagnosis and management of chronic cough, asthma, tracheostomy care, and OSA. The pre- and posttest questions were on Qualtrics and embedded into the module. Each test consisted of five multiple-choice questions (Appendix E). The faculty involved with the module development received email notification of completion of pre- and posttests and could access their assessment score on Qualtrics. The average pre- and posttest scores of all learners were also analyzed according to trainee levels. In addition, informal feedback and formal evaluations were obtained from the residents and medical students after completion of the elective (Appendix F).

## Results

A total of 22 participants with various levels of medical education took part in the elective and completed the modules. Of the 22 participants, 27% (*n* = 6) were fourth-year medical students, 14% (*n* = 3) were first-year residents, 23% (*n* = 5) were second-year residents, and 36% (*n* = 8) were third-year residents. Results of the pre- and posttests were monitored between December 2021 to July 2022.

There were varied levels of engagement across each module, with 73% (*n* = 16) of trainees completing the OSA module, 100% (*n* = 22) completing the asthma module, and 82% (*n* = 18) of trainees completing the chronic cough module and tracheostomy module. Each module took around 25–45 minutes to complete. Overall, there was an improvement seen in average posttest scores across all four modules, which ranged from 9% to 21% (Figure), with the largest improvement seen in the OSA module and smallest improvement seen in the chronic cough module.

**Figure.**
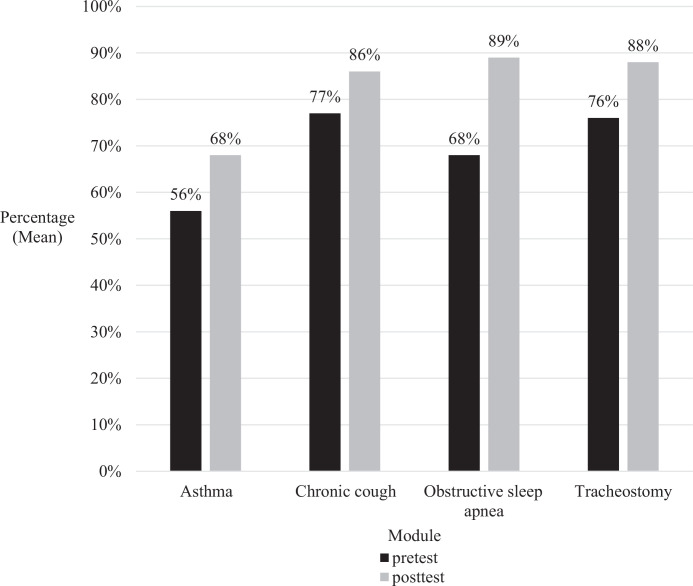
Mean pre- and posttest scores for each of the four modules. The chronic cough module exhibited the smallest improvement, with a 9% change from pre- to posttest scores. Conversely, the obstructive sleep apnea module showed the most significant improvement, with a notable 21% increase in scores.

When the data was grouped by training level, fourth-year medical students and first-year residents had lower pre- and posttest scores than second- and third-year residents. Second-year residents showed the greatest average improvement across all four modules when compared to the other training levels. Third-year residents had the highest scores in the pre- and posttest scores and had the smallest average improvement across all four modules (Table 1).

**Table 1.**
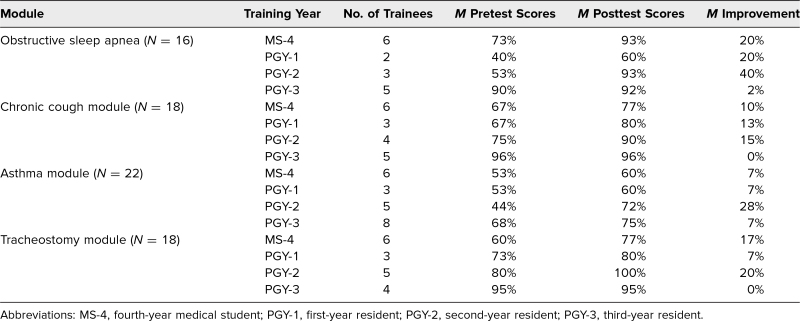
Mean Pre- and Posttest Scores Across Each of the Four Pediatric Pulmonary Modules by Training Level

### Feedback

The informal feedback and formal evaluations obtained from the residents and medical students after completion of the elective was obtained via email and in person. Trainees also left feedback in the postsurvey evaluation of the elective.

In general, trainees were satisfied with the cases. They found them relevant to the elective and enjoyed exposure to topics they commonly do not see. The cases improved their knowledge and confidence, specifically in history-taking, forming differentials, and medication techniques.

Trainees also gave feedback on specific components of the modules as well as indicated topics they would like added to the modules. Comments and feedback can be seen in Table 2.

**Table 2.**
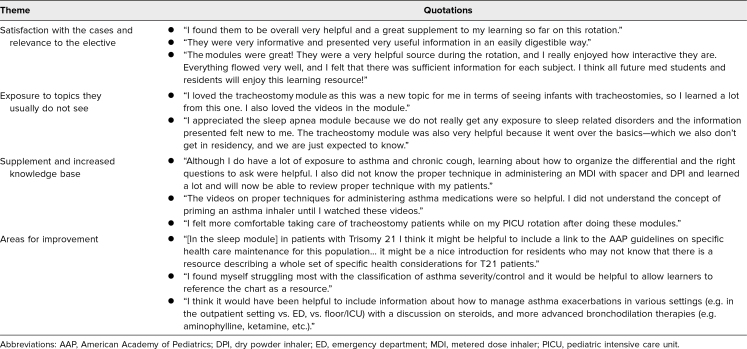
Feedback Received From Trainees

## Discussion

We have created and implemented four case-based learning modules in our pediatric pulmonary elective, which have been well received by trainees at our institution. Trainees have shown an improvement in mean pos-test scores across all modules, suggesting that online, case-based learning is a helpful adjunct in our pediatric pulmonary elective. As there is variability in the pulmonary diagnoses encountered by trainees during the elective, the modules provide a standardized level of exposure to common conditions they are likely to face during their training and in clinical practice.

Case-based learning is a productive way of presenting knowledge through clinical application and discussion. This type of learning has been shown to enhance clinical knowledge, improve clinical skills, and improve patient outcomes.^5–8^ Overall, there was an improvement in knowledge gained across all four modules. The highest increase was seen in the OSA module, which could be due to lack of exposure to sleep disorders during pediatric residency. When the results were grouped by training level, third-year residents had the smallest average improvement while second-year residents had the largest. This may suggest that while the modules are effective across all training levels, the greatest benefit was for medical students and first- and second-year trainees.

While there were several benefits noted with the modules, we did identify a few limitations. Although we did make the modules required, there was a variable level of engagement amongst trainees with the different cases, and some trainees did not complete all the modules. Additionally, while we assessed knowledge with pre- and posttest surveys, a disadvantage of this design is the possibility of participants benefiting from practice effect. Participants may remember or learn from the pretest questions, inflating posttest scores and making it difficult to determine the true effectiveness of the intervention. We also did not formally assess attitudes and perceptions towards the modules.

We plan to incorporate changes based on feedback received from learners and incorporate the modules into our institution's general pediatric curriculum to standardize pulmonary education for more learners. We also plan to create more pediatric pulmonary, case-based, interactive modules on topics of high demand, such as cystic fibrosis and basics of mechanical ventilation.

Respiratory conditions are the most common reasons for pediatrician visits. Depending on practice location, access to subspecialty care may be limited and frontline practitioners may have to independently manage children with these respiratory conditions. Knowledge and familiarity with these presentations is critical for general pediatricians. Case-based, interactive modules are an effective way to improve knowledge amongst trainees.

## Appendices


Asthma Module folderTracheostomy Module folderChronic Cough Module folderObstructive Sleep Apnea Module folderPosttest Questions.docxFeedback.docx
*All appendices are peer reviewed as integral parts of the Original Publication.*
