# Supplementary material for: Development and Implementation of a Pediatric Pulmonary–Focused Active Learning Curriculum
Source: MedEdPORTAL. 2024 Dec 6;20:11470. doi: 10.15766/mep_2374-8265.11470 (PMC11621239; doi:10.15766/mep_2374-8265.11470)
Supplement: Supplementary file 1 — Asthma Module folderTracheostomy Module folderChronic Cough Module folderObstructive Sleep Apnea Module folderPosttest Questions.docxFeedback.docx [file mep_2374-8265.11470-s001.zip › _Educational Summary Report_11470.pdf]

MedEdPORTAL® The AAMC Journal of Teaching and Learning Resources

Original Publication Open Access

Development and Implementation of a Pediatric Pulmonary–Focused Active Learning Curriculum

Sanaz Vaziri, MD*, Fatima Neemuchwala, MD, Janet Lim, MD, Marilynn Chan, MD

*Corresponding author: sanaz.vaziri@ucsf.edu

Abstract

Introduction: During the pediatric pulmonary rotation, trainees reported that while the clinical experience was robust, they desired more structured didactic sessions. Self-paced online modules can be an alternative but equally effective teaching tool. Multiple studies demonstrate the benefits of e-learning in medical education. **Methods:** Four interactive, case-based, e-learning modules on asthma, chronic cough, tracheostomy, and obstructive sleep apnea were created and piloted to pediatric residents and medical students in the pediatric pulmonology elective at the University of California, San Francisco and Oakland campus. Modules incorporated videos with experts, images, and multiple-choice questions to enhance learning. The modules were distributed via email on their first day of the rotation and were completed virtually by the trainees during their rotation. Progress was evaluated from December 2021 to July 2022 using pre- and postcourse questions assessing knowledge acquisition and decision-making skills. **Results:** A total of 22 participants took part in the elective and completed the modules to varying degrees. This group included six fourth-year medical students and three first-year, five second-year, and eight third-year pediatric residents. Data showed an improvement in posttest scores across all four modules, ranging from 9% to 21%, with a large improvement in the obstructive sleep apnea module and a small improvement in the chronic cough module. Trainees reported that the modules supplemented and increased their knowledge base. **Discussion:** Knowledge about managing common pulmonary conditions is critical for general pediatricians. Self-paced online modules are well accepted by trainees and are effective in improving knowledge and medical decision-making.

Keywords
Pediatric Pulmonary, Pediatrics, Chronic Cough, Asthma, Tracheostomy, Obstructive Sleep Apnea, Case-Based Learning

Educational Objectives

By the end of this activity, learners will be able to:

1. Diagnose pulmonary conditions common in childhood such as chronic cough, asthma, and obstructive sleep apnea (OSA).
2. Manage the basics of chronic cough, asthma, OSA, and tracheostomy care.
3. Demonstrate an understanding of the proper techniques for metered dose inhalers, dry powder inhalers, and redihalers.

Citation:
Vaziri S, Neemuchwala F, Lim J, Chan M. Development and implementation of a pediatric pulmonary–focused active learning curriculum. *MedEdPORTAL*. 2024;20:11470.
https://doi.org/10.15766/mep_2374-8265.11470

Introduction

Respiratory conditions are one of the most common causes of acute primary care visits.[1] General pediatricians often become the frontline providers for managing common respiratory symptoms prior to referral to a subspecialist. However, depending on practice location and the access to pediatric pulmonologists, many pediatricians become the medical home for these patients. Knowledge of common pulmonary diseases is critical for general pediatricians.

Although about 55% of pediatric residents go into general pediatrics,[2] residency programs typically prioritize inpatient training, with residents spending less than 30% of their time in the outpatient setting.[3] In addition, many residents receive little or no training in the management of chronic illness that often require interdisciplinary care, and these skills are difficult and expensive to obtain after residency.[4] Standardization of learning outcomes and integrating formal knowledge and clinical experience can help fill in this training gap.

Copyright © 2024 Vaziri et al. This is an open-access publication distributed under the terms of the Creative Commons Attribution license.

A needs assessment survey was completed by pediatric residents at our institution. Residents expressed a desire for structured education on common pulmonary topics. Based on the results of this survey, we developed and implemented four case-based, e-learning, interactive modules on common topics seen in pulmonology. E-learning offers an opportunity to overcome barriers to educational delivery, including accessibility and incorporation of novel instructional methods. E-learning has the added benefit of being asynchronous, which allows students to work at their own pace.[5] Multiple studies have demonstrated it to be as effective as conventional didacticism.[6-8] Although prior studies have implemented combined hands on training and visual presentations,[9,10] this can often be challenging in a limited resource environment. In addition, with e-learning modules, trainees can access these during their rotation and even after completion of the rotation.

Our curriculum utilized a blended learning approach, which consisted of modules in conjunction with inpatient and outpatient experiences to foster knowledge and to train learners with the skills needed to provide quality care to their patients. These modules are primarily focused on outpatient care diagnosis and management. While e-learning modules have been created for asthma,[11] no published reports exist on the effectiveness of e-learning modules in improving trainee knowledge of outpatient pediatric pulmonary conditions. Additionally, no similar case-based modules were found for learning about chronic cough, tracheostomy, and obstructive sleep apnea (OSA).

Methods

Needs Assessment Survey

A needs assessment survey was conducted in December 2020. The survey was distributed to pediatric residents at University of California, San Francisco (UCSF) and Oakland campus via the Qualtrics platform, with 66 responses collected. The residents were asked about what topics they would like to see covered in depth in the e-learning modules. The topics listed included asthma management, cystic fibrosis, chronic cough, basics of tracheostomies, OSA, pulmonary function testing, congenital lung malformations, and a fill-in response. The four topics of interest chosen were asthma, chronic cough, basics of tracheostomy management, and OSA. Based on these results, we developed and implemented four case-based, e-learning, interactive modules. These modules were created for medical students and pediatric residents participating in the pediatric pulmonary elective. Trainees were given time within their duty hours to complete the modules at their own pace throughout their 2- to 4-week elective. There were no other modules required during this rotation.

Module Development

Modules were created in the Articulate Rise 360 (Articulate) e-learning platform. This software was used due to its user-friendly interface and wide range of interactive features including quizzes, simulations, and scenarios. A single user developed the modules, with each one requiring a 2-week period for creation, which included the time needed for revisions based on received feedback. Each case contained different interactive elements including videos, flashcards, and audio clips to enhance learning and maintain user engagement. Faculty recorded videos on Zoom Video Communications, which were then uploaded onto the Articulate platform. Continue button dividers were added to control the flow of the learner's navigation through the content, which required the learner to review all the content in one section before proceeding to the next.

Access

The modules were published on the UCSF Learning Center website. A link to each module was created and added to the pediatric pulmonary curriculum, which was distributed via email in a Word document to the learners. Learners accessed the modules through the University of California learning website using their UCSF credentials. Upon publication of the modules on the website, information technology (IT) granted us access, allowing us to monitor completion status.

Cases

The cases were created based on clinical scenarios that general pediatricians often face in an ambulatory setting. The goals of these modules were to help trainees develop practical skills in assessing and managing patients with different pulmonary conditions. The cases included:

- Alexa, a 7-year-old girl with a cough, diagnosed with asthma (Appendix A)
- Adam, a 13-month-old boy born prematurely with subsequent bronchopulmonary dysplasia, who is tracheostomy and ventilator dependent (Appendix B)
- Matteo, a 5-year-old boy with chronic cough, diagnosed with protracted bacterial bronchitis (Appendix C)
- Sean, a 6-year-old boy with snoring and gasping episodes, diagnosed with sleep apnea (Appendix D)

Each case began with a chief complaint and led the trainee through a conversation with the parent to obtain the history of present illness and the past medical, surgical, social, and family histories. An interactive physical exam, differential diagnosis,

Copyright © 2024 Vaziri et al. This is an open-access publication distributed under the terms of the Creative Commons Attribution license.

workup, and treatment were integrated into different subsections. The modules also incorporated videos of experts explaining key concepts (e.g., spirometry, proper technique for asthma inhalers).

Feedback

Before implementing the modules, feedback was gathered from the pediatric pulmonology faculty to assess strengths and areas for improvement. Modules were shared with the faculty via email links. The faculty found the cases to be relevant and succinct. We also obtained feedback from the trainees who participated in the module, and changes were implemented following the monitoring period conducted from December 2021 to July 2022. Trainees also had an opportunity to provide qualitative feedback on the modules during one-on-one faculty review at the end of the elective.

Assessment

Each module contained a pre- and posttest to assess knowledge and decision-making skills. The questions in the pre and posttest were identical and designed around the educational objectives of each module. Questions were designed by faculty and were made to resemble pediatric board-style questions centered around the diagnosis and management of chronic cough, asthma, tracheostomy care, and OSA. The pre- and posttest questions were on Qualtrics and embedded into the module. Each test consisted of five multiple-choice questions (Appendix E). The faculty involved with the module development received email notification of completion of pre- and posttests and could access their assessment score on Qualtrics. The average pre- and posttest scores of all learners were also analyzed according to trainee levels. In addition, informal feedback and formal evaluations were obtained from the residents and medical students after completion of the elective (Appendix F).

Results

A total of 22 participants with various levels of medical education took part in the elective and completed the modules. Of the 22 participants, 27% ($n = 6$) were fourth-year medical students, 14% ($n = 3$) were first-year residents, 23% ($n = 5$) were second-year residents, and 36% ($n = 8$) were third-year residents. Results of the pre- and posttests were monitored between December 2021 to July 2022.

There were varied levels of engagement across each module, with 73% ($n = 16$) of trainees completing the OSA module, 100% ($n = 22$) completing the asthma module, and 82% ($n = 18$) of trainees completing the chronic cough module and tracheostomy module. Each module took around 25-45 minutes to complete. Overall, there was an improvement seen in average posttest scores across all four modules, which ranged from 9% to 21% (Figure), with the largest improvement seen in the OSA module and smallest improvement seen in the chronic cough module.

When the data was grouped by training level, fourth-year medical students and first-year residents had lower pre- and posttest scores than second- and third-year residents. Second-year residents showed the greatest average improvement across all four modules when compared to the other training levels. Third-year residents had the highest scores in the pre- and posttest scores and had the smallest average improvement across all four modules (Table 1).

Feedback

The informal feedback and formal evaluations obtained from the residents and medical students after completion of the elective was obtained via email and in person. Trainees also left feedback in the postsurvey evaluation of the elective.

In general, trainees were satisfied with the cases. They found them relevant to the elective and enjoyed exposure to topics they commonly do not see. The cases improved their knowledge and confidence, specifically in history-taking, forming differentials, and medication techniques.

Trainees also gave feedback on specific components of the modules as well as indicated topics they would like added to the modules. Comments and feedback can be seen in Table 2.

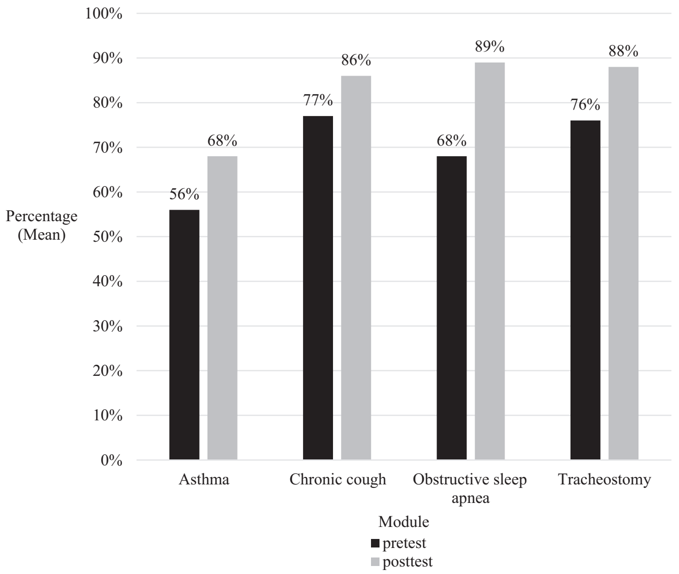

Figure. Mean pre- and posttest scores for each of the four modules. The chronic cough module exhibited the smallest improvement, with a 9% change from pre- to posttest scores. Conversely, the obstructive sleep apnea module showed the most significant improvement, with a notable 21% increase in scores.

Copyright © 2024 Vaziri et al. This is an open-access publication distributed under the terms of the Creative Commons Attribution license.

Table 1. Mean Pre- and Posttest Scores Across Each of the Four Pediatric Pulmonary Modules by Training Level

Module	Training Year	No. of Trainees	*M* Pretest Scores	*M* Posttest Scores	*M* Improvement
Obstructive sleep apnea (*N* = 16)	MS-4	6	73%	93%	20%
	PGY-1	2	40%	60%	20%
	PGY-2	3	53%	93%	40%
	PGY-3	5	90%	92%	2%
Chronic cough module (*N* = 18)	MS-4	6	67%	77%	10%
	PGY-1	3	67%	80%	13%
	PGY-2	4	75%	90%	15%
	PGY-3	5	96%	96%	0%
Asthma module (*N* = 22)	MS-4	6	53%	60%	7%
	PGY-1	3	53%	60%	7%
	PGY-2	5	44%	72%	28%
	PGY-3	8	68%	75%	7%
Tracheostomy module (*N* = 18)	MS-4	6	60%	77%	17%
	PGY-1	3	73%	80%	7%
	PGY-2	5	80%	100%	20%
	PGY-3	4	95%	95%	0%

Abbreviations: MS-4, fourth-year medical student; PGY-1, first-year resident; PGY-2, second-year resident; PGY-3, third-year resident.

Discussion

We have created and implemented four case-based learning modules in our pediatric pulmonary elective, which have been well received by trainees at our institution. Trainees have shown an improvement in mean pos-test scores across all modules, suggesting that online, case-based learning is a helpful adjunct in our pediatric pulmonary elective. As there is variability in the pulmonary diagnoses encountered by trainees during the elective, the modules provide a standardized level of exposure to common conditions they are likely to face during their training and in clinical practice.

Case-based learning is a productive way of presenting knowledge through clinical application and discussion. This type of learning has been shown to enhance clinical knowledge, improve clinical skills, and improve patient outcomes.[5-8] Overall, there was an improvement in knowledge gained across all four modules. The highest increase was seen in the OSA module, which could be due to lack of exposure to sleep disorders during

Table 2. Feedback Received From Trainees

Theme	Quotations
Satisfaction with the cases and relevance to the elective	• "I found them to be overall very helpful and a great supplement to my learning so far on this rotation."<br>• "They were very informative and presented very useful information in an easily digestible way."<br>• "The modules were great! They were a very helpful source during the rotation, and I really enjoyed how interactive they are. Everything flowed very well, and I felt that there was sufficient information for each subject. I think all future med students and residents will enjoy this learning resource!"
Exposure to topics they usually do not see	• "I loved the tracheostomy module as this was a new topic for me in terms of seeing infants with tracheostomies, so I learned a lot from this one. I also loved the videos in the module."<br>• "I appreciated the sleep apnea module because we do not really get any exposure to sleep related disorders and the information presented felt new to me. The tracheostomy module was also very helpful because it went over the basics—which we also don't get in residency, and we are just expected to know."
Supplement and increased knowledge base	• "Although I do have a lot of exposure to asthma and chronic cough, learning about how to organize the differential and the right questions to ask were helpful. I also did not know the proper technique in administering an MDI with spacer and DPI and learned a lot and will now be able to review proper technique with my patients."<br>• "The videos on proper techniques for administering asthma medications were so helpful. I did not understand the concept of priming an asthma inhaler until I watched these videos."<br>• "I felt more comfortable taking care of tracheostomy patients while on my PICU rotation after doing these modules."
Areas for improvement	• "[In the sleep module] in patients with Trisomy 21 I think it might be helpful to include a link to the AAP guidelines on specific health care maintenance for this population... it might be a nice introduction for residents who may not know that there is a resource describing a whole set of specific health considerations for T21 patients."<br>• "I found myself struggling most with the classification of asthma severity/control and it would be helpful to allow learners to reference the chart as a resource."<br>• "I think it would have been helpful to include information about how to manage asthma exacerbations in various settings (e.g. in the outpatient setting vs. ED, vs. floor/ICU) with a discussion on steroids, and more advanced bronchodilation therapies (e.g. aminophylline, ketamine, etc.)."

Abbreviations: AAP, American Academy of Pediatrics; DPI, dry powder inhaler; ED, emergency department; MDI, metered dose inhaler; PICU, pediatric intensive care unit.

Copyright © 2024 Vaziri et al. This is an open-access publication distributed under the terms of the Creative Commons Attribution license.

pediatric residency. When the results were grouped by training level, third-year residents had the smallest average improvement while second-year residents had the largest. This may suggest that while the modules are effective across all training levels, the greatest benefit was for medical students and first- and second-year trainees.

While there were several benefits noted with the modules, we did identify a few limitations. Although we did make the modules required, there was a variable level of engagement amongst trainees with the different cases, and some trainees did not complete all the modules. Additionally, while we assessed knowledge with pre- and posttest surveys, a disadvantage of this design is the possibility of participants benefiting from practice effect. Participants may remember or learn from the pretest questions, inflating posttest scores and making it difficult to determine the true effectiveness of the intervention. We also did not formally assess attitudes and perceptions towards the modules.

We plan to incorporate changes based on feedback received from learners and incorporate the modules into our institution's general pediatric curriculum to standardize pulmonary education for more learners. We also plan to create more pediatric pulmonary, case-based, interactive modules on topics of high demand, such as cystic fibrosis and basics of mechanical ventilation.

Respiratory conditions are the most common reasons for pediatrician visits. Depending on practice location, access to subspecialty care may be limited and frontline practitioners may have to independently manage children with these respiratory conditions. Knowledge and familiarity with these presentations is critical for general pediatricians. Case-based, interactive modules are an effective way to improve knowledge amongst trainees.

Appendices

A. Asthma Module folder
B. Tracheostomy Module folder
C. Chronic Cough Module folder
D. Obstructive Sleep Apnea Module folder
E. Posttest Questions.docx
F. Feedback.docx

All appendices are peer reviewed as integral parts of the Original Publication.

Sanaz Vaziri, MD: Third-Year Fellow, Department of Pediatric Pulmonology, University of California, San Francisco, School of Medicine; ORCID: https://orcid.org/0000-0002-5842-5082

Fatima Neemuchwala, MD: Assistant Professor, Department of Pediatric Pulmonology, University of California, San Francisco, School of Medicine

Janet Lim, MD: Assistant Professor, Department of Pediatric Pulmonology, Loma Linda University School of Medicine

Marilynn Chan, MD: Physician, Department of Pediatric Pulmonology, Kaiser Permanente

Disclosures:
None to report.

Funding/Support:
None to report.

Ethical Approval:
Reported as not applicable.

References

1. Finley CR, Chan DS, Garrison S, et al. What are the most common conditions in primary care? Systematic review. *Can Fam Physician*. 2018;64(11):832-840.
2. Freed GL, Dunham KM, Switalski KE, Jones MD Jr, McGuinness GA; Research Advisory Committee of the American Board of Pediatrics. Recently trained general pediatricians: perspectives on residency training and scope of practice. *Pediatrics*. 2009;123(suppl 1):S38-S43. https://doi.org/10.1542/peds.2008-1578j
3. Charap MH, Levin RI, Pearlman RE, Blaser MJ. Internal medicine residency training in the 21st century: aligning requirements with professional needs. *Am J Med*. 2005;118(9):1042-1046. https://doi.org/10.1016/j.amjmed.2005.06.009
4. Feifer C, Mora A, White B, Barnett BP. Challenges to improving chronic disease care and training in residencies. *Acad Med*. 2006;81(8):696-701. https://doi.org/10.1097/00001888-200608000-00004
5. Choules AP. The use of elearning in medical education: a review of the current situation. *Postgrad Med J*. 2007;83(978):212-216. https://doi.org/10.1136/pgmj.2006.054189
6. Fransen F, Martens H, Nagtzaam I, Heeneman S. Use of e-learning in clinical clerkships: effects on acquisition of dermatological knowledge and learning processes. *Int J Med Educ*. 2018;9:11-17. https://doi.org/10.5116/ijme.5a47.8ab0
7. Morgulis Y, Kumar RK, Lindeman R, Velan GM. Impact on learning of an e-learning module on leukaemia: a randomised controlled trial. *BMC Med Educ*. 2012;12:36. https://doi.org/10.1186/1472-6920-12-36

Copyright © 2024 Vaziri et al. This is an open-access publication distributed under the terms of the Creative Commons Attribution license.

8. Mallin M, Schlein S, Doctor S, Stroud S, Dawson M, Fix M. A survey of the current utilization of asynchronous education among emergency medicine residents in the United States. *Acad Med*. 2014;89(4):598-601. https://doi.org/10.1097/ACM.0000000000000170
9. Khan EK, Liptzin DR, Baker J, Meier M, Baker CD, Lockspeiser TM. Pediatric resident education in pulmonary (PREP): a subspecialty preparatory boot camp curriculum for pediatric residents. *MedEdPORTAL*. 2021;17:11066. https://doi.org/10.15766/mep_2374-8265.11066
10. Johnston M, Bridges T, Palen B, Parsons E, Wemple M, Adamson R. Hands-on pulmonary curriculum: interactive learning sessions on oxygen delivery, spirometry, positive airway pressure devices, tracheostomy, and thoracostomy tubes. *MedEdPORTAL*. 2017;13:10633. https://doi.org/10.15766/mep_2374-8265.10633
11. Gill P, Kapusta M, Gerdung C, Pinsk M, Duff J, Grewal S. PedsCases—a learning module of pediatric emergency medicine cases for medical students. *MedEdPORTAL*. 2010;6:8191. https://doi.org/10.15766/mep_2374-8265.8191

Received: June 14, 2024
Accepted: August 22, 2024
Published: December 6, 2024

Copyright © 2024 Vaziri et al. This is an open-access publication distributed under the terms of the Creative Commons Attribution license.
