# Supplementary material for: Development and Implementation of a Pediatric Pulmonary–Focused Active Learning Curriculum
Source: MedEdPORTAL. 2024 Dec 6;20:11470. doi: 10.15766/mep_2374-8265.11470 (PMC11621239; doi:10.15766/mep_2374-8265.11470)
Supplement: Supplementary file 1 — Asthma Module folderTracheostomy Module folderChronic Cough Module folderObstructive Sleep Apnea Module folderPosttest Questions.docxFeedback.docx [file mep_2374-8265.11470-s001.zip › A. Asthma Module/scormcontent/index.html]

Nice and Wheezy\*
