# Supplementary figures and images for: Development and Implementation of a Pediatric Pulmonary–Focused Active Learning Curriculum
Source: MedEdPORTAL. 2024 Dec 6;20:11470. doi: 10.15766/mep_2374-8265.11470 (PMC11621239; doi:10.15766/mep_2374-8265.11470)

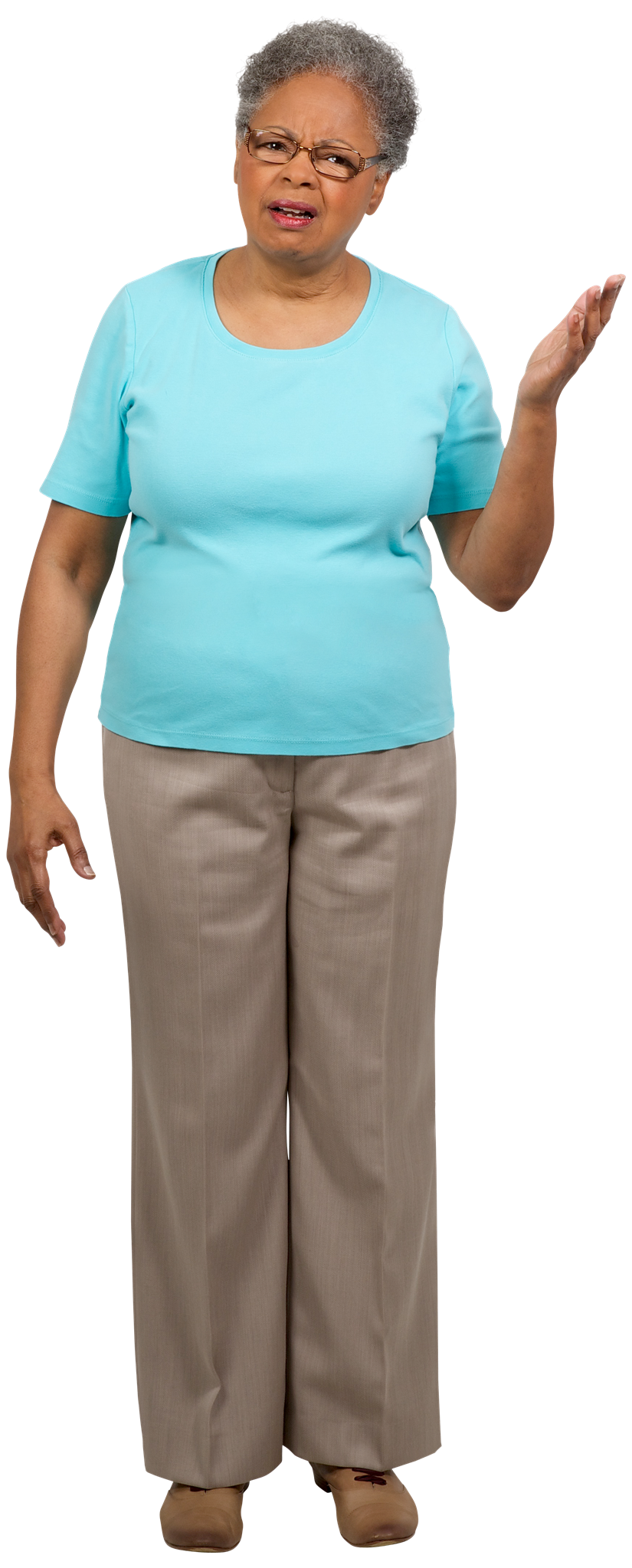

Supplement: Supplementary file 1 — Asthma Module folderTracheostomy Module folderChronic Cough Module folderObstructive Sleep Apnea Module folderPosttest Questions.docxFeedback.docx [file mep_2374-8265.11470-s001.zip › A. Asthma Module/scormcontent/assets/5Q6NOg3u-I-SeYUJ_209_full.png]

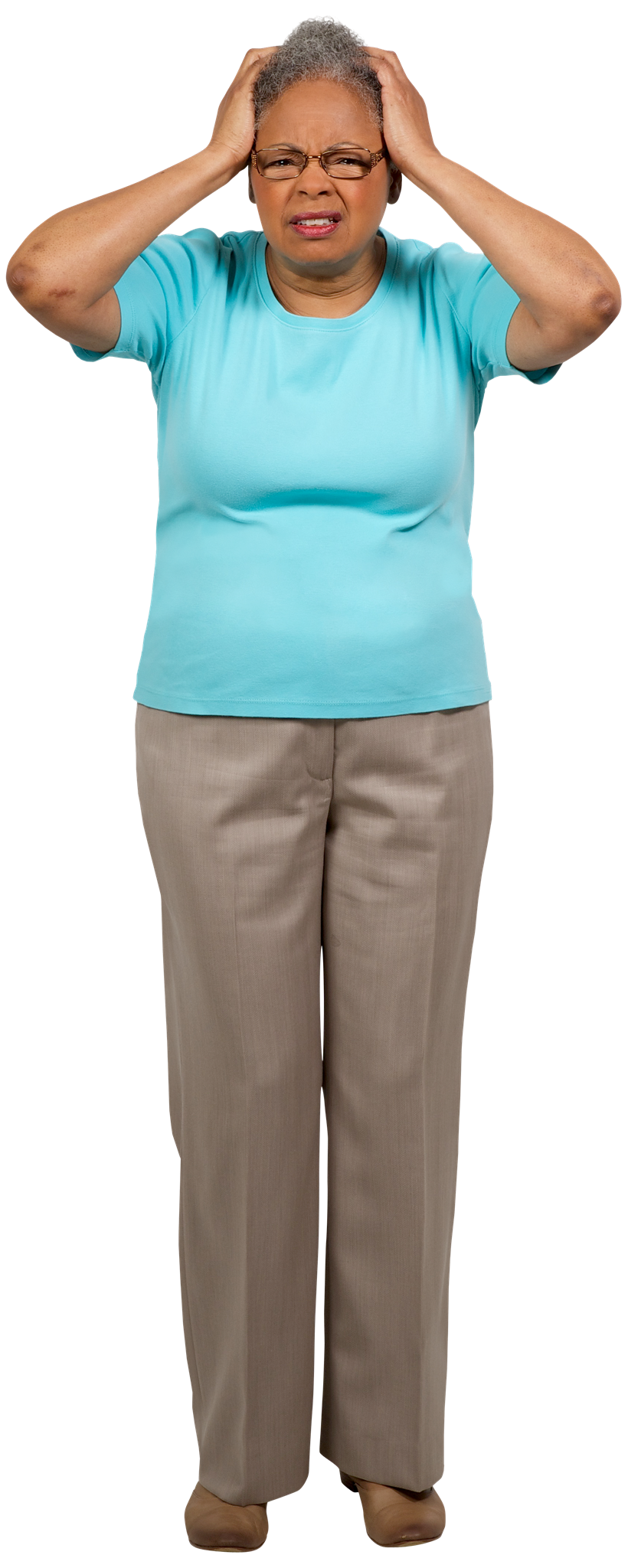

Supplement: Supplementary file 1 — Asthma Module folderTracheostomy Module folderChronic Cough Module folderObstructive Sleep Apnea Module folderPosttest Questions.docxFeedback.docx [file mep_2374-8265.11470-s001.zip › A. Asthma Module/scormcontent/assets/AAyUCKL6tPvB0rZJ_216_full.png]

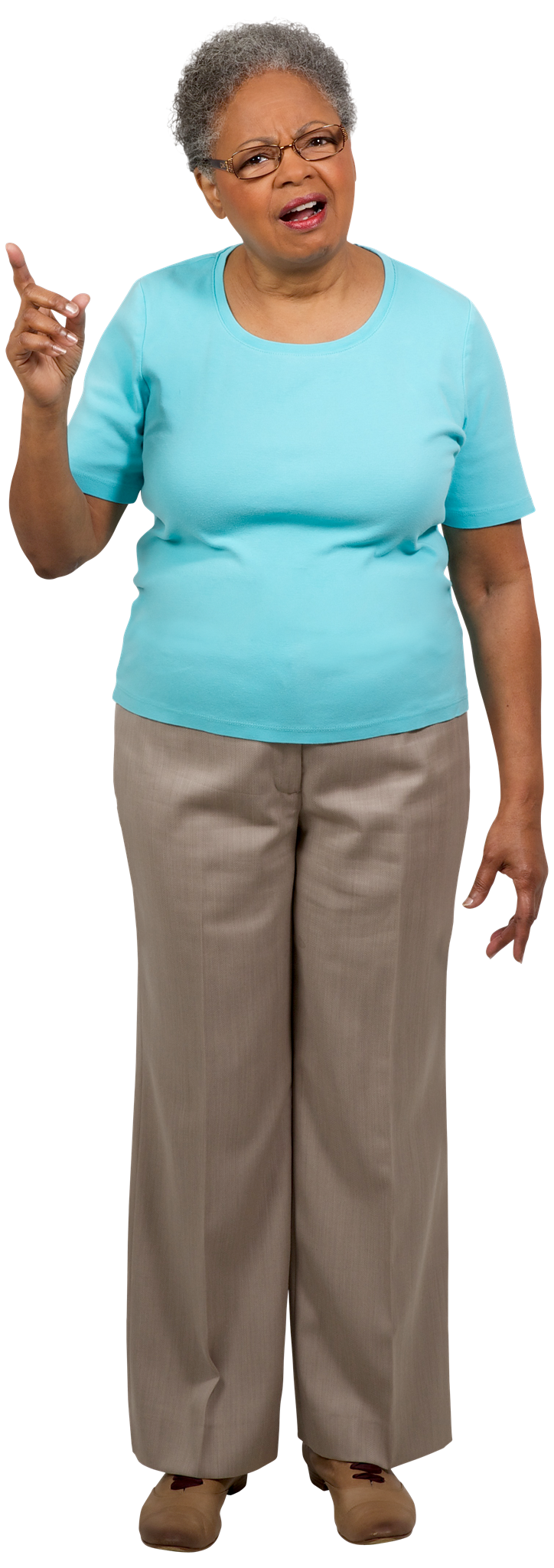

Supplement: Supplementary file 1 — Asthma Module folderTracheostomy Module folderChronic Cough Module folderObstructive Sleep Apnea Module folderPosttest Questions.docxFeedback.docx [file mep_2374-8265.11470-s001.zip › A. Asthma Module/scormcontent/assets/aqeBm99qENEtPcxh_169_full.png]

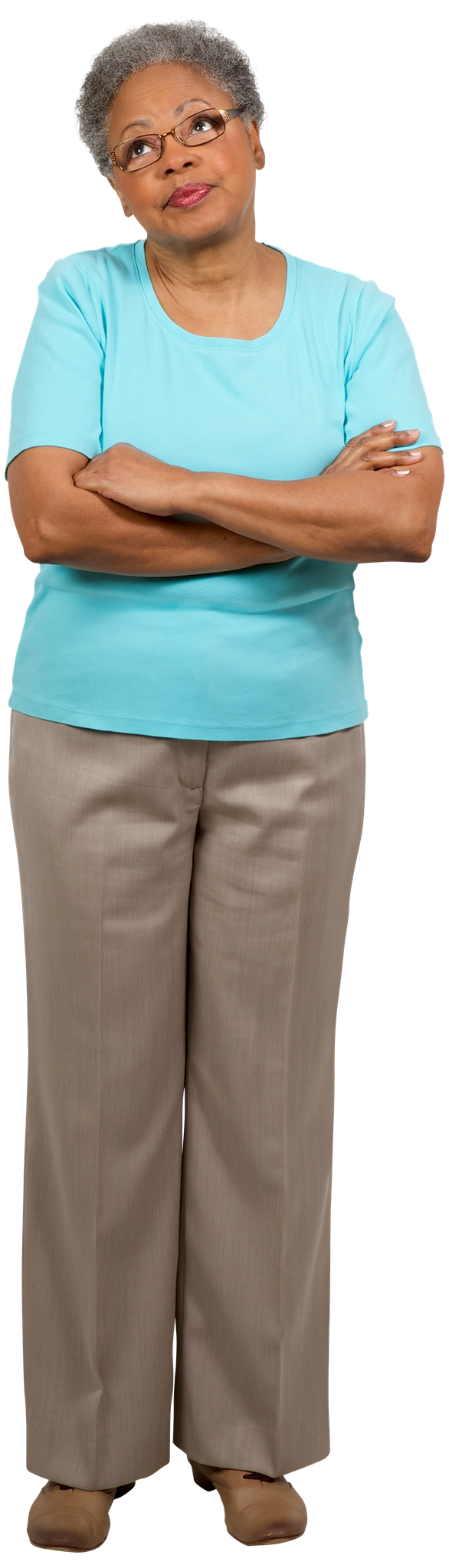

Supplement: Supplementary file 1 — Asthma Module folderTracheostomy Module folderChronic Cough Module folderObstructive Sleep Apnea Module folderPosttest Questions.docxFeedback.docx [file mep_2374-8265.11470-s001.zip › A. Asthma Module/scormcontent/assets/B9JsJ-LXoXfaRI3G_189_full.png]

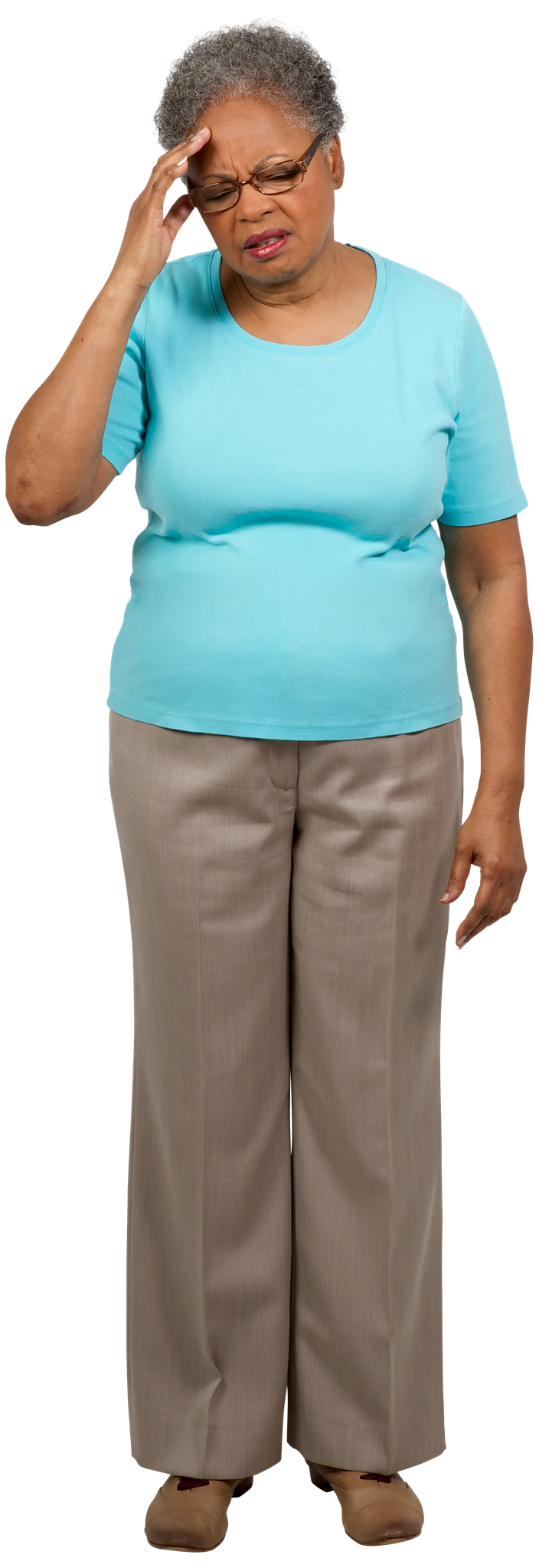

Supplement: Supplementary file 1 — Asthma Module folderTracheostomy Module folderChronic Cough Module folderObstructive Sleep Apnea Module folderPosttest Questions.docxFeedback.docx [file mep_2374-8265.11470-s001.zip › A. Asthma Module/scormcontent/assets/BLgqtqYclv4JlM5l_185_full.png]

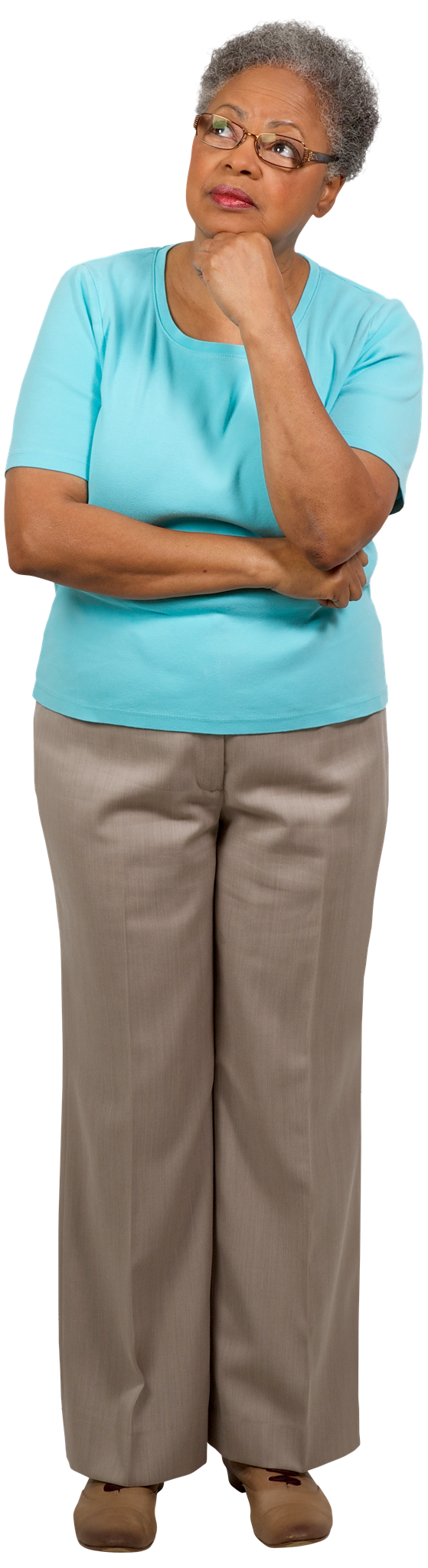

Supplement: Supplementary file 1 — Asthma Module folderTracheostomy Module folderChronic Cough Module folderObstructive Sleep Apnea Module folderPosttest Questions.docxFeedback.docx [file mep_2374-8265.11470-s001.zip › A. Asthma Module/scormcontent/assets/C0ZL6bUVGuiFSuOo_219_full.png]

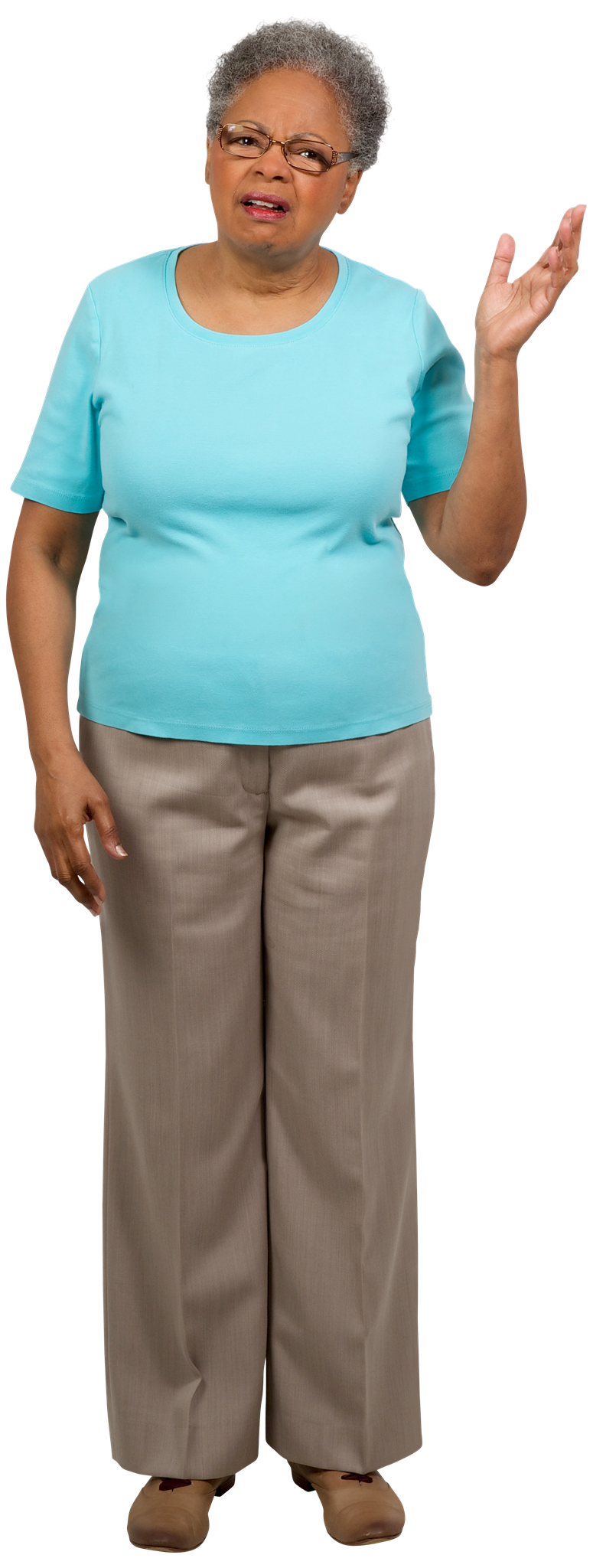

Supplement: Supplementary file 1 — Asthma Module folderTracheostomy Module folderChronic Cough Module folderObstructive Sleep Apnea Module folderPosttest Questions.docxFeedback.docx [file mep_2374-8265.11470-s001.zip › A. Asthma Module/scormcontent/assets/EVcVIJE_QHU7XRpx_180_full.png]

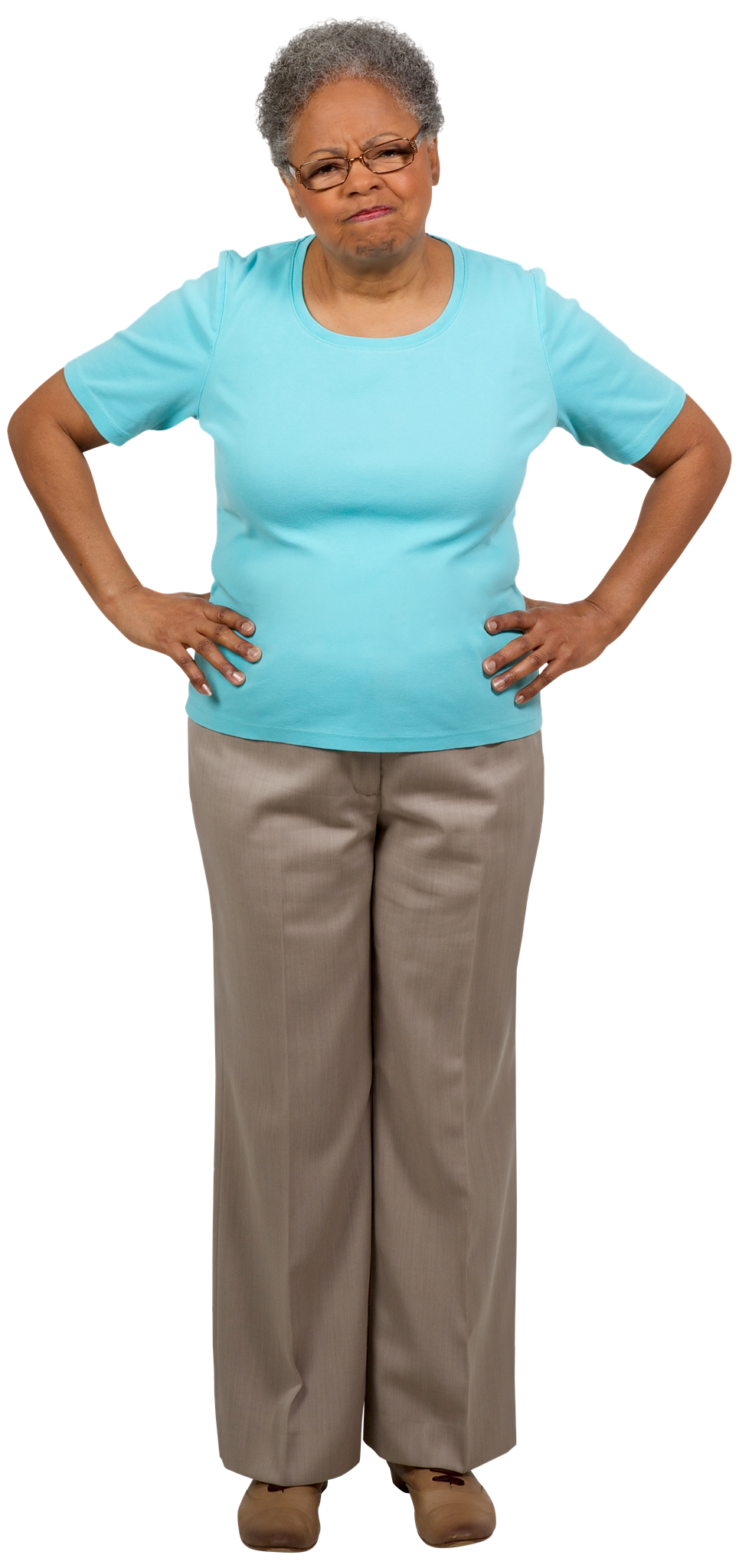

Supplement: Supplementary file 1 — Asthma Module folderTracheostomy Module folderChronic Cough Module folderObstructive Sleep Apnea Module folderPosttest Questions.docxFeedback.docx [file mep_2374-8265.11470-s001.zip › A. Asthma Module/scormcontent/assets/f6DJNol-G_4AX-yr_215_full.png]

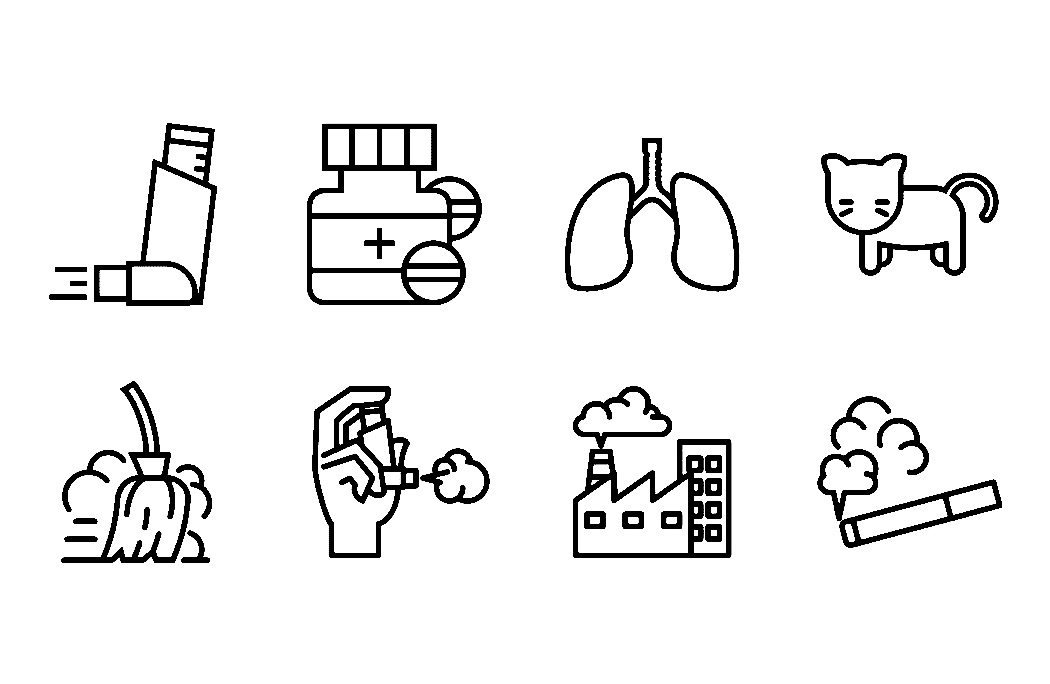

Supplement: Supplementary file 1 — Asthma Module folderTracheostomy Module folderChronic Cough Module folderObstructive Sleep Apnea Module folderPosttest Questions.docxFeedback.docx [file mep_2374-8265.11470-s001.zip › A. Asthma Module/scormcontent/assets/HKp7xCpC98F5BTeY.png]

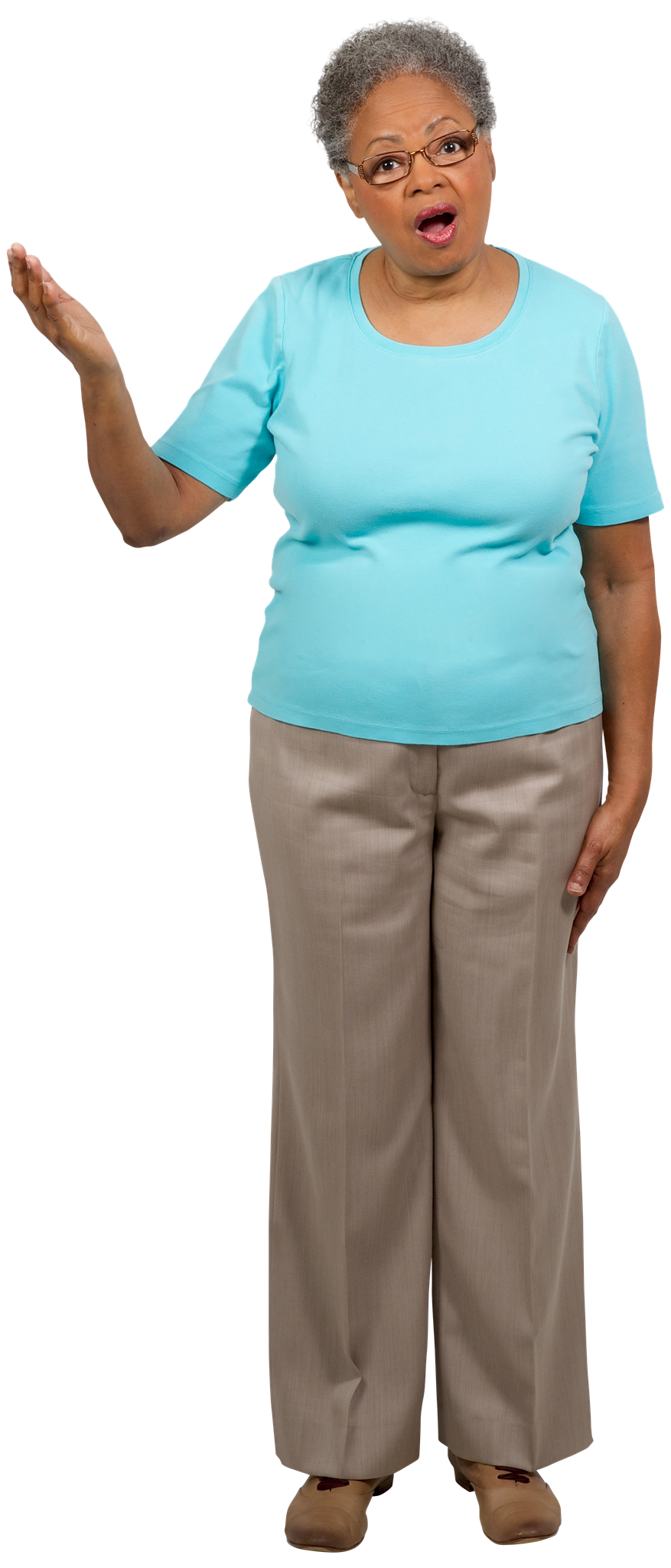

Supplement: Supplementary file 1 — Asthma Module folderTracheostomy Module folderChronic Cough Module folderObstructive Sleep Apnea Module folderPosttest Questions.docxFeedback.docx [file mep_2374-8265.11470-s001.zip › A. Asthma Module/scormcontent/assets/HqAs6EJXUt712yR2_158_full.png]

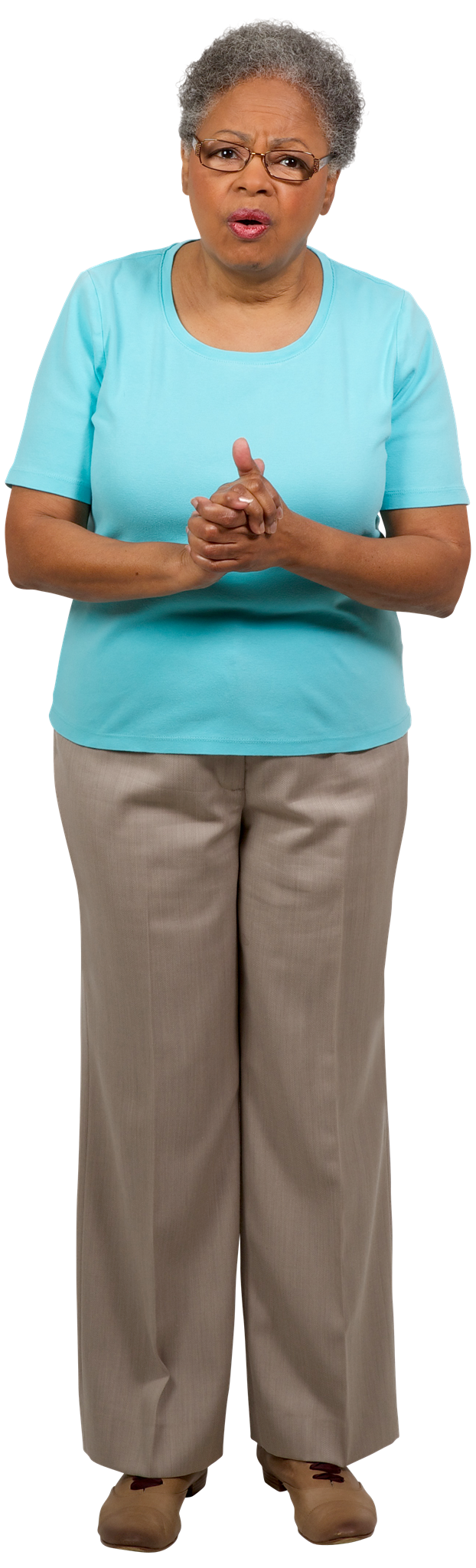

Supplement: Supplementary file 1 — Asthma Module folderTracheostomy Module folderChronic Cough Module folderObstructive Sleep Apnea Module folderPosttest Questions.docxFeedback.docx [file mep_2374-8265.11470-s001.zip › A. Asthma Module/scormcontent/assets/I_uj6qpDJb-VVySI_198_full.png]

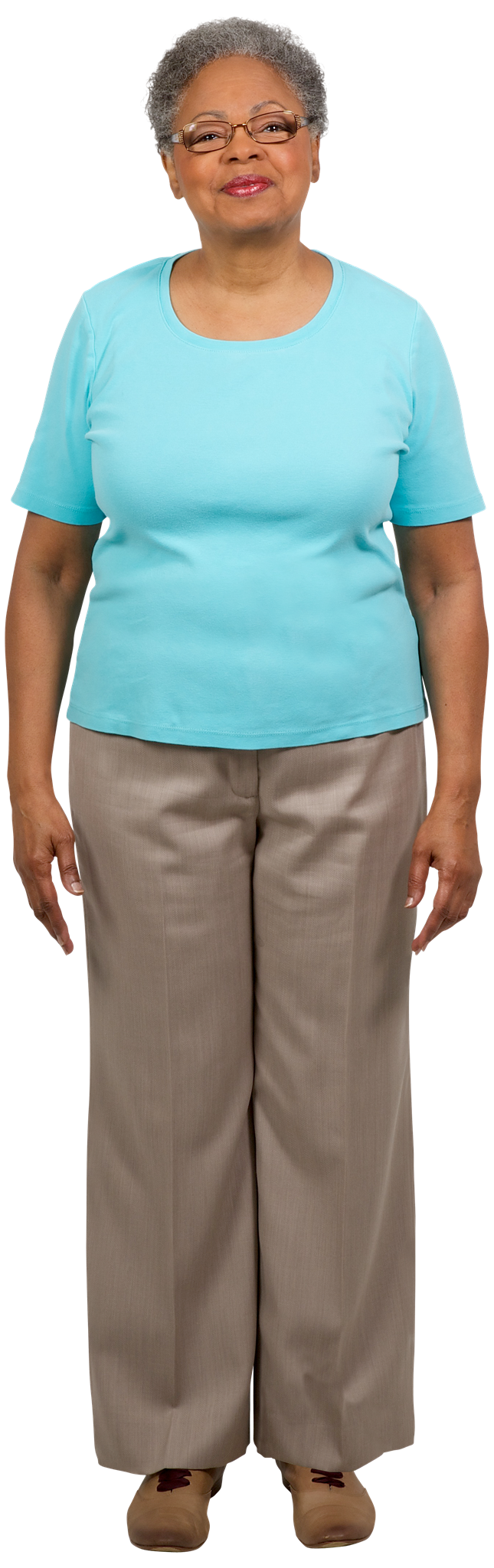

Supplement: Supplementary file 1 — Asthma Module folderTracheostomy Module folderChronic Cough Module folderObstructive Sleep Apnea Module folderPosttest Questions.docxFeedback.docx [file mep_2374-8265.11470-s001.zip › A. Asthma Module/scormcontent/assets/IR-7kdTENIuYgMli_106_full.png]

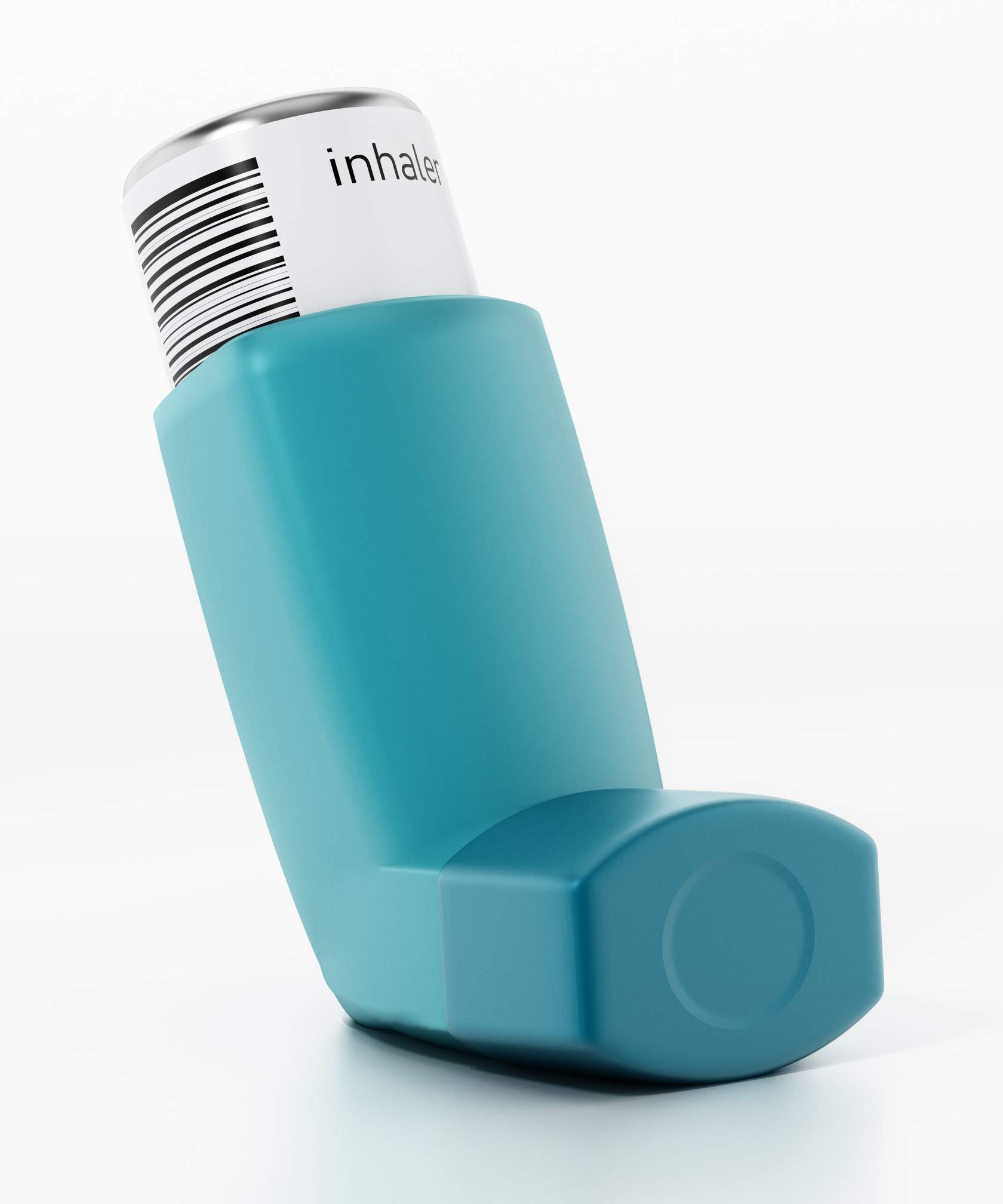

Supplement: Supplementary file 1 — Asthma Module folderTracheostomy Module folderChronic Cough Module folderObstructive Sleep Apnea Module folderPosttest Questions.docxFeedback.docx [file mep_2374-8265.11470-s001.zip › A. Asthma Module/scormcontent/assets/iStock-1082347064.jpg]

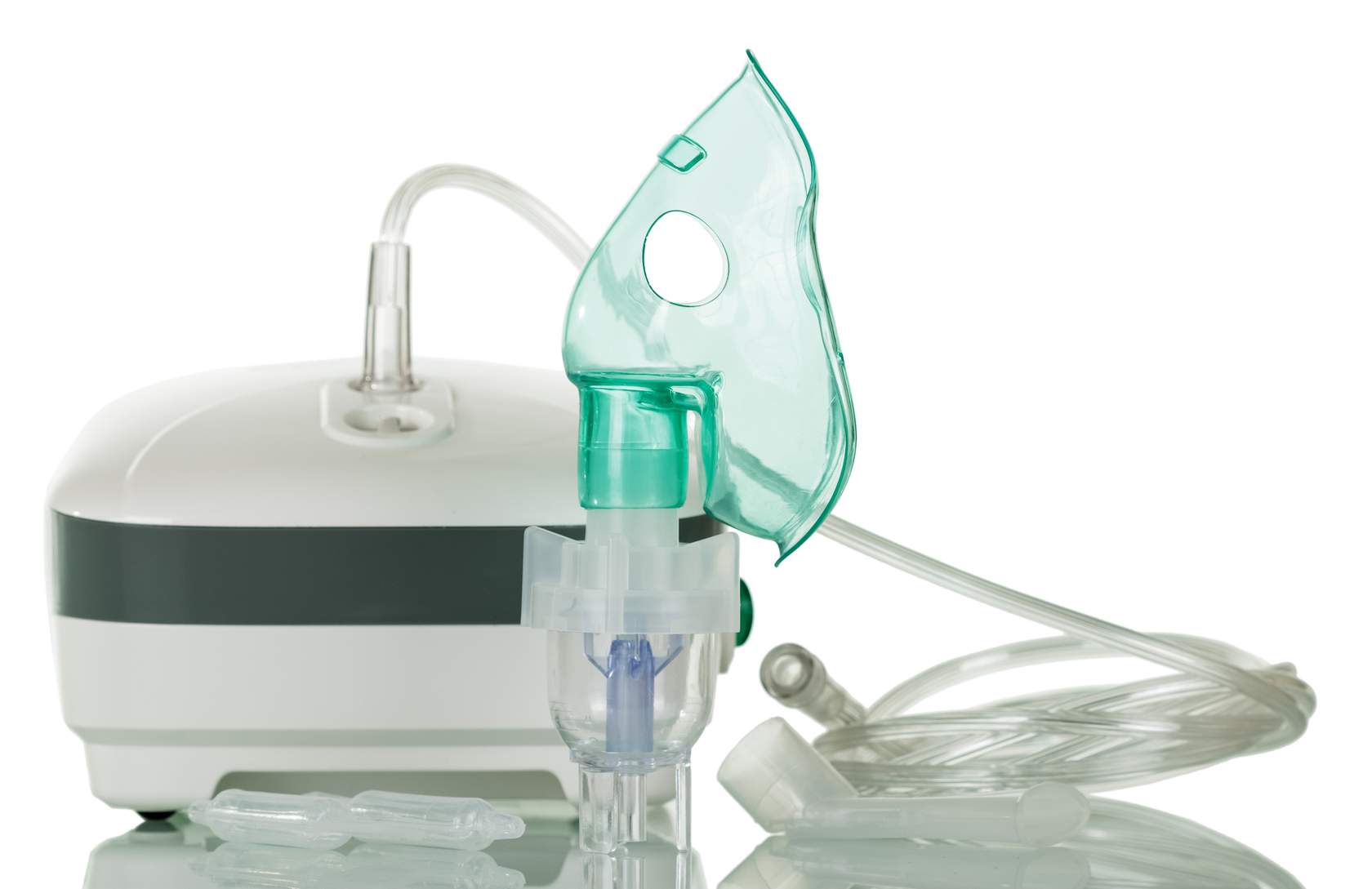

Supplement: Supplementary file 1 — Asthma Module folderTracheostomy Module folderChronic Cough Module folderObstructive Sleep Apnea Module folderPosttest Questions.docxFeedback.docx [file mep_2374-8265.11470-s001.zip › A. Asthma Module/scormcontent/assets/iStock-852092470.jpg]

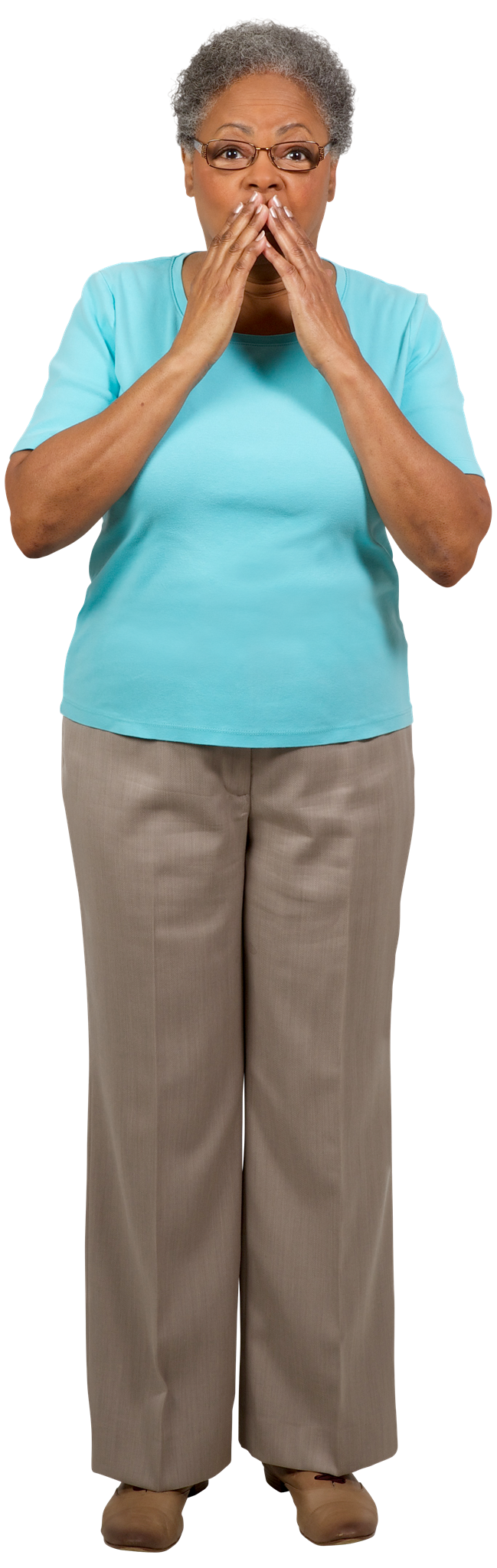

Supplement: Supplementary file 1 — Asthma Module folderTracheostomy Module folderChronic Cough Module folderObstructive Sleep Apnea Module folderPosttest Questions.docxFeedback.docx [file mep_2374-8265.11470-s001.zip › A. Asthma Module/scormcontent/assets/JoZVQSd7JxKhmaTQ_187_full.png]

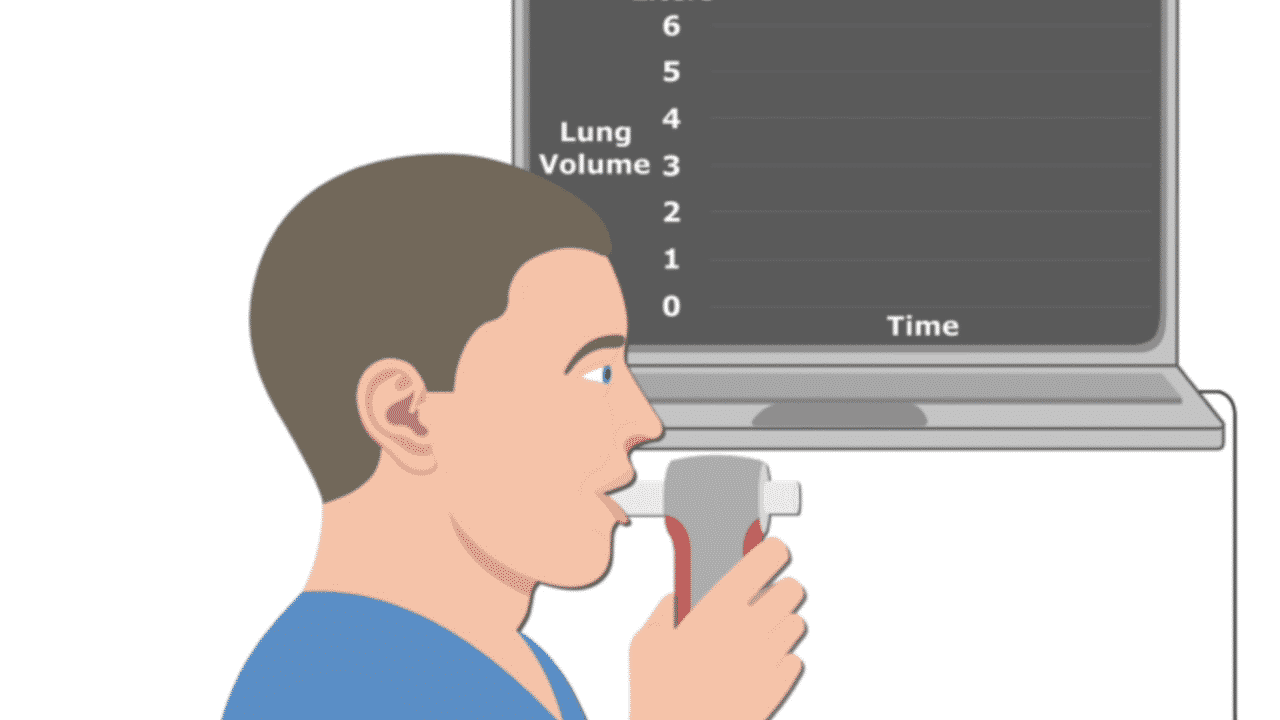

Supplement: Supplementary file 1 — Asthma Module folderTracheostomy Module folderChronic Cough Module folderObstructive Sleep Apnea Module folderPosttest Questions.docxFeedback.docx [file mep_2374-8265.11470-s001.zip › A. Asthma Module/scormcontent/assets/kenshospirometry.png]

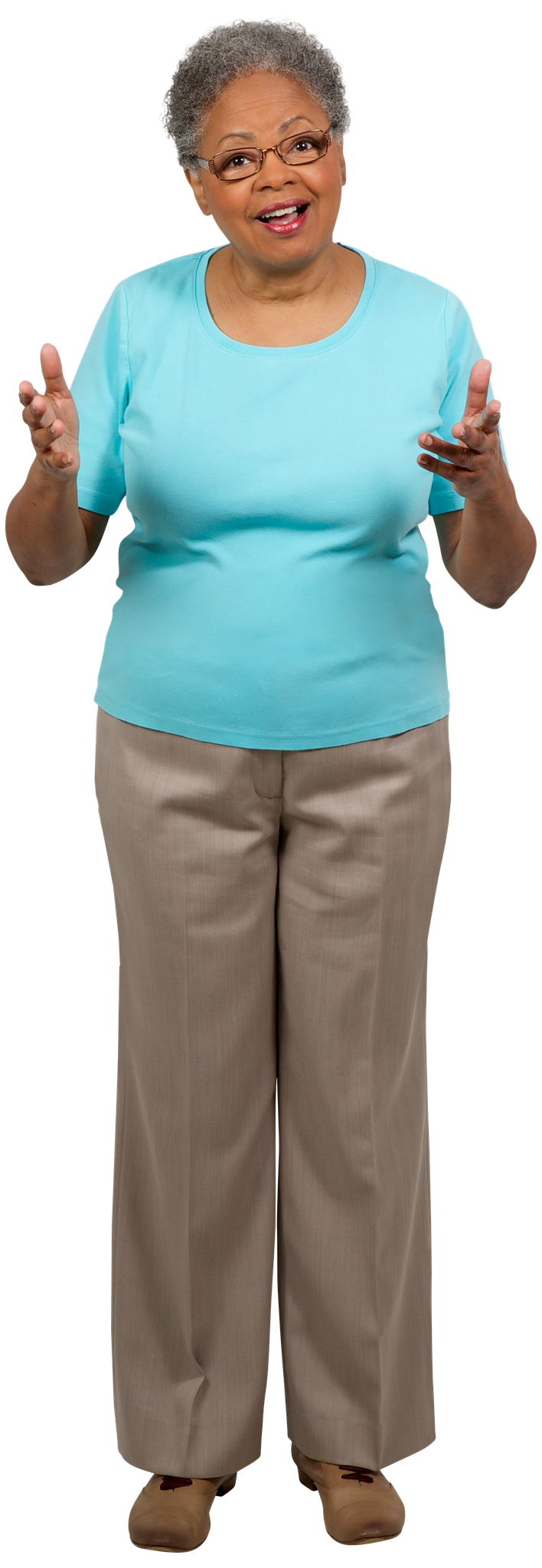

Supplement: Supplementary file 1 — Asthma Module folderTracheostomy Module folderChronic Cough Module folderObstructive Sleep Apnea Module folderPosttest Questions.docxFeedback.docx [file mep_2374-8265.11470-s001.zip › A. Asthma Module/scormcontent/assets/MecG18ubSNNdz9Dn_162_full.png]

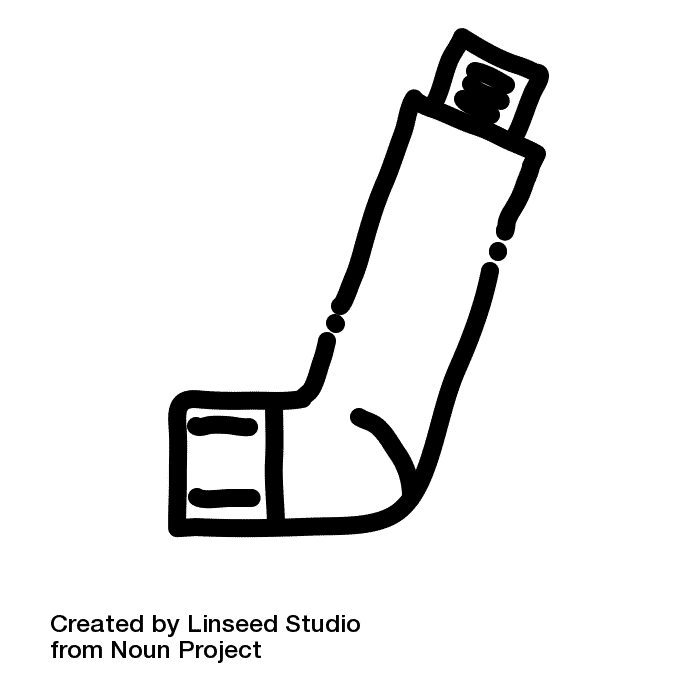

Supplement: Supplementary file 1 — Asthma Module folderTracheostomy Module folderChronic Cough Module folderObstructive Sleep Apnea Module folderPosttest Questions.docxFeedback.docx [file mep_2374-8265.11470-s001.zip › A. Asthma Module/scormcontent/assets/noun_inhaler_1239931.png]

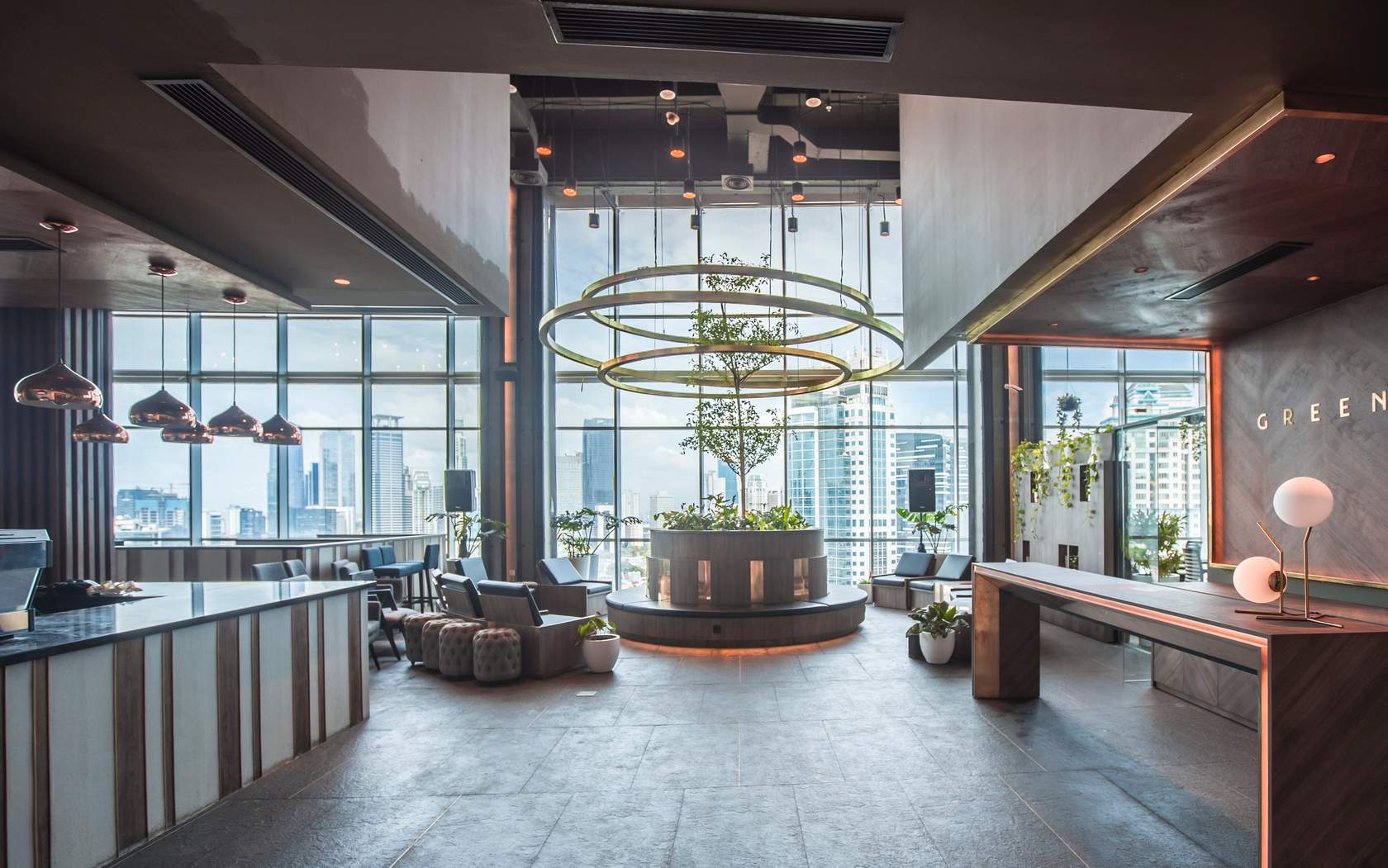

Supplement: Supplementary file 1 — Asthma Module folderTracheostomy Module folderChronic Cough Module folderObstructive Sleep Apnea Module folderPosttest Questions.docxFeedback.docx [file mep_2374-8265.11470-s001.zip › A. Asthma Module/scormcontent/assets/NzBPF8/hotel.jpg]

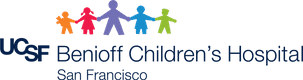

Supplement: Supplementary file 1 — Asthma Module folderTracheostomy Module folderChronic Cough Module folderObstructive Sleep Apnea Module folderPosttest Questions.docxFeedback.docx [file mep_2374-8265.11470-s001.zip › A. Asthma Module/scormcontent/assets/o4AtQ8oYNqcSTWfI.png]

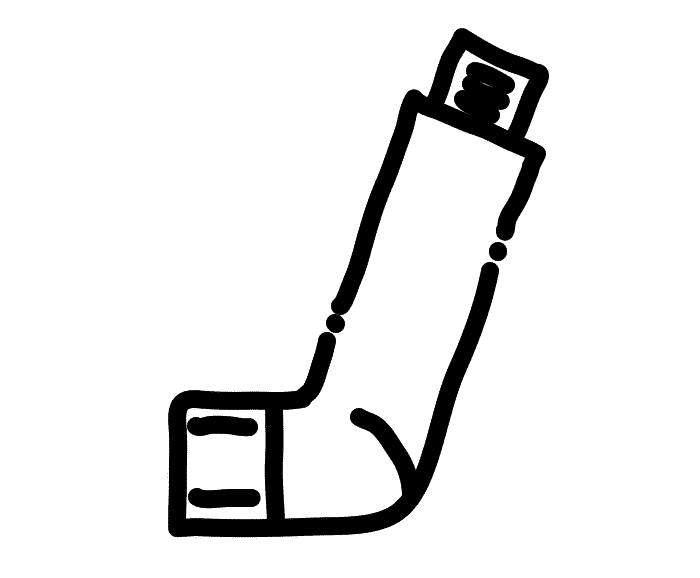

Supplement: Supplementary file 1 — Asthma Module folderTracheostomy Module folderChronic Cough Module folderObstructive Sleep Apnea Module folderPosttest Questions.docxFeedback.docx [file mep_2374-8265.11470-s001.zip › A. Asthma Module/scormcontent/assets/oLvBuEJWLc-Xc8kf.png]

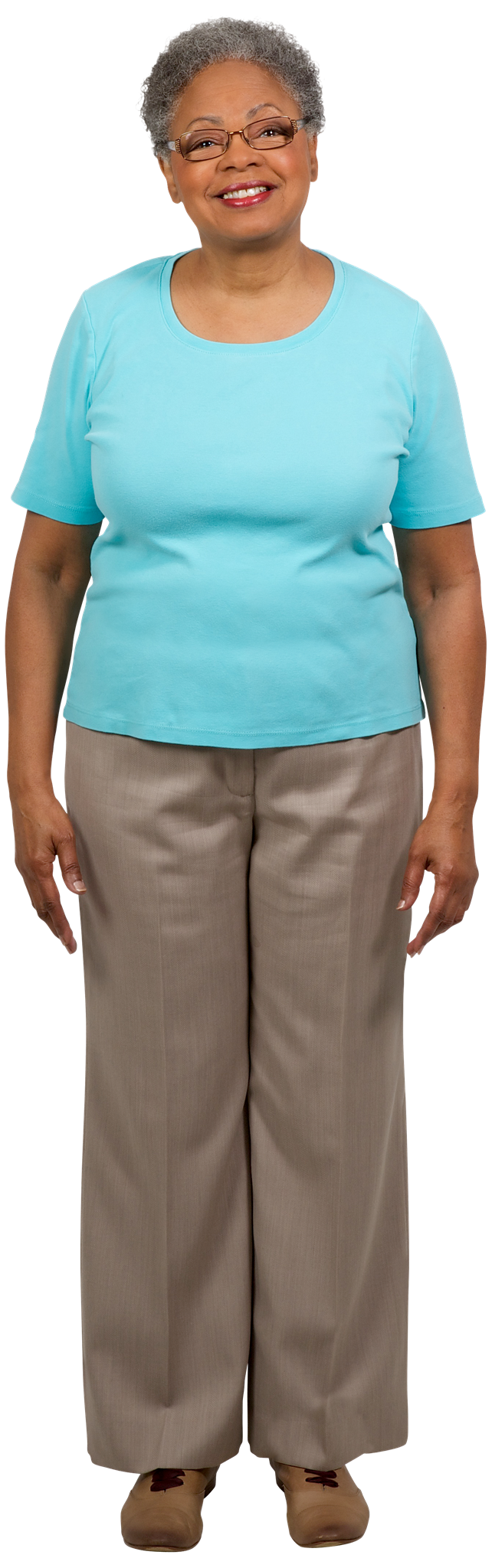

Supplement: Supplementary file 1 — Asthma Module folderTracheostomy Module folderChronic Cough Module folderObstructive Sleep Apnea Module folderPosttest Questions.docxFeedback.docx [file mep_2374-8265.11470-s001.zip › A. Asthma Module/scormcontent/assets/oLZSAbeAuJbvwFCD_107_full.png]

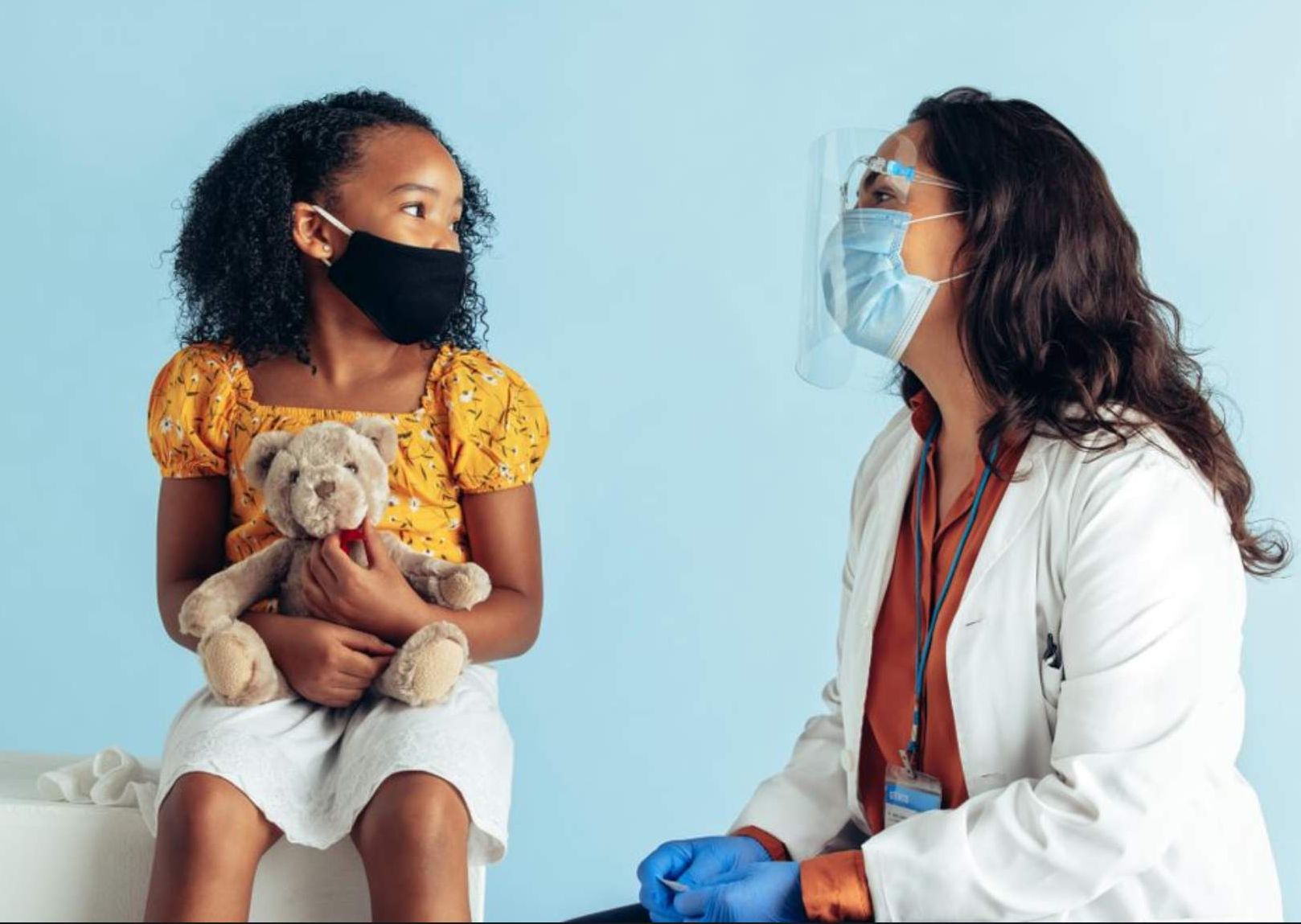

Supplement: Supplementary file 1 — Asthma Module folderTracheostomy Module folderChronic Cough Module folderObstructive Sleep Apnea Module folderPosttest Questions.docxFeedback.docx [file mep_2374-8265.11470-s001.zip › A. Asthma Module/scormcontent/assets/Screen Shot 2021-09-24 at 4.15.40 PM.jpg]

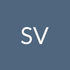

Supplement: Supplementary file 1 — Asthma Module folderTracheostomy Module folderChronic Cough Module folderObstructive Sleep Apnea Module folderPosttest Questions.docxFeedback.docx [file mep_2374-8265.11470-s001.zip › A. Asthma Module/scormcontent/assets/small.png]

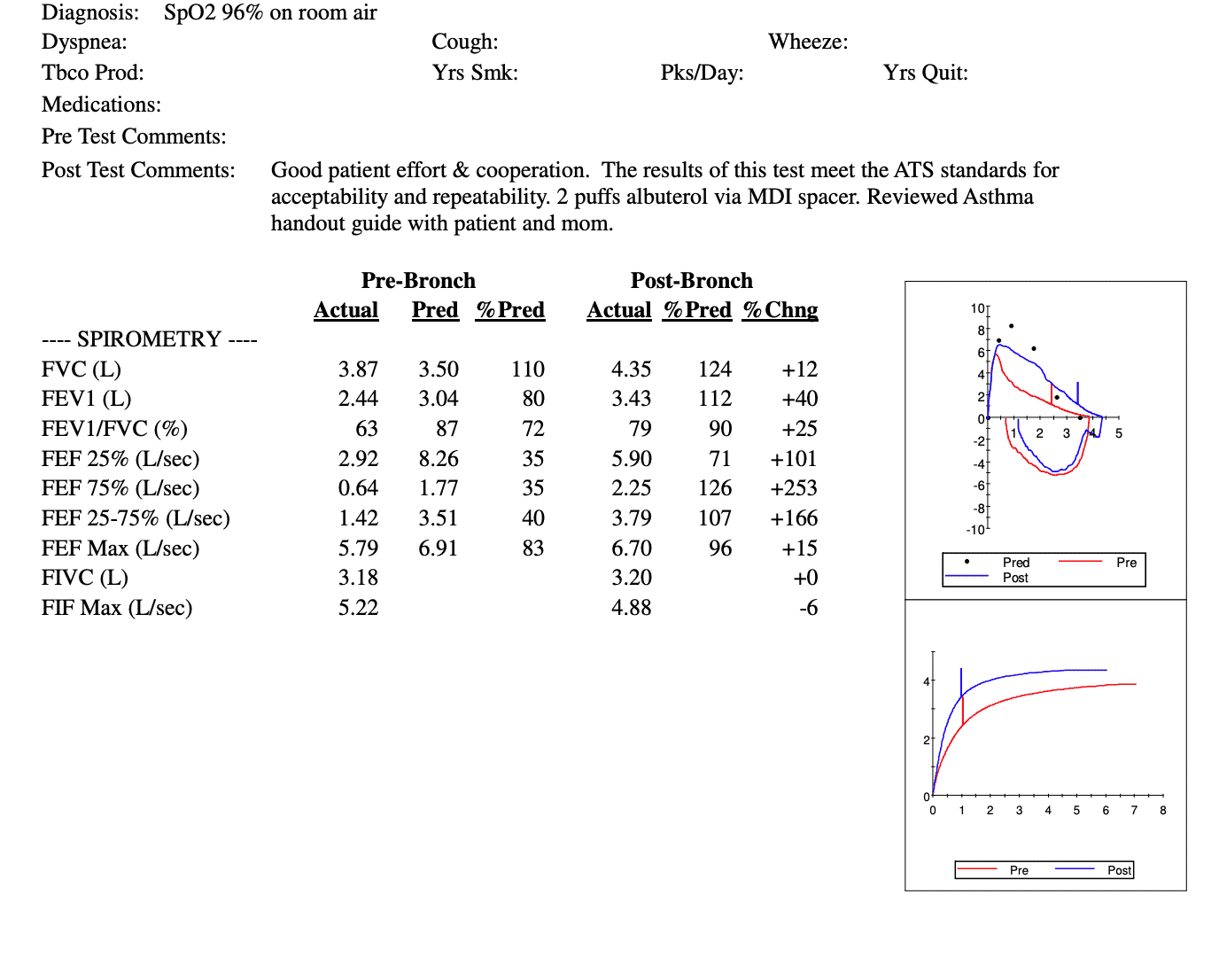

Supplement: Supplementary file 1 — Asthma Module folderTracheostomy Module folderChronic Cough Module folderObstructive Sleep Apnea Module folderPosttest Questions.docxFeedback.docx [file mep_2374-8265.11470-s001.zip › A. Asthma Module/scormcontent/assets/spirometry.png]

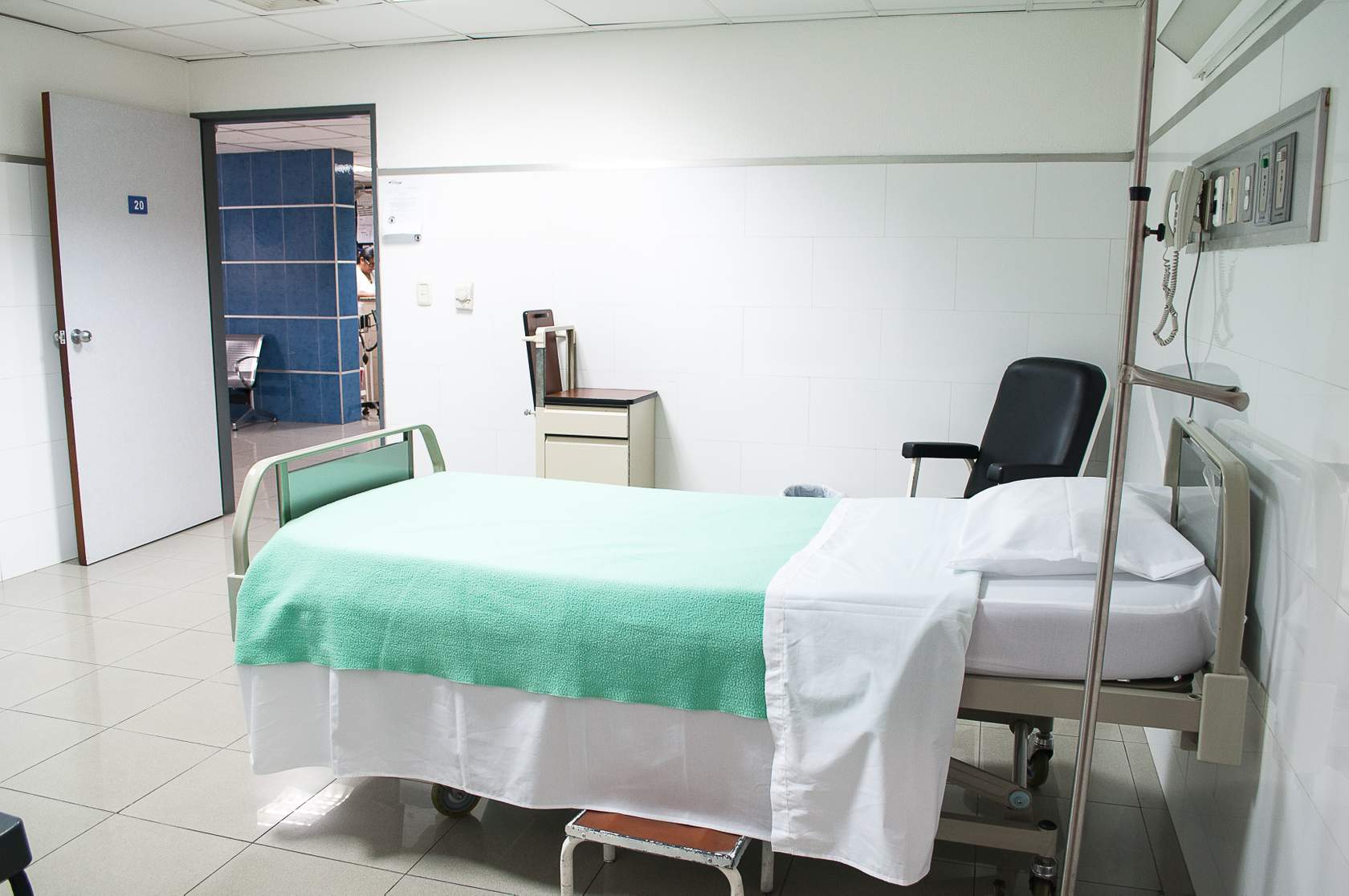

Supplement: Supplementary file 1 — Asthma Module folderTracheostomy Module folderChronic Cough Module folderObstructive Sleep Apnea Module folderPosttest Questions.docxFeedback.docx [file mep_2374-8265.11470-s001.zip › A. Asthma Module/scormcontent/assets/stock-image.jpg]

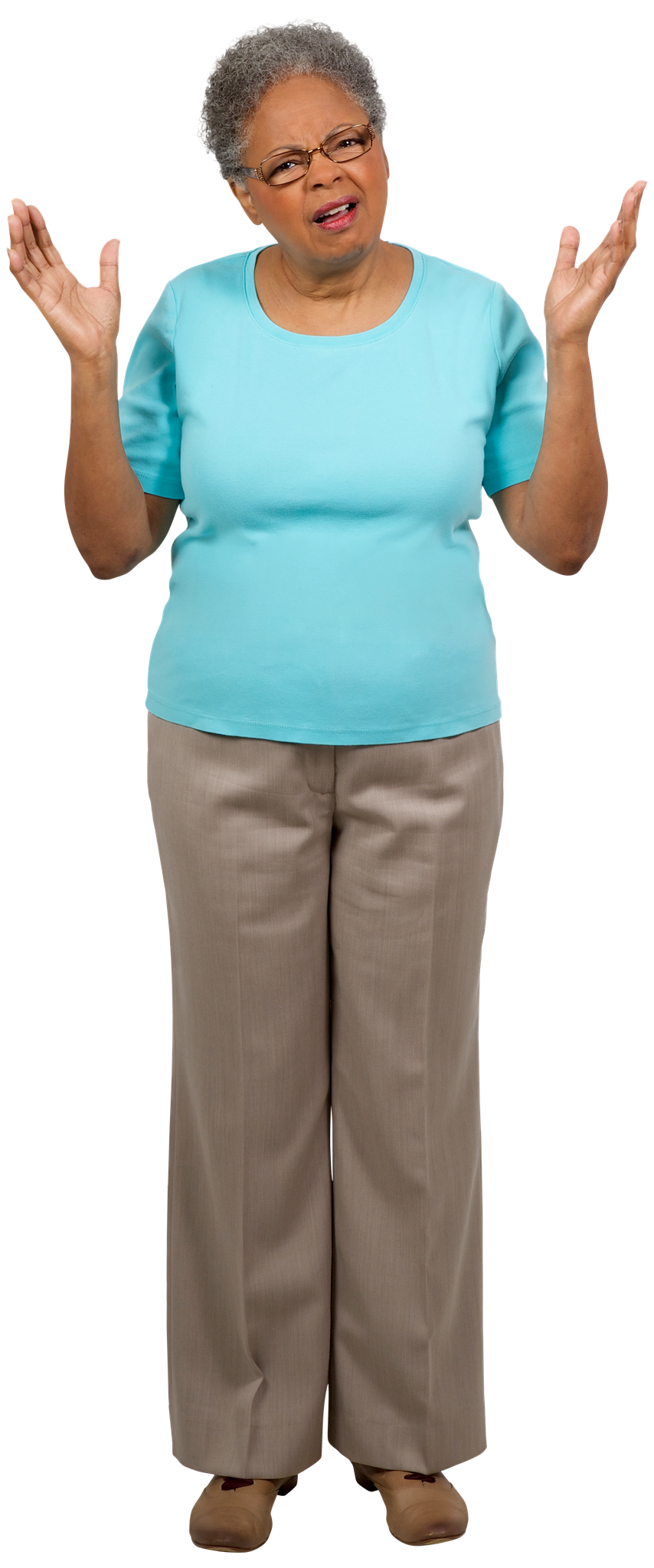

Supplement: Supplementary file 1 — Asthma Module folderTracheostomy Module folderChronic Cough Module folderObstructive Sleep Apnea Module folderPosttest Questions.docxFeedback.docx [file mep_2374-8265.11470-s001.zip › A. Asthma Module/scormcontent/assets/SY1YyjgNohUXUUWV_182_full.png]

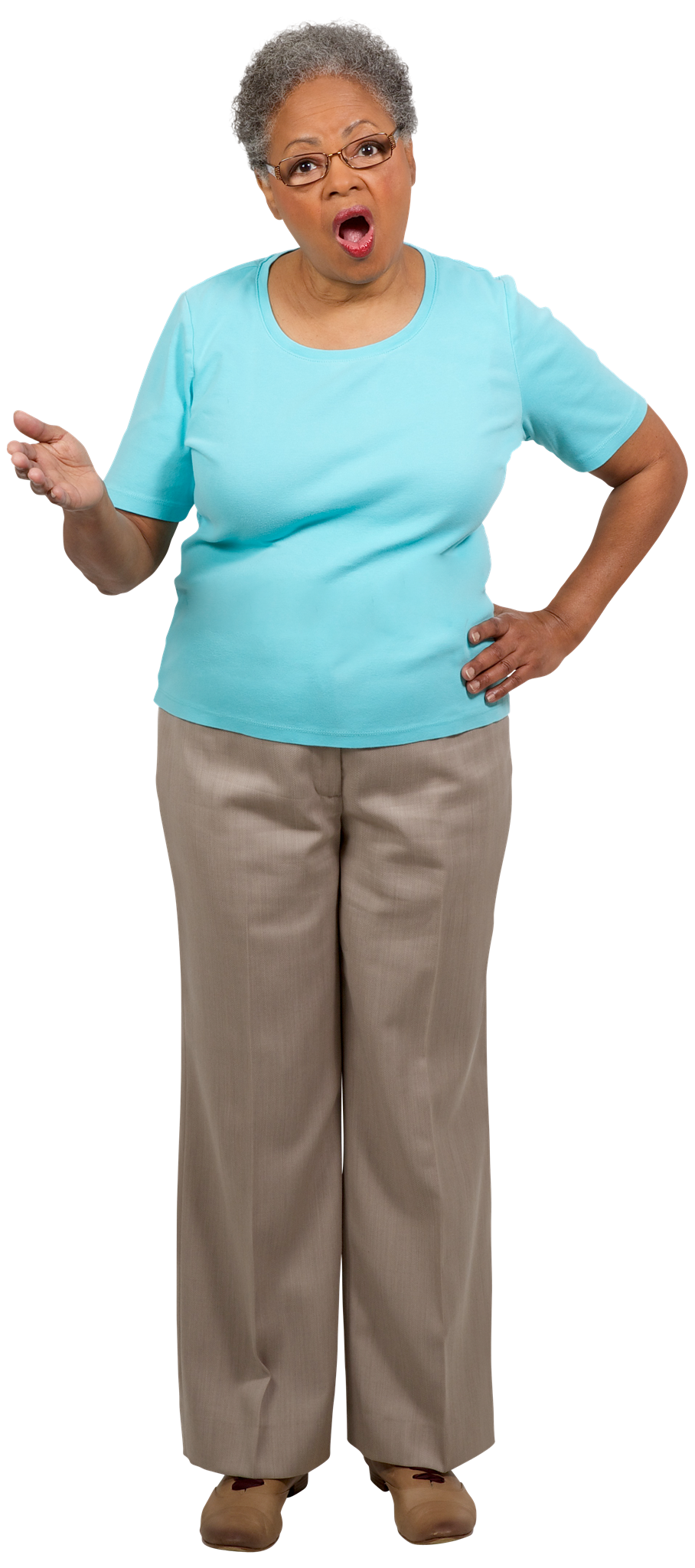

Supplement: Supplementary file 1 — Asthma Module folderTracheostomy Module folderChronic Cough Module folderObstructive Sleep Apnea Module folderPosttest Questions.docxFeedback.docx [file mep_2374-8265.11470-s001.zip › A. Asthma Module/scormcontent/assets/TlJ1_TGtvfo5fGzX_156_full.png]

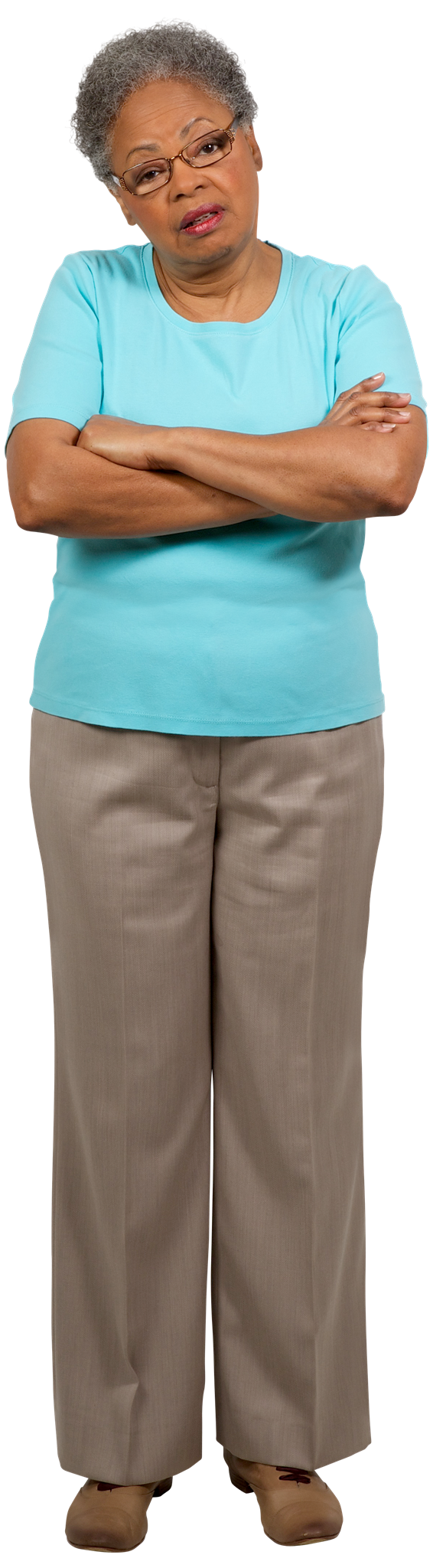

Supplement: Supplementary file 1 — Asthma Module folderTracheostomy Module folderChronic Cough Module folderObstructive Sleep Apnea Module folderPosttest Questions.docxFeedback.docx [file mep_2374-8265.11470-s001.zip › A. Asthma Module/scormcontent/assets/tz4LBTF-y1nULSlu_194_full.png]

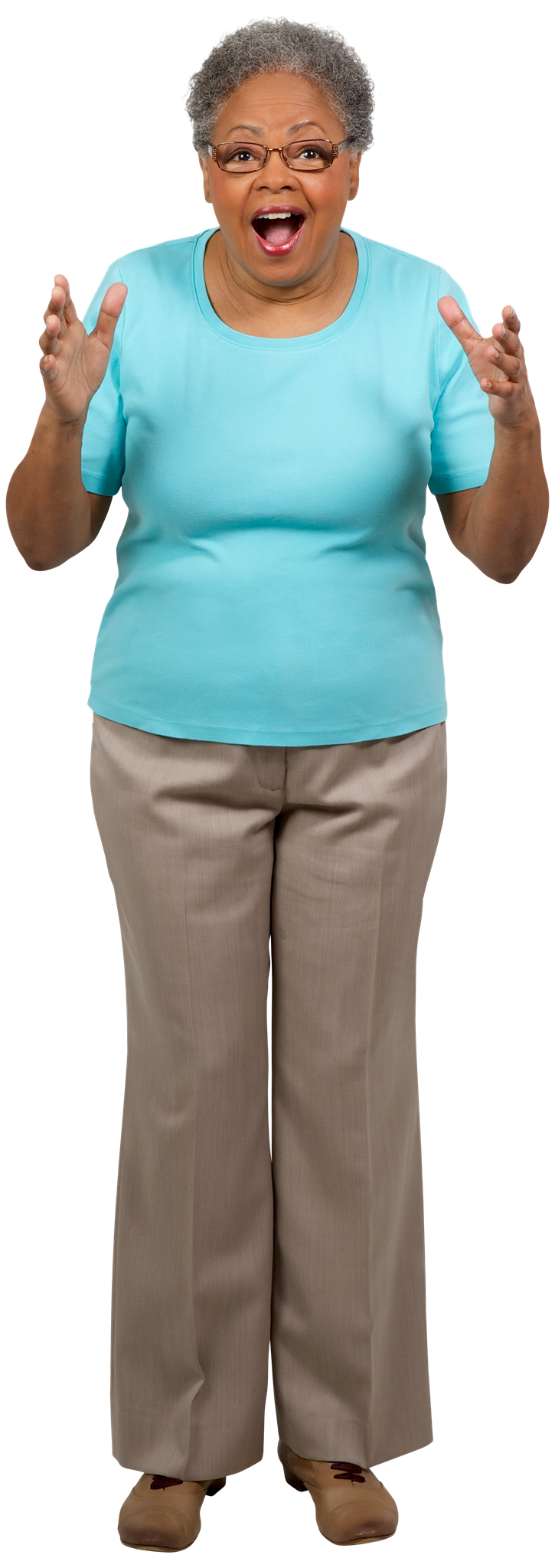

Supplement: Supplementary file 1 — Asthma Module folderTracheostomy Module folderChronic Cough Module folderObstructive Sleep Apnea Module folderPosttest Questions.docxFeedback.docx [file mep_2374-8265.11470-s001.zip › A. Asthma Module/scormcontent/assets/wX-eY7Gxc8Y3sbOE_171_full.png]

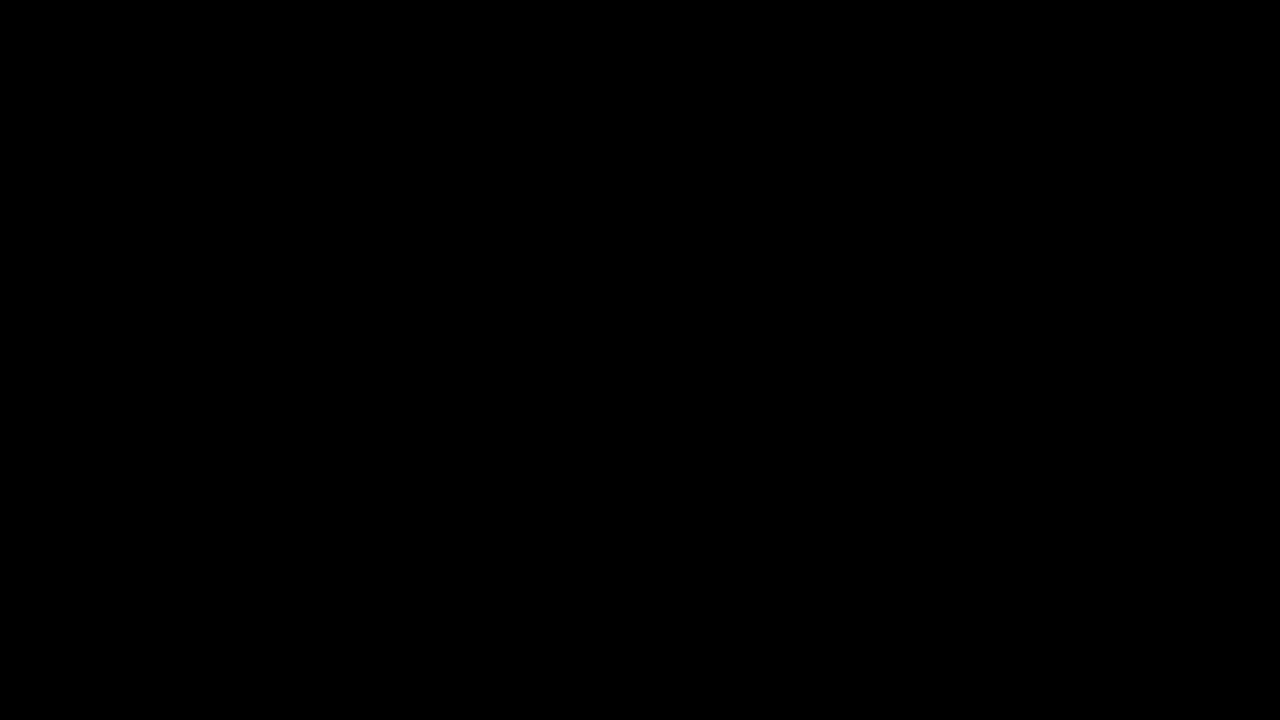

Supplement: Supplementary file 1 — Asthma Module folderTracheostomy Module folderChronic Cough Module folderObstructive Sleep Apnea Module folderPosttest Questions.docxFeedback.docx [file mep_2374-8265.11470-s001.zip › A. Asthma Module/scormcontent/assets/zoom_0(1).jpg]

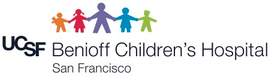

Supplement: Supplementary file 1 — Asthma Module folderTracheostomy Module folderChronic Cough Module folderObstructive Sleep Apnea Module folderPosttest Questions.docxFeedback.docx [file mep_2374-8265.11470-s001.zip › B. Tracheostomy Module/scormcontent/assets/77hO9jXor3JkA4FP.jpg]

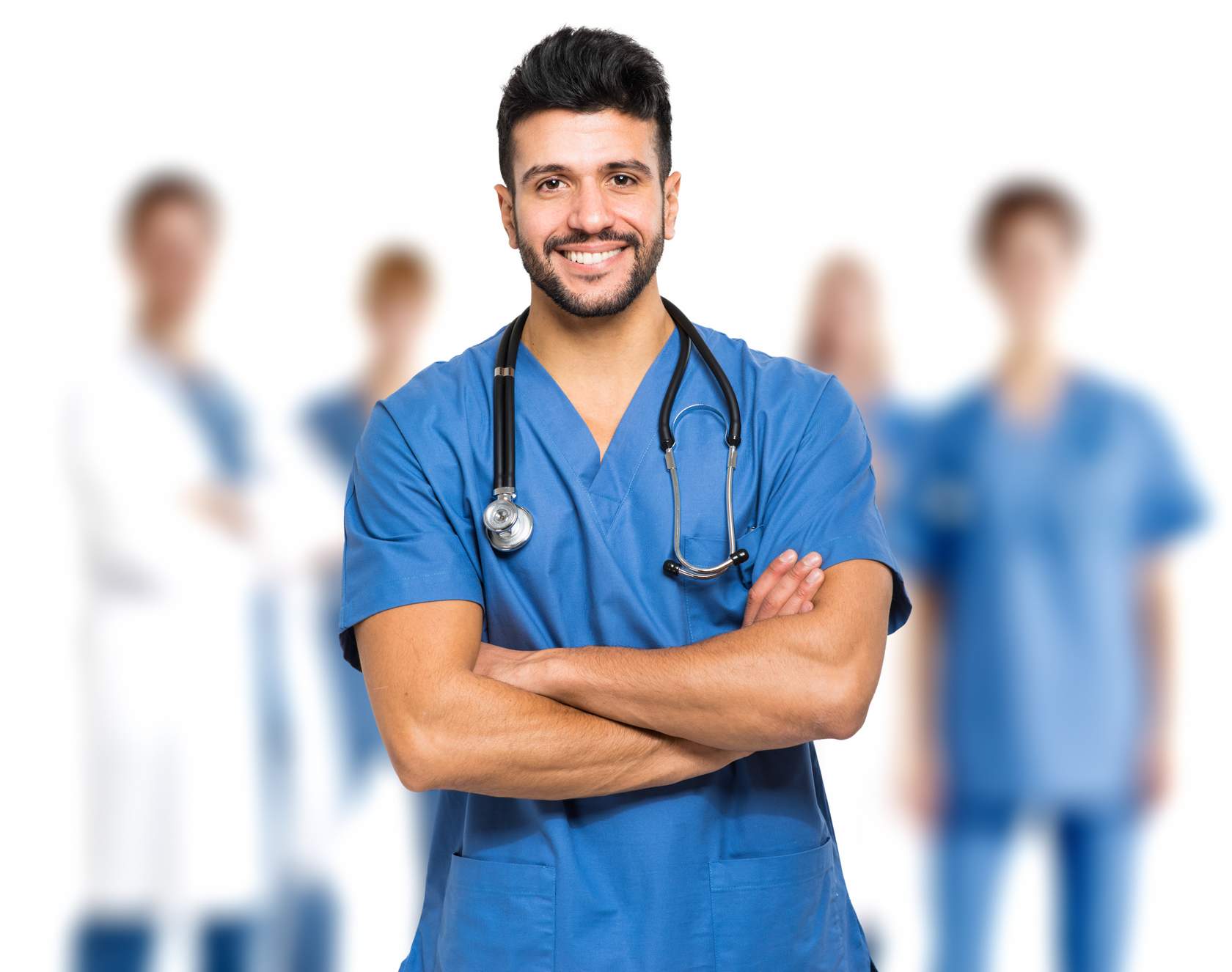

Supplement: Supplementary file 1 — Asthma Module folderTracheostomy Module folderChronic Cough Module folderObstructive Sleep Apnea Module folderPosttest Questions.docxFeedback.docx [file mep_2374-8265.11470-s001.zip › B. Tracheostomy Module/scormcontent/assets/iStock-590614440.jpg]

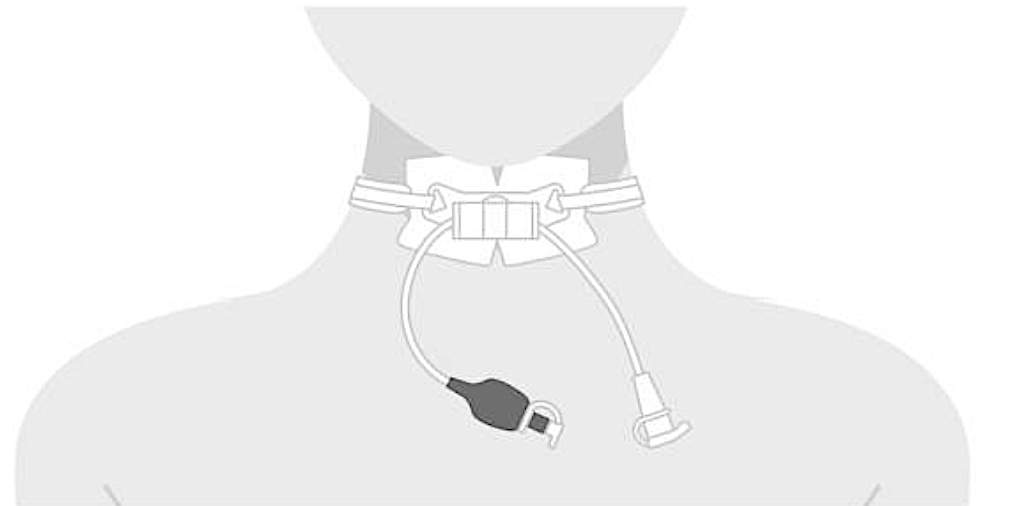

Supplement: Supplementary file 1 — Asthma Module folderTracheostomy Module folderChronic Cough Module folderObstructive Sleep Apnea Module folderPosttest Questions.docxFeedback.docx [file mep_2374-8265.11470-s001.zip › B. Tracheostomy Module/scormcontent/assets/jfEzY7AJIivS1pZY.jpg]

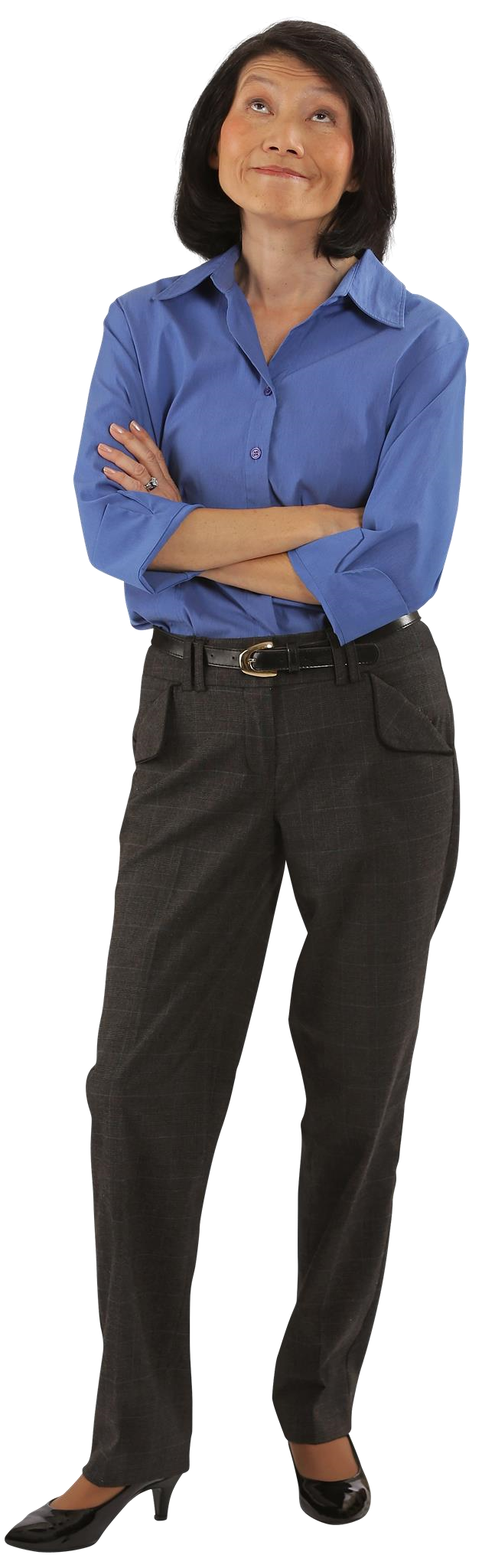

Supplement: Supplementary file 1 — Asthma Module folderTracheostomy Module folderChronic Cough Module folderObstructive Sleep Apnea Module folderPosttest Questions.docxFeedback.docx [file mep_2374-8265.11470-s001.zip › C. Chronic Cough Module/scormcontent/assets/0epwcAGL7SuVJmsj_279_full.png]

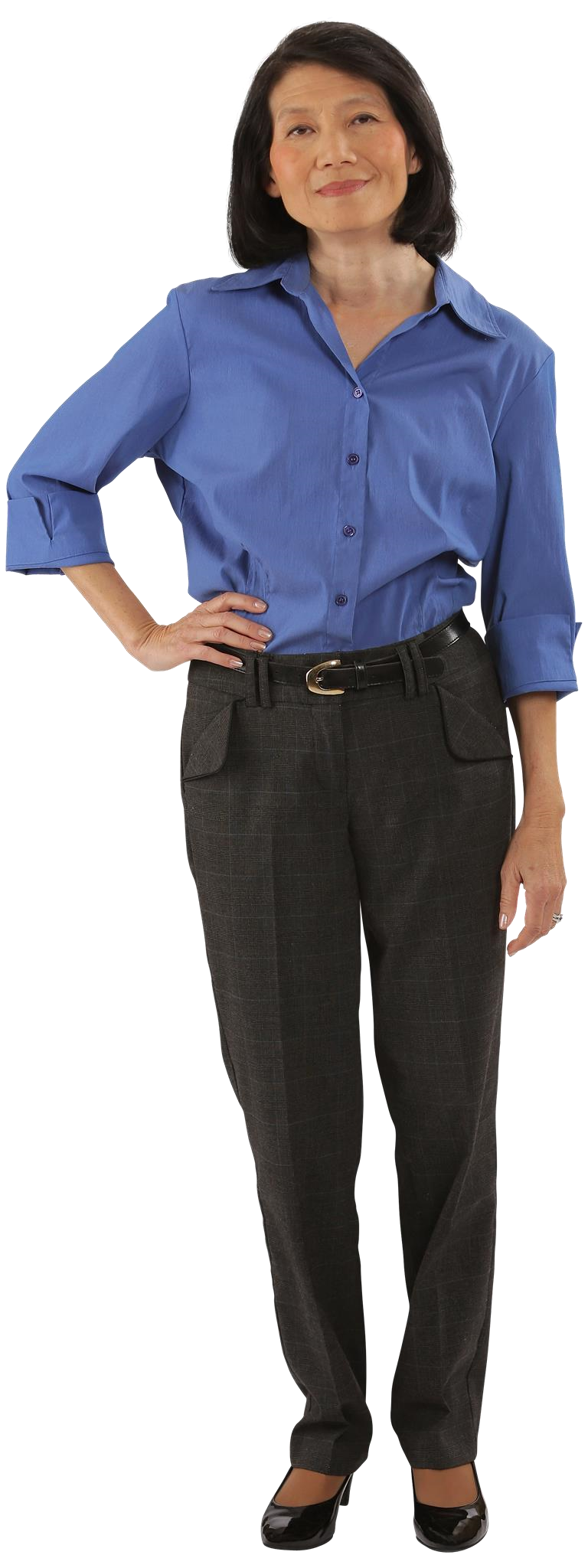

Supplement: Supplementary file 1 — Asthma Module folderTracheostomy Module folderChronic Cough Module folderObstructive Sleep Apnea Module folderPosttest Questions.docxFeedback.docx [file mep_2374-8265.11470-s001.zip › C. Chronic Cough Module/scormcontent/assets/0H7Q2MSe-sN83Aqj_202_full.png]

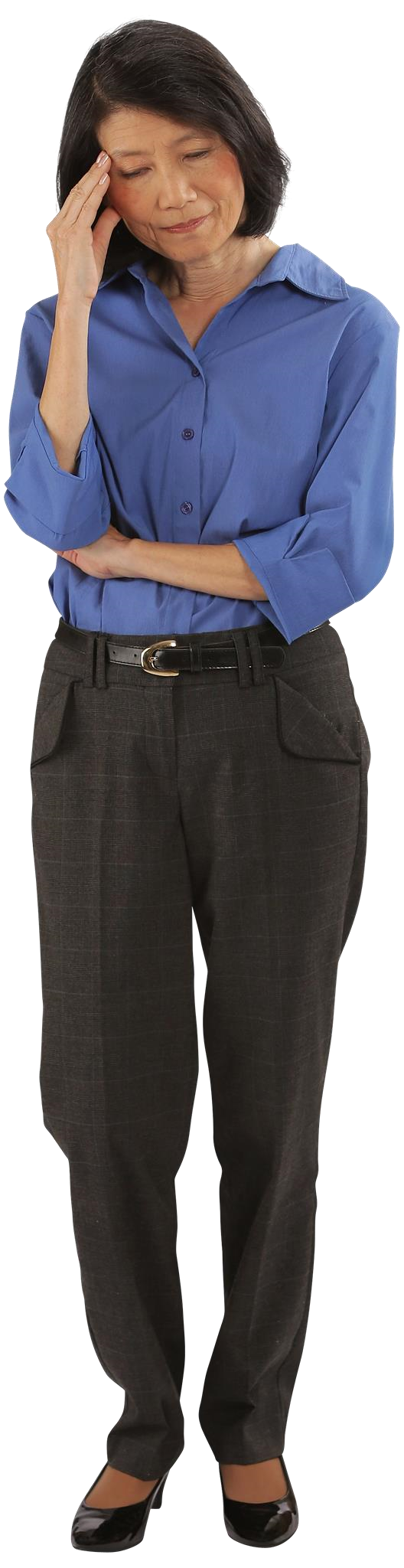

Supplement: Supplementary file 1 — Asthma Module folderTracheostomy Module folderChronic Cough Module folderObstructive Sleep Apnea Module folderPosttest Questions.docxFeedback.docx [file mep_2374-8265.11470-s001.zip › C. Chronic Cough Module/scormcontent/assets/C1l-cKauPYpz3QF0_310_full.png]

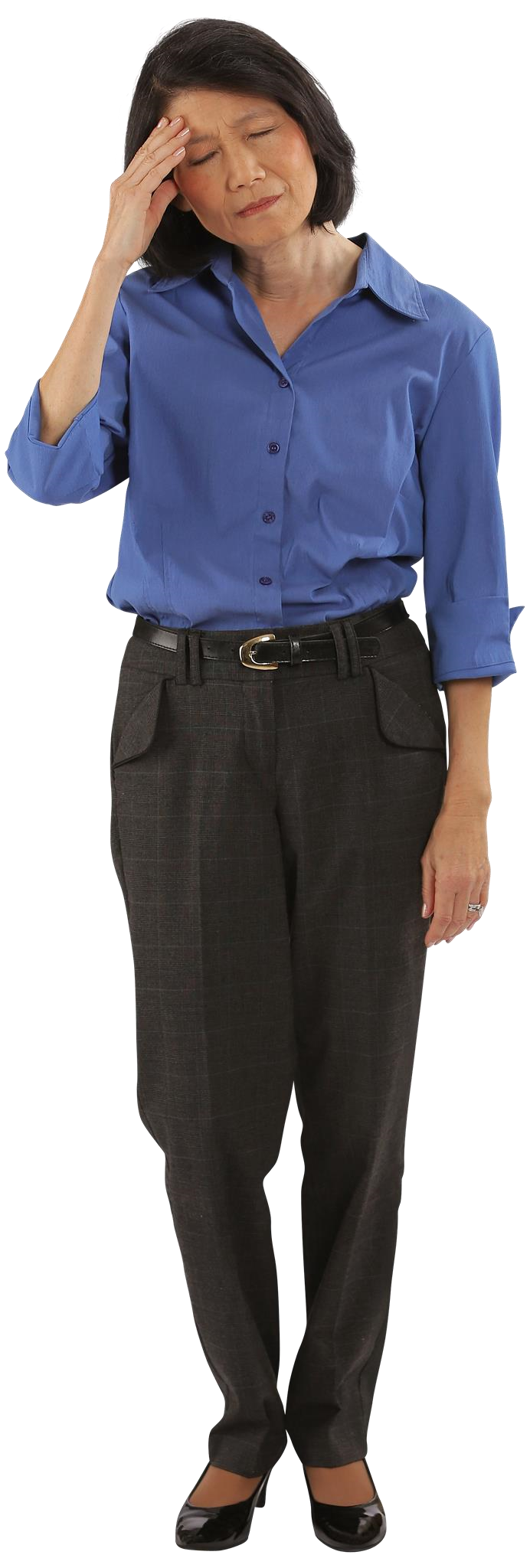

Supplement: Supplementary file 1 — Asthma Module folderTracheostomy Module folderChronic Cough Module folderObstructive Sleep Apnea Module folderPosttest Questions.docxFeedback.docx [file mep_2374-8265.11470-s001.zip › C. Chronic Cough Module/scormcontent/assets/c3GO3elFU8pcPIeQ_281_full.png]

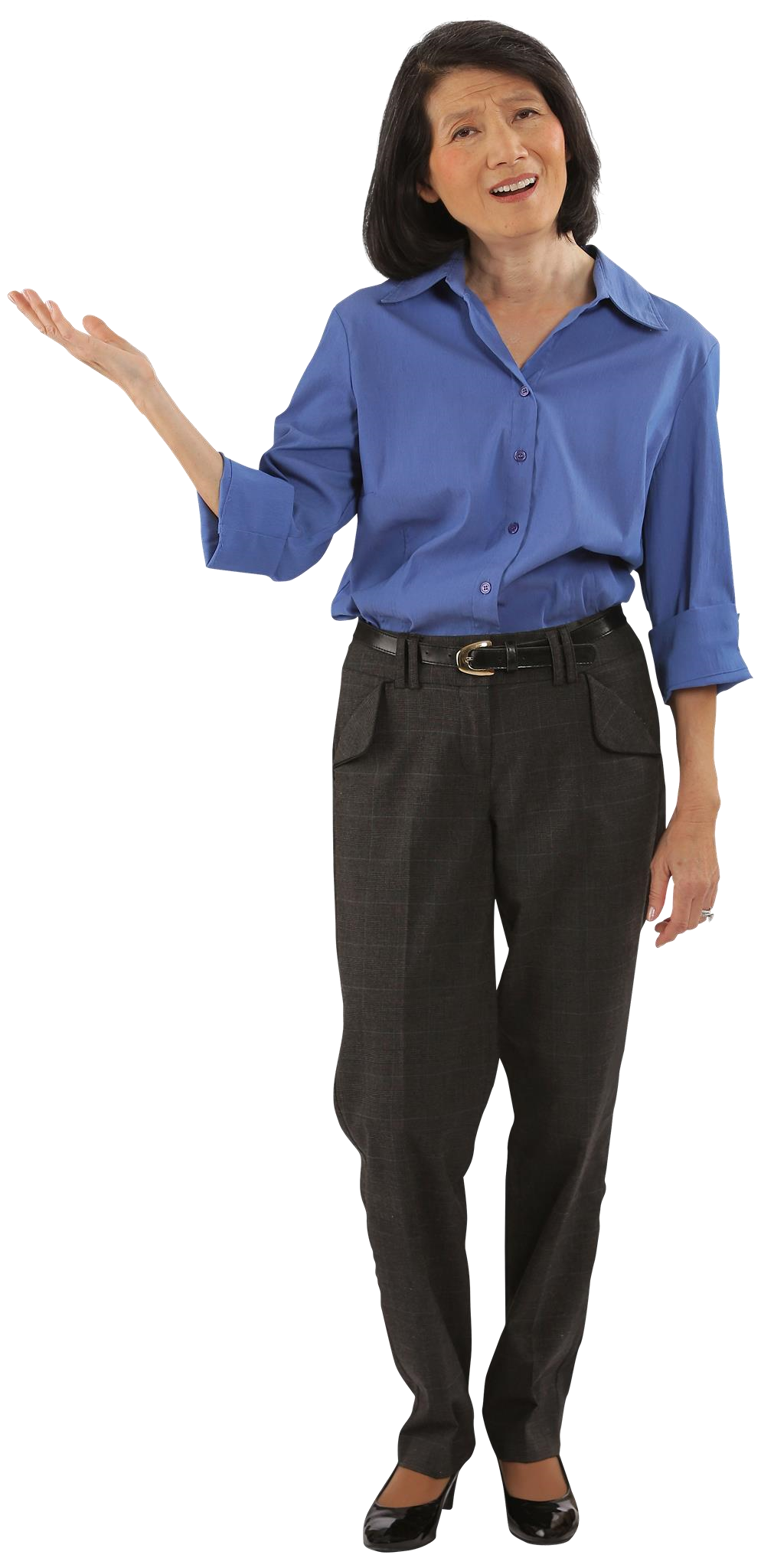

Supplement: Supplementary file 1 — Asthma Module folderTracheostomy Module folderChronic Cough Module folderObstructive Sleep Apnea Module folderPosttest Questions.docxFeedback.docx [file mep_2374-8265.11470-s001.zip › C. Chronic Cough Module/scormcontent/assets/EhvjZHWgBkRBQl8d_274_full.png]

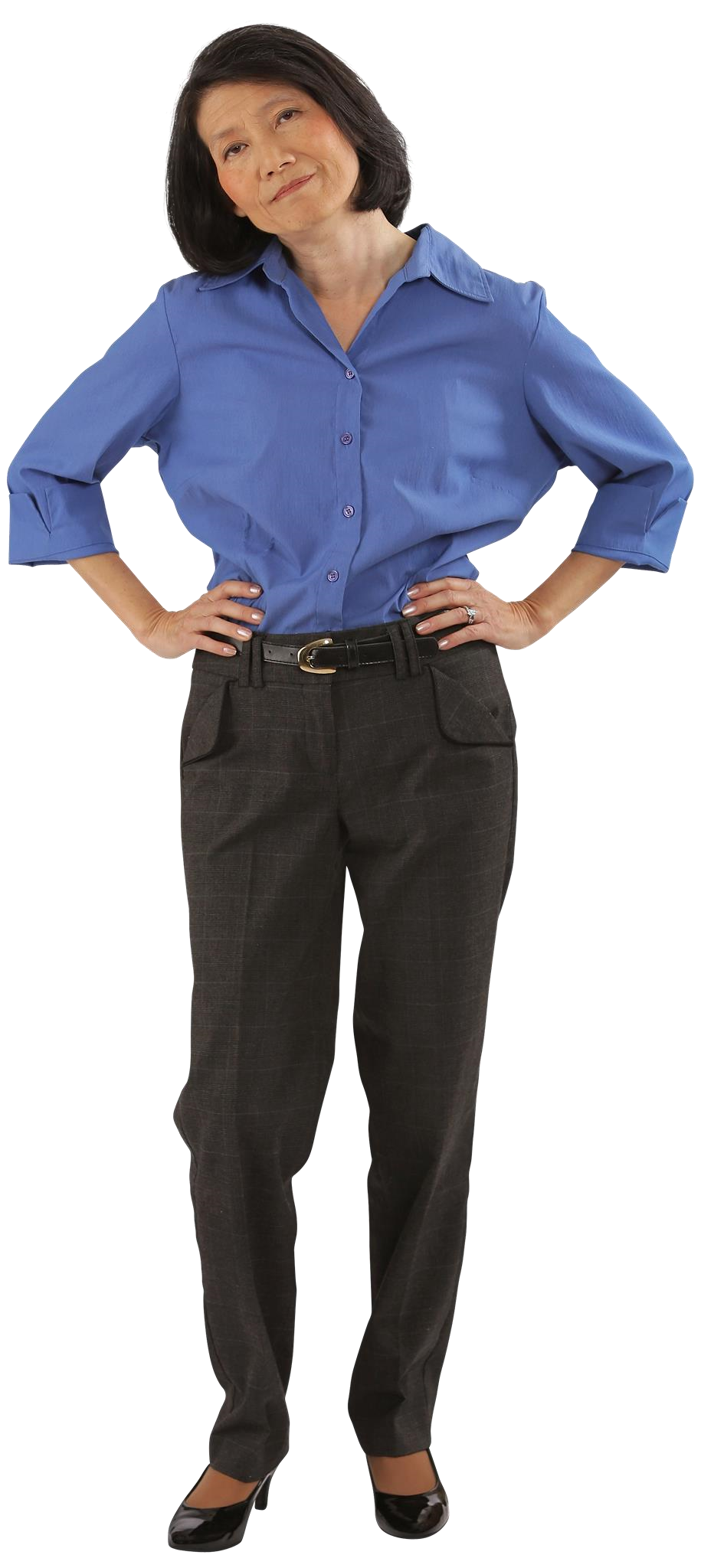

Supplement: Supplementary file 1 — Asthma Module folderTracheostomy Module folderChronic Cough Module folderObstructive Sleep Apnea Module folderPosttest Questions.docxFeedback.docx [file mep_2374-8265.11470-s001.zip › C. Chronic Cough Module/scormcontent/assets/fVhg1DS1VdF98vO1_303_full.png]

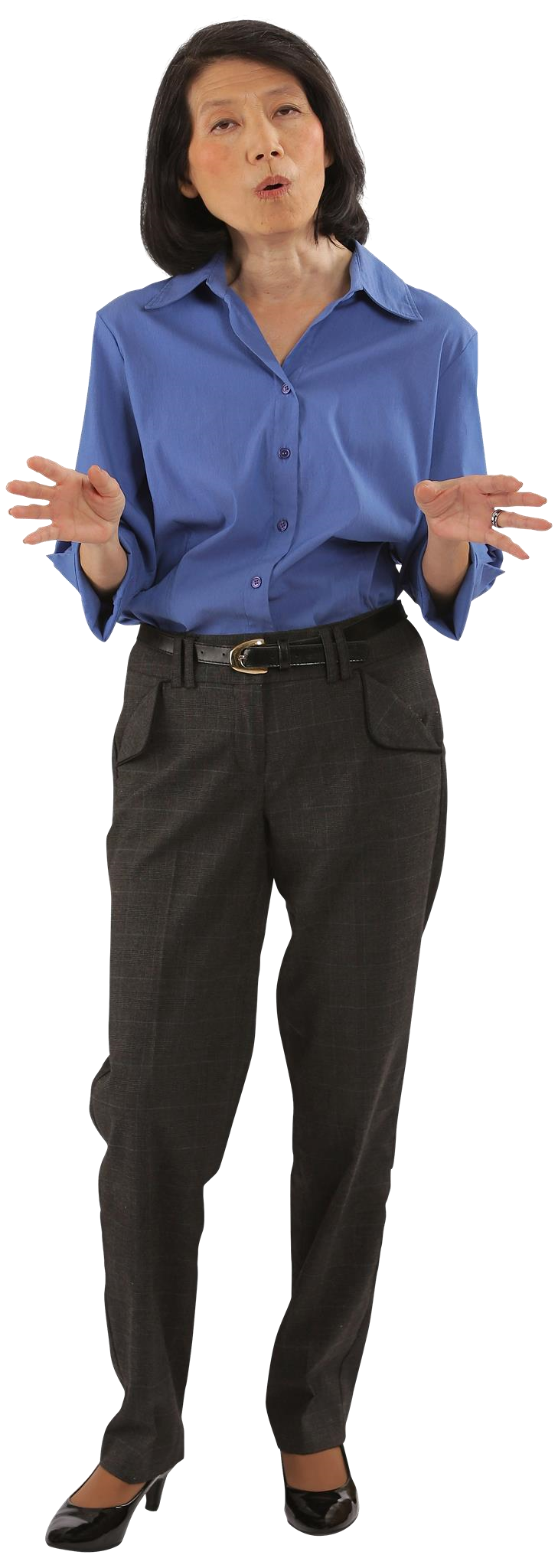

Supplement: Supplementary file 1 — Asthma Module folderTracheostomy Module folderChronic Cough Module folderObstructive Sleep Apnea Module folderPosttest Questions.docxFeedback.docx [file mep_2374-8265.11470-s001.zip › C. Chronic Cough Module/scormcontent/assets/FzrmB1jI3j0bo-hd_264_full.png]

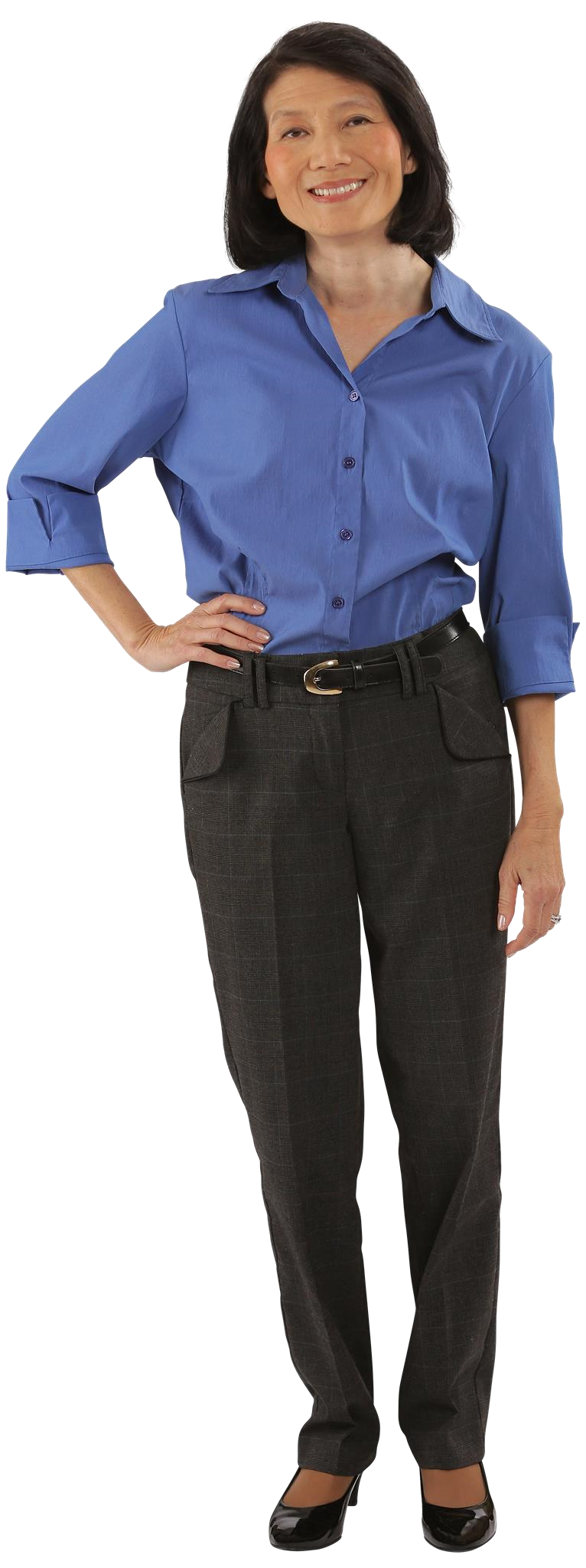

Supplement: Supplementary file 1 — Asthma Module folderTracheostomy Module folderChronic Cough Module folderObstructive Sleep Apnea Module folderPosttest Questions.docxFeedback.docx [file mep_2374-8265.11470-s001.zip › C. Chronic Cough Module/scormcontent/assets/GTQji5XGdrLcuE08_203_full.png]

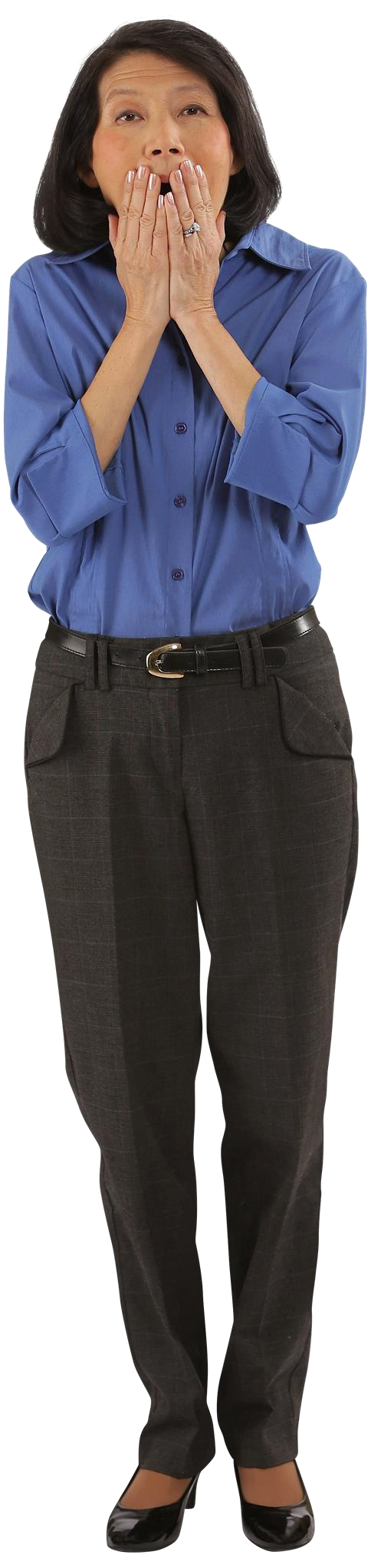

Supplement: Supplementary file 1 — Asthma Module folderTracheostomy Module folderChronic Cough Module folderObstructive Sleep Apnea Module folderPosttest Questions.docxFeedback.docx [file mep_2374-8265.11470-s001.zip › C. Chronic Cough Module/scormcontent/assets/ij6XXpxjaSzTCHSQ_284_full.png]

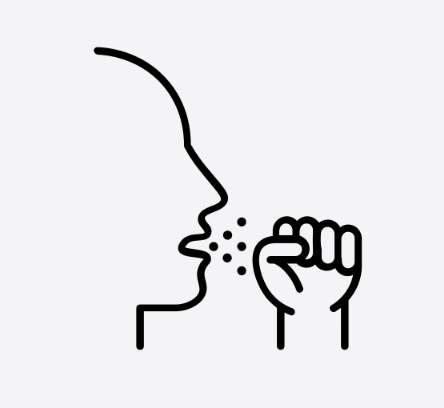

Supplement: Supplementary file 1 — Asthma Module folderTracheostomy Module folderChronic Cough Module folderObstructive Sleep Apnea Module folderPosttest Questions.docxFeedback.docx [file mep_2374-8265.11470-s001.zip › C. Chronic Cough Module/scormcontent/assets/IKv_y9Muhq1-J2o3.jpg]

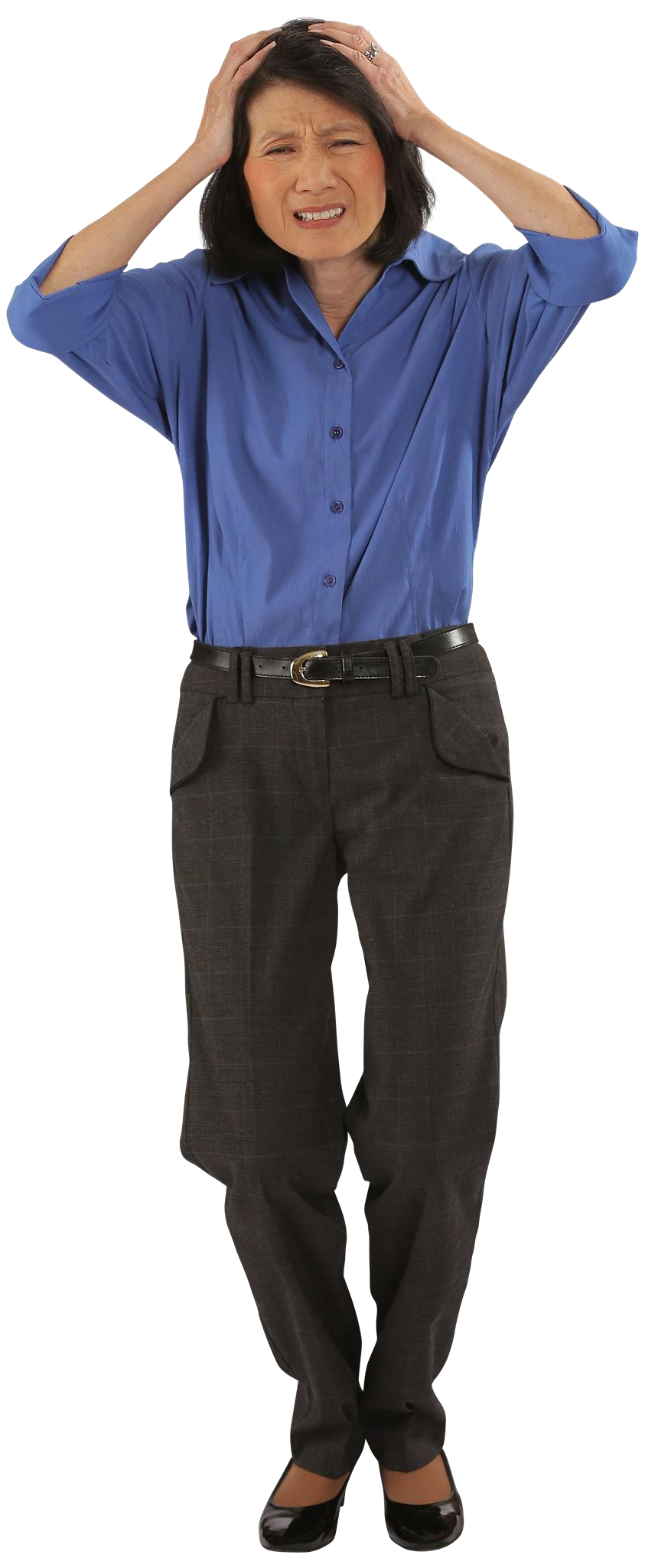

Supplement: Supplementary file 1 — Asthma Module folderTracheostomy Module folderChronic Cough Module folderObstructive Sleep Apnea Module folderPosttest Questions.docxFeedback.docx [file mep_2374-8265.11470-s001.zip › C. Chronic Cough Module/scormcontent/assets/joIblU8A-TZWQg_h_306_full.png]

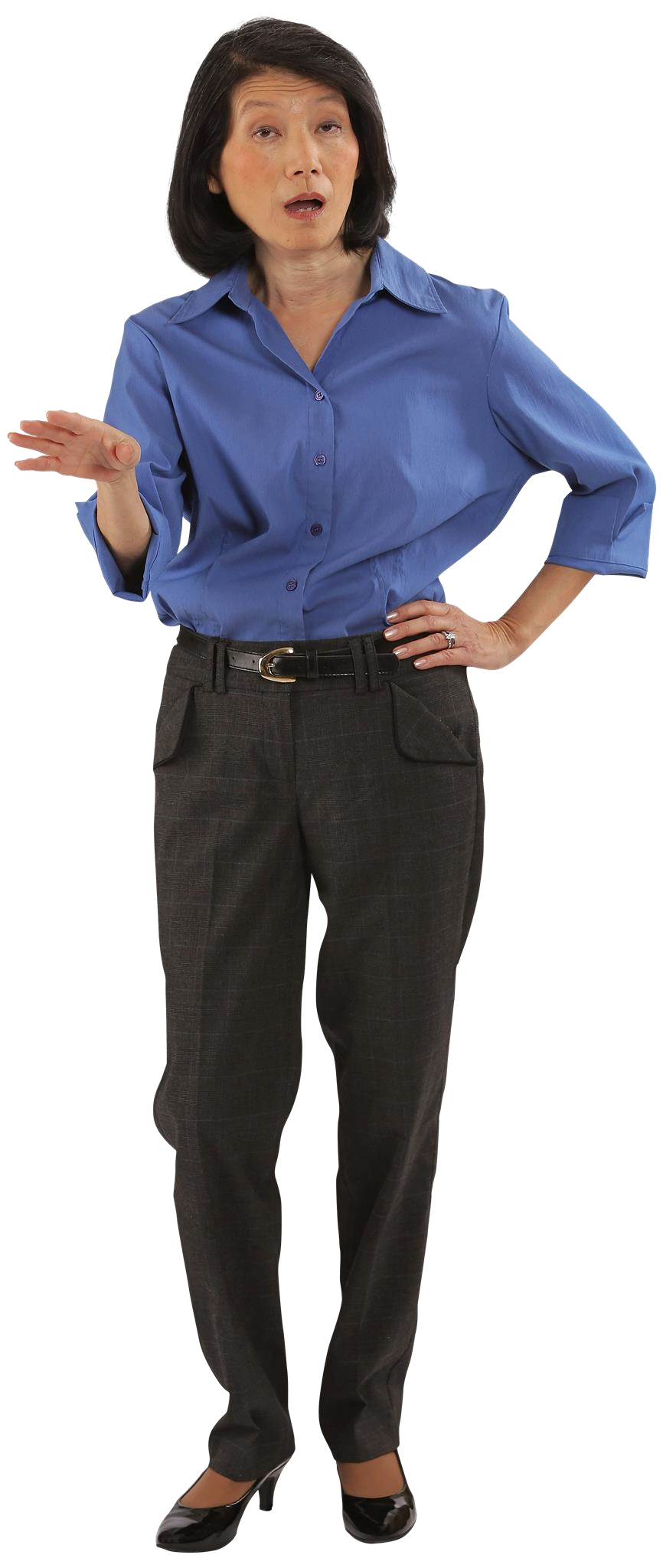

Supplement: Supplementary file 1 — Asthma Module folderTracheostomy Module folderChronic Cough Module folderObstructive Sleep Apnea Module folderPosttest Questions.docxFeedback.docx [file mep_2374-8265.11470-s001.zip › C. Chronic Cough Module/scormcontent/assets/L8VBq_9Lo2E-33tm_258_full.png]

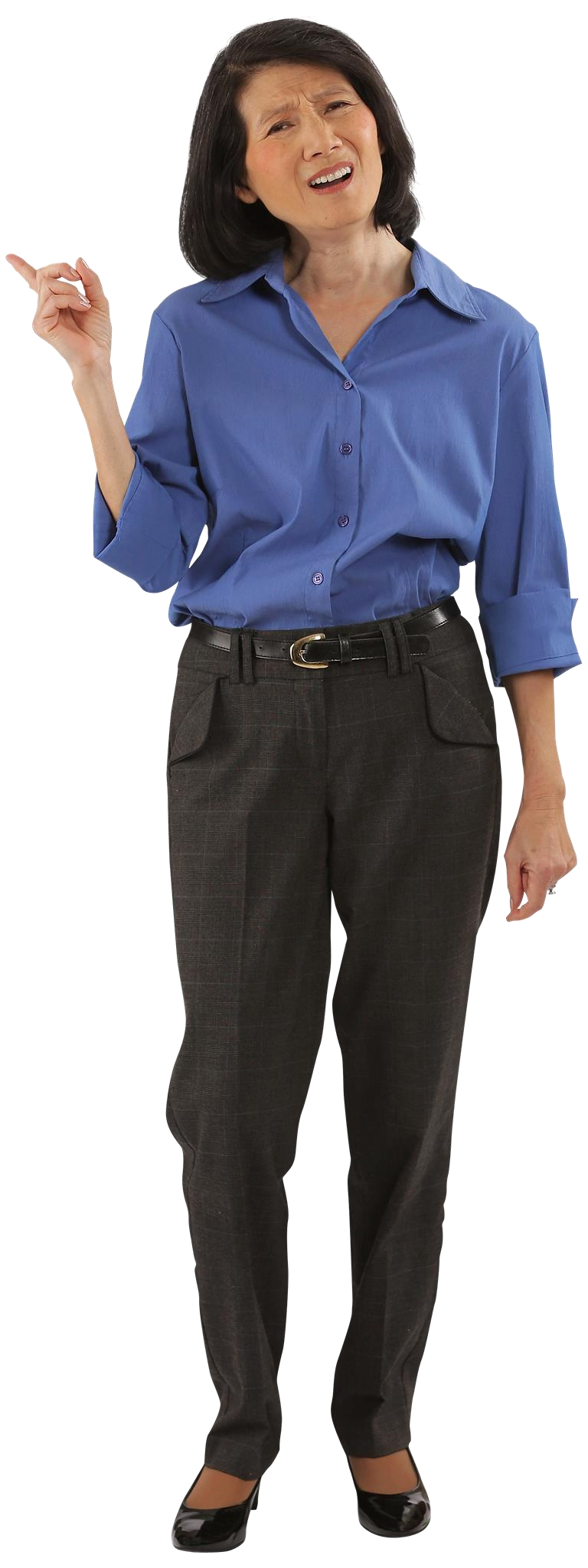

Supplement: Supplementary file 1 — Asthma Module folderTracheostomy Module folderChronic Cough Module folderObstructive Sleep Apnea Module folderPosttest Questions.docxFeedback.docx [file mep_2374-8265.11470-s001.zip › C. Chronic Cough Module/scormcontent/assets/M0BEohVVFpExzZ_5_267_full.png]

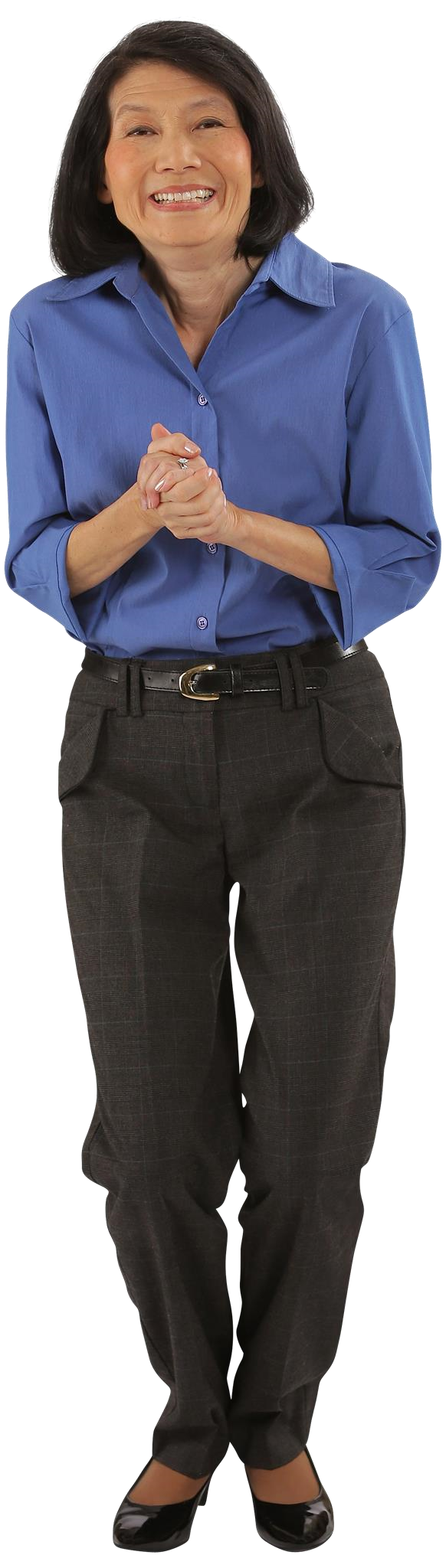

Supplement: Supplementary file 1 — Asthma Module folderTracheostomy Module folderChronic Cough Module folderObstructive Sleep Apnea Module folderPosttest Questions.docxFeedback.docx [file mep_2374-8265.11470-s001.zip › C. Chronic Cough Module/scormcontent/assets/Mks5Mpe4-Esp1pbQ_282_full.png]

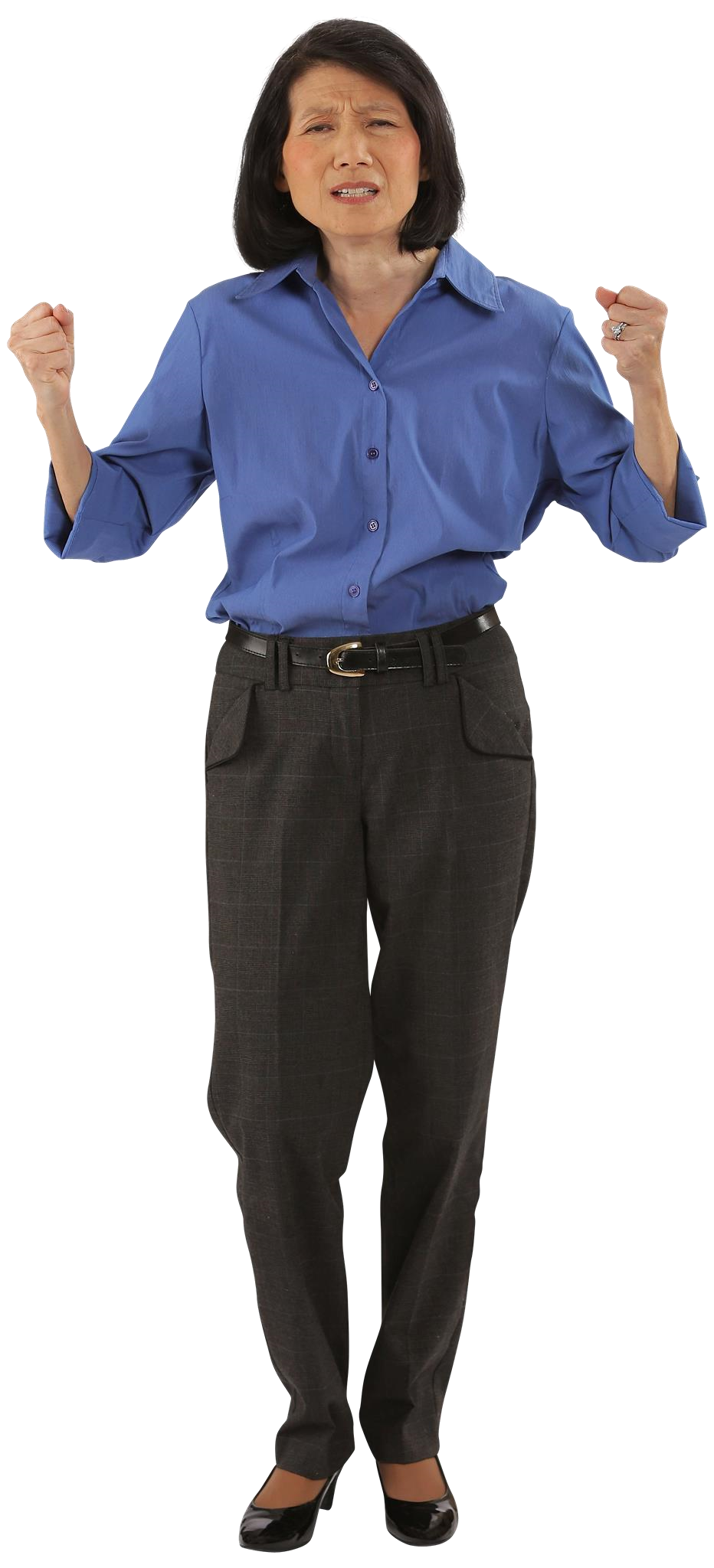

Supplement: Supplementary file 1 — Asthma Module folderTracheostomy Module folderChronic Cough Module folderObstructive Sleep Apnea Module folderPosttest Questions.docxFeedback.docx [file mep_2374-8265.11470-s001.zip › C. Chronic Cough Module/scormcontent/assets/OVBnGLRXG_E3nsEO_277_full.png]

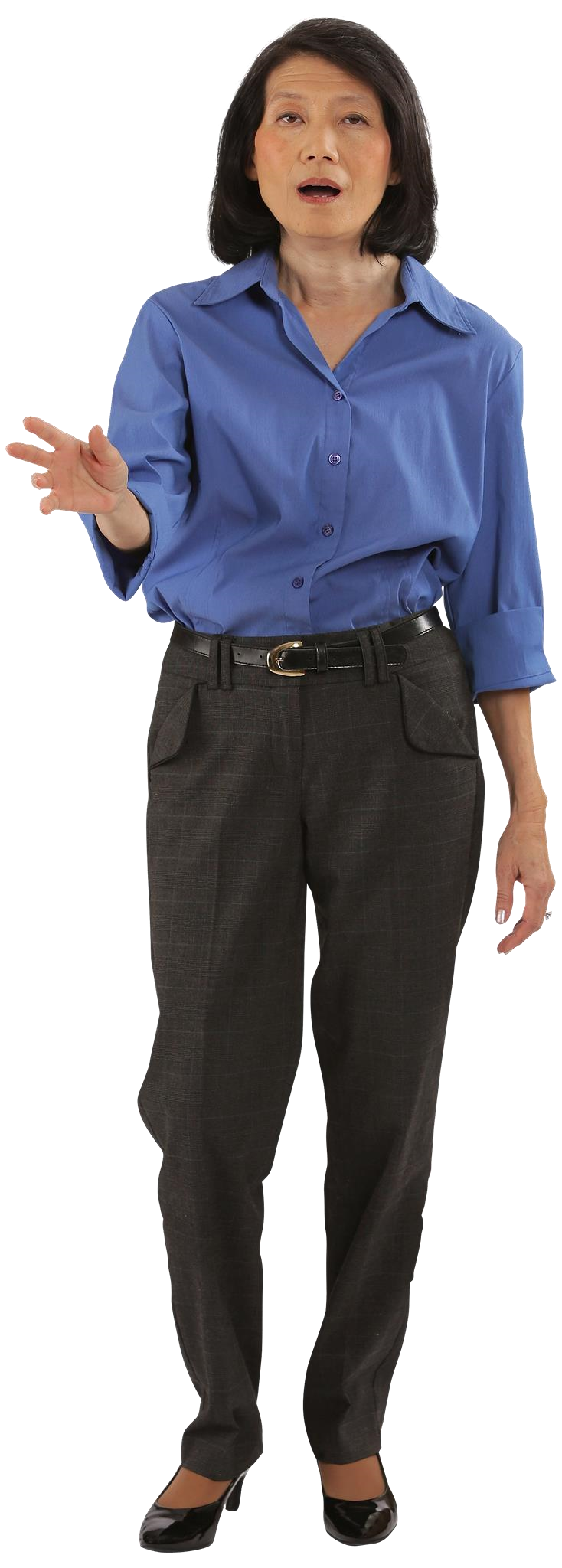

Supplement: Supplementary file 1 — Asthma Module folderTracheostomy Module folderChronic Cough Module folderObstructive Sleep Apnea Module folderPosttest Questions.docxFeedback.docx [file mep_2374-8265.11470-s001.zip › C. Chronic Cough Module/scormcontent/assets/QGhqLI05iETKbVGV_261_full.png]

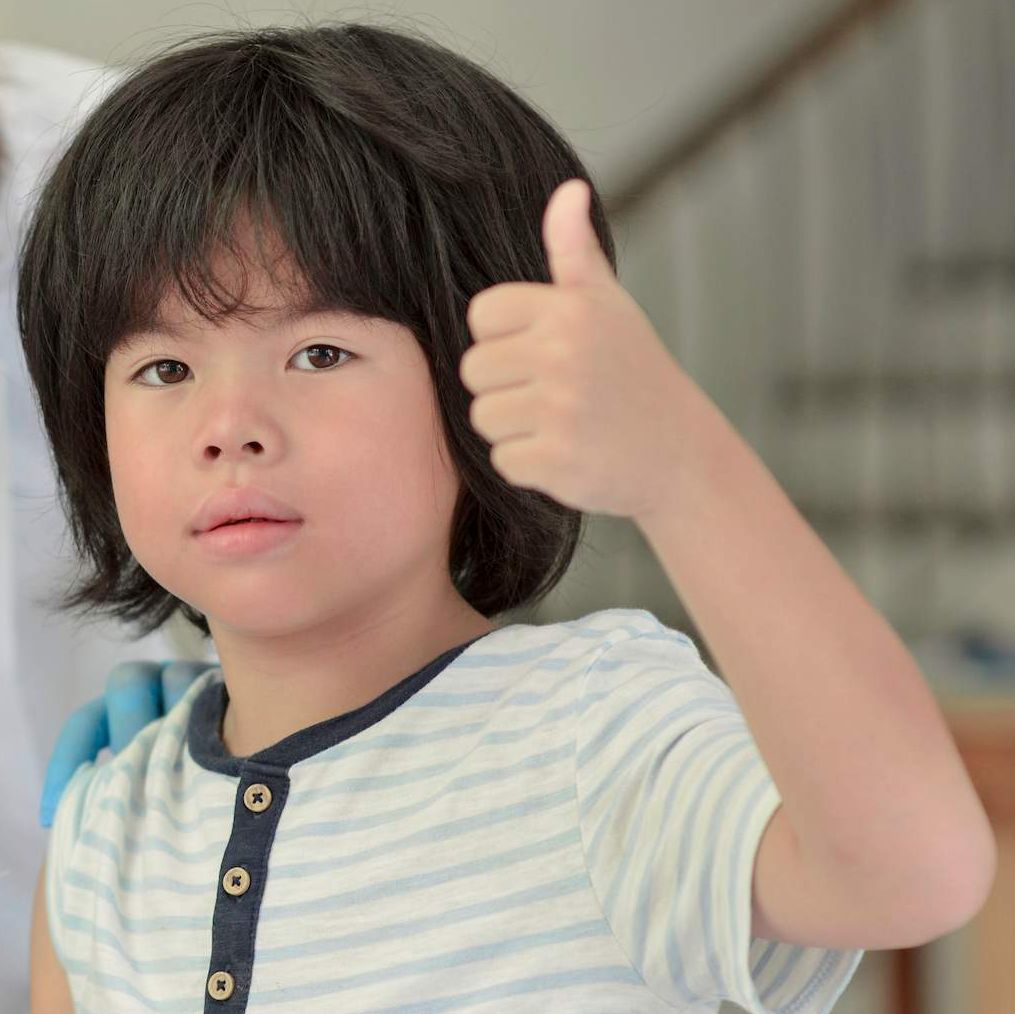

Supplement: Supplementary file 1 — Asthma Module folderTracheostomy Module folderChronic Cough Module folderObstructive Sleep Apnea Module folderPosttest Questions.docxFeedback.docx [file mep_2374-8265.11470-s001.zip › C. Chronic Cough Module/scormcontent/assets/Screen Shot 2021-09-29 at 10.18.15 PM.jpg]

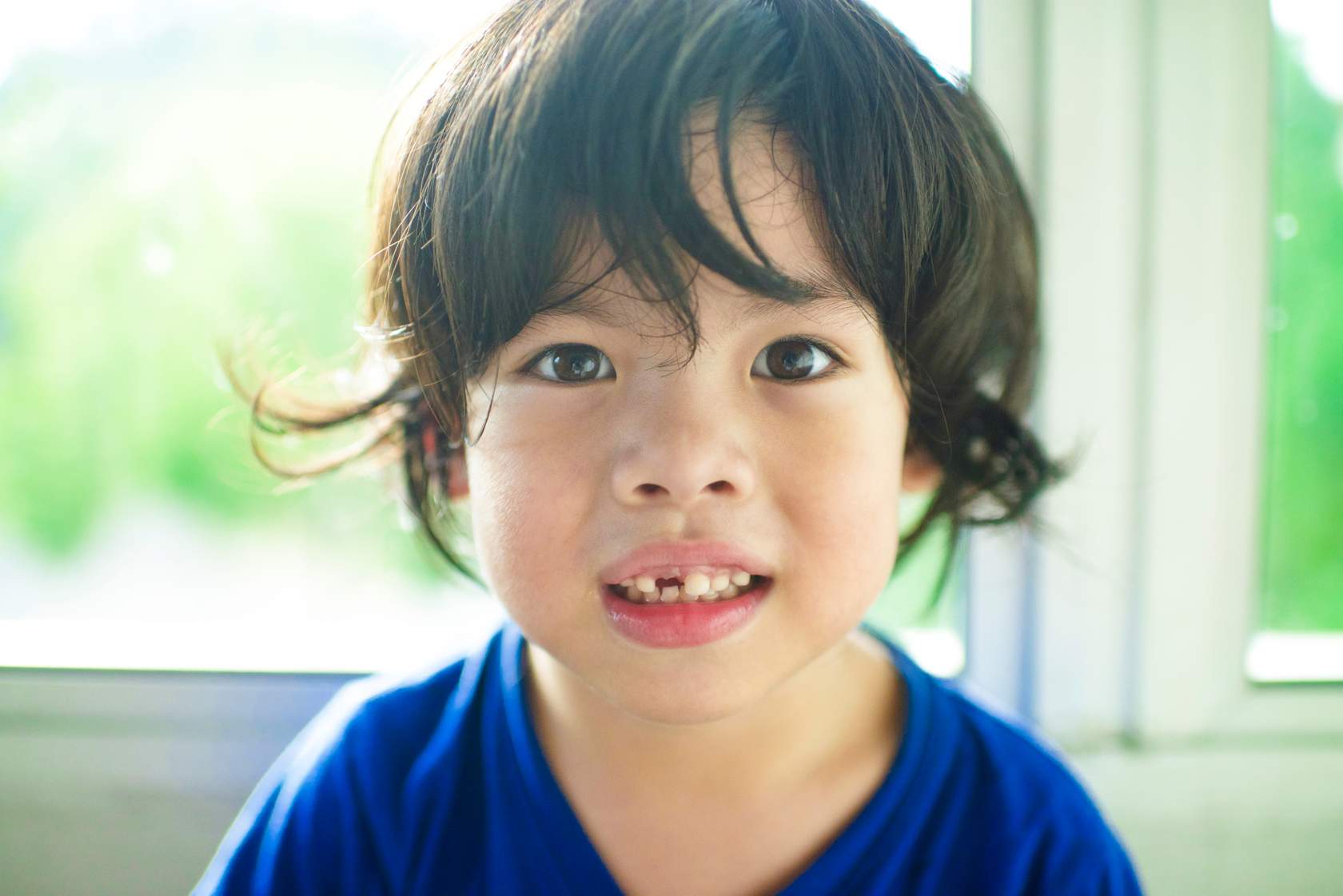

Supplement: Supplementary file 1 — Asthma Module folderTracheostomy Module folderChronic Cough Module folderObstructive Sleep Apnea Module folderPosttest Questions.docxFeedback.docx [file mep_2374-8265.11470-s001.zip › C. Chronic Cough Module/scormcontent/assets/shutterstock_1636863793.jpg]

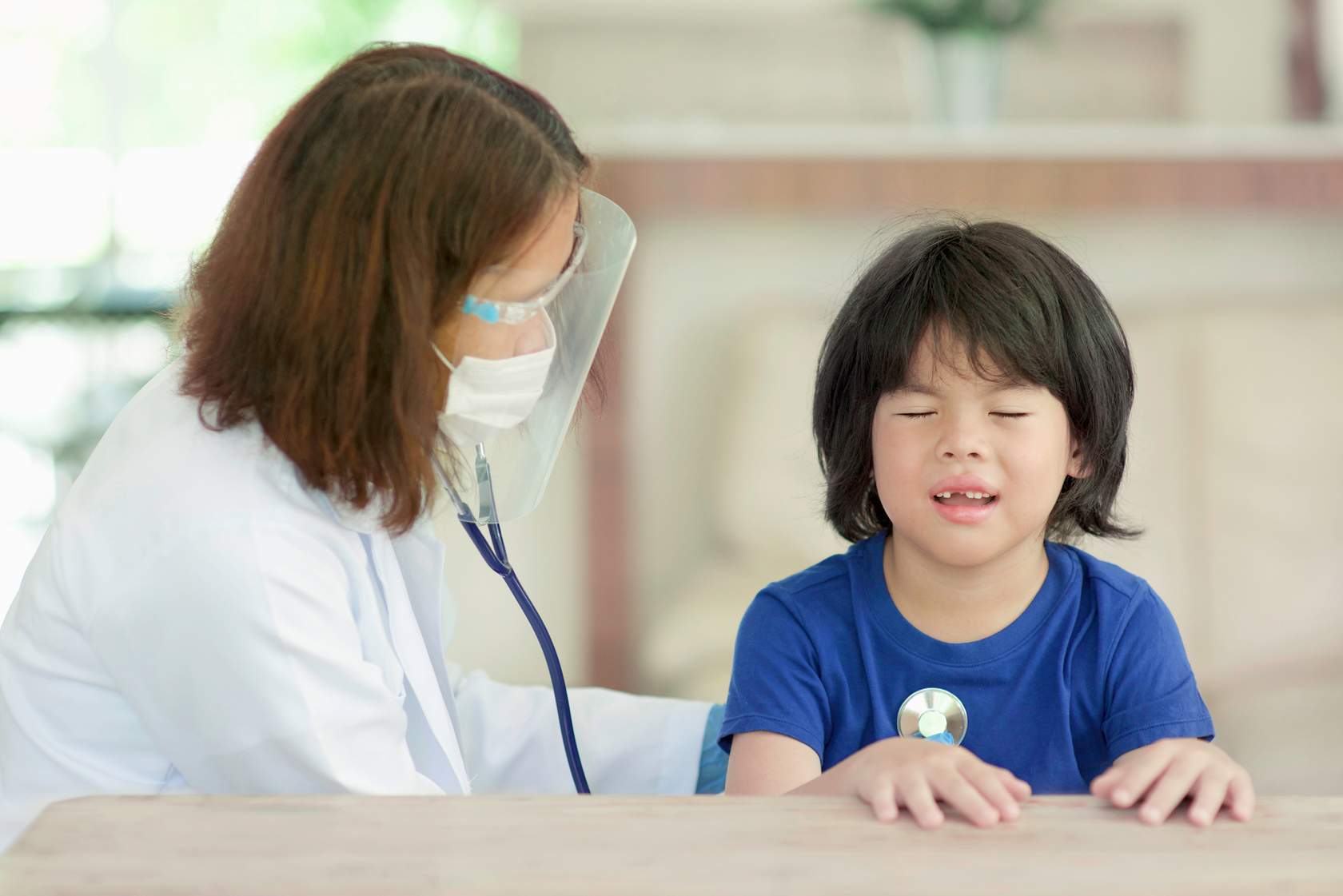

Supplement: Supplementary file 1 — Asthma Module folderTracheostomy Module folderChronic Cough Module folderObstructive Sleep Apnea Module folderPosttest Questions.docxFeedback.docx [file mep_2374-8265.11470-s001.zip › C. Chronic Cough Module/scormcontent/assets/shutterstock_2030708006.jpg]

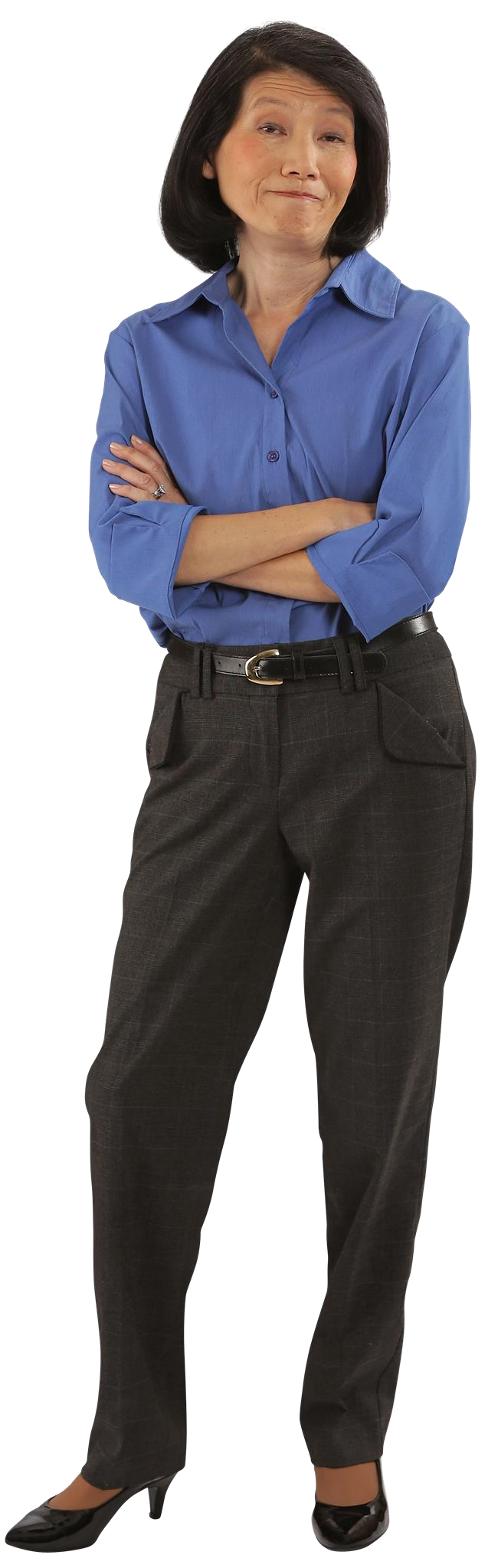

Supplement: Supplementary file 1 — Asthma Module folderTracheostomy Module folderChronic Cough Module folderObstructive Sleep Apnea Module folderPosttest Questions.docxFeedback.docx [file mep_2374-8265.11470-s001.zip › C. Chronic Cough Module/scormcontent/assets/tKDtThg4E14KkwOX_288_full.png]

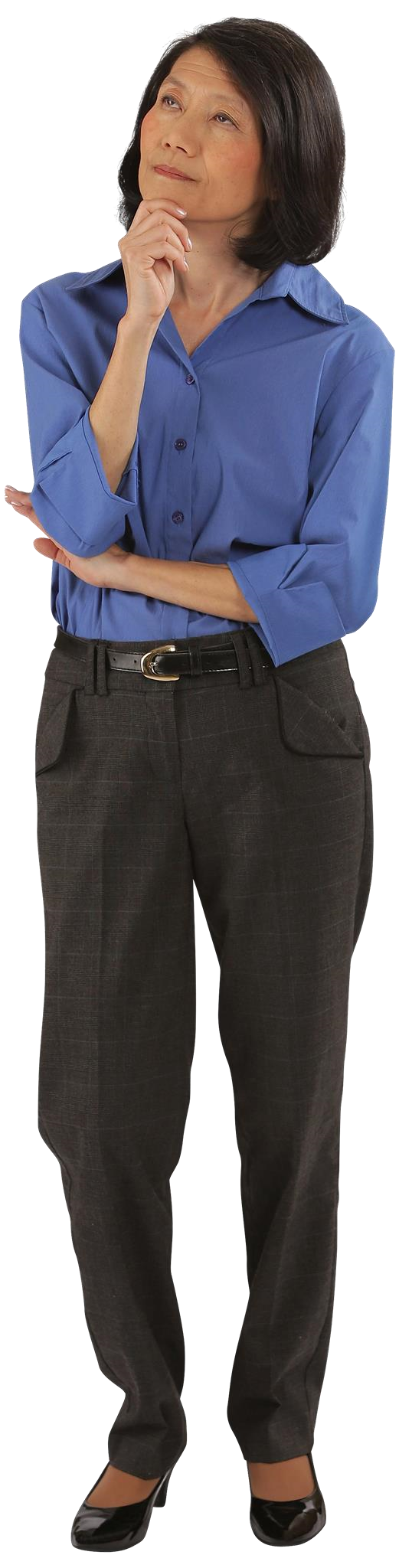

Supplement: Supplementary file 1 — Asthma Module folderTracheostomy Module folderChronic Cough Module folderObstructive Sleep Apnea Module folderPosttest Questions.docxFeedback.docx [file mep_2374-8265.11470-s001.zip › C. Chronic Cough Module/scormcontent/assets/vbOwwx9RuMfWS5e__312_full.png]

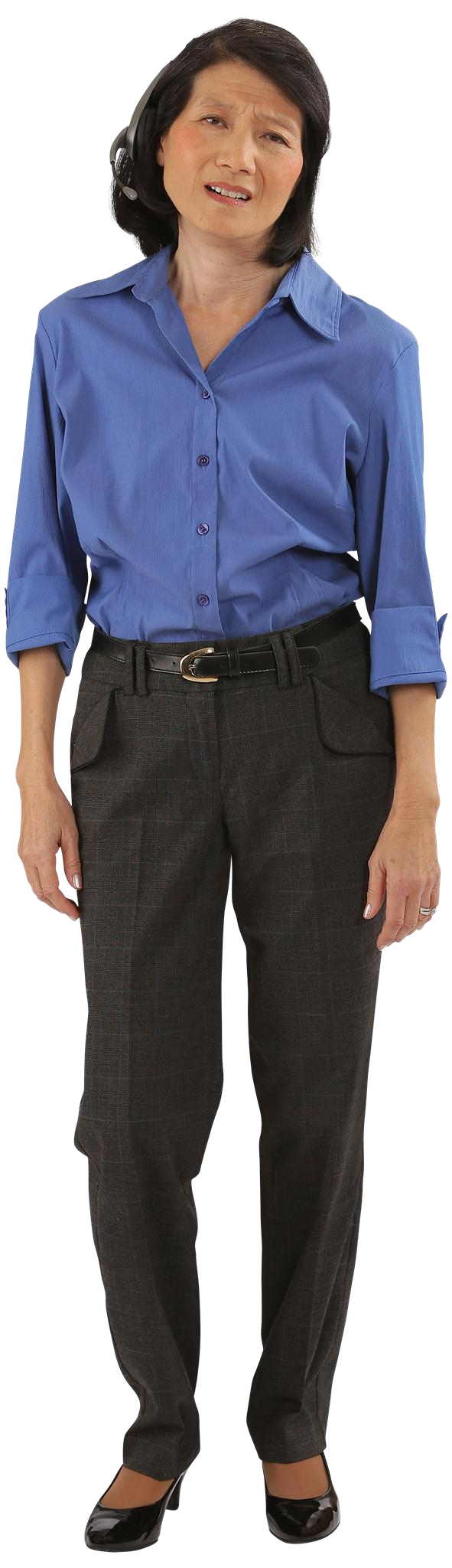

Supplement: Supplementary file 1 — Asthma Module folderTracheostomy Module folderChronic Cough Module folderObstructive Sleep Apnea Module folderPosttest Questions.docxFeedback.docx [file mep_2374-8265.11470-s001.zip › C. Chronic Cough Module/scormcontent/assets/zsX--yzfhae08_iA_192_full.png]

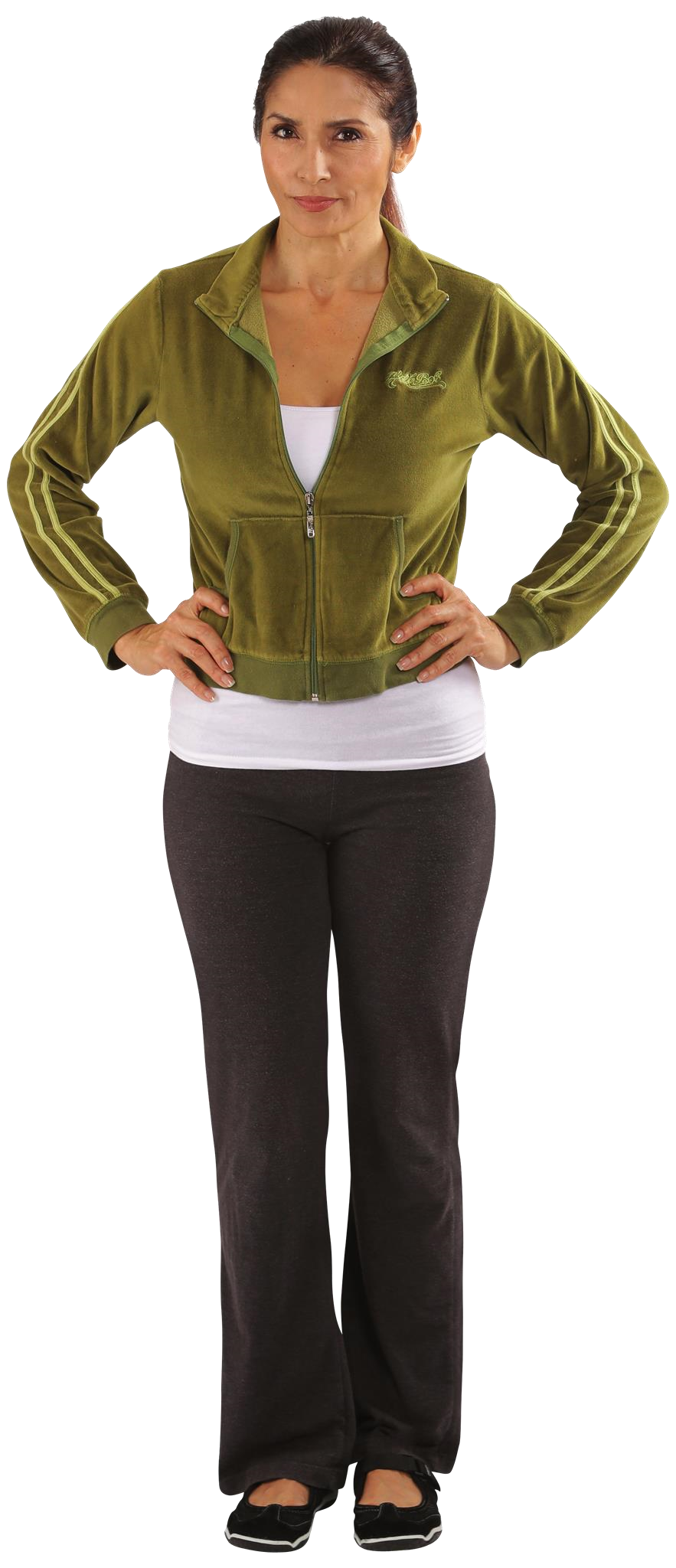

Supplement: Supplementary file 1 — Asthma Module folderTracheostomy Module folderChronic Cough Module folderObstructive Sleep Apnea Module folderPosttest Questions.docxFeedback.docx [file mep_2374-8265.11470-s001.zip › D. Obstructive Sleep Apnea Module/scormcontent/assets/3rSqB8kPYhFQizdZ_135_full.png]

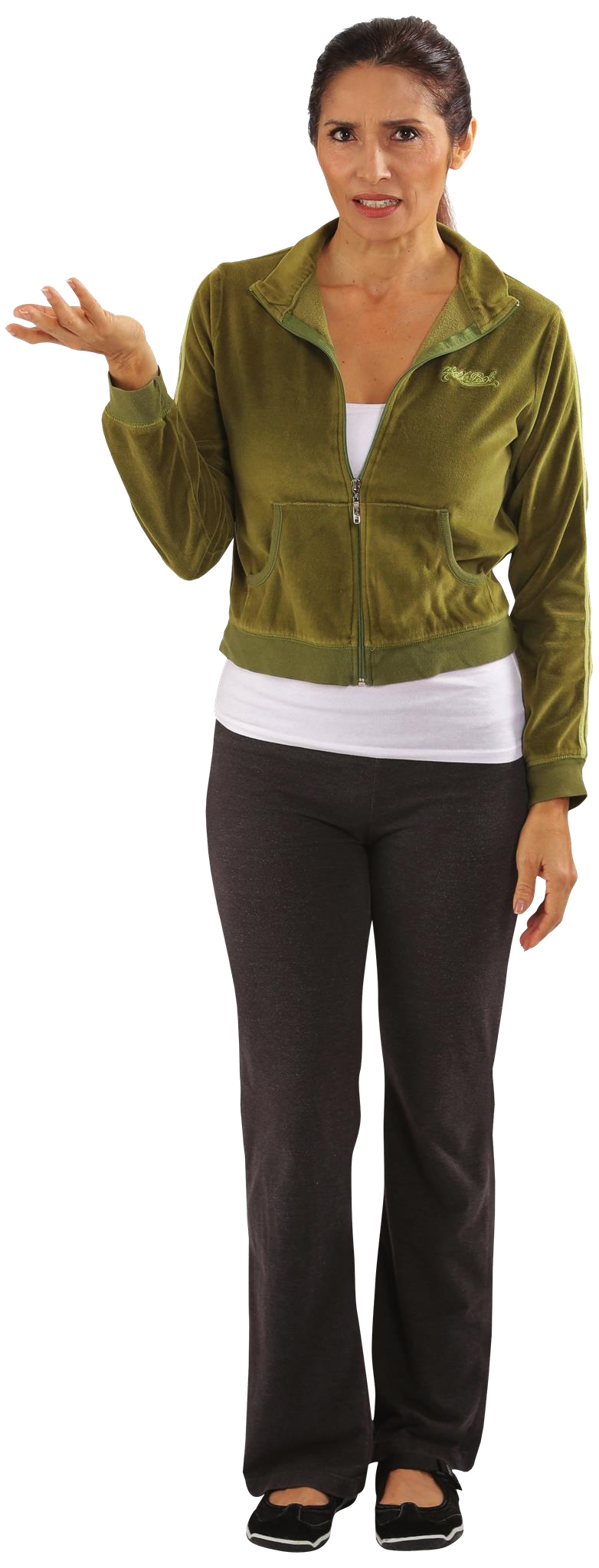

Supplement: Supplementary file 1 — Asthma Module folderTracheostomy Module folderChronic Cough Module folderObstructive Sleep Apnea Module folderPosttest Questions.docxFeedback.docx [file mep_2374-8265.11470-s001.zip › D. Obstructive Sleep Apnea Module/scormcontent/assets/5CjVzhtXlHwl3RpV_133_full.png]

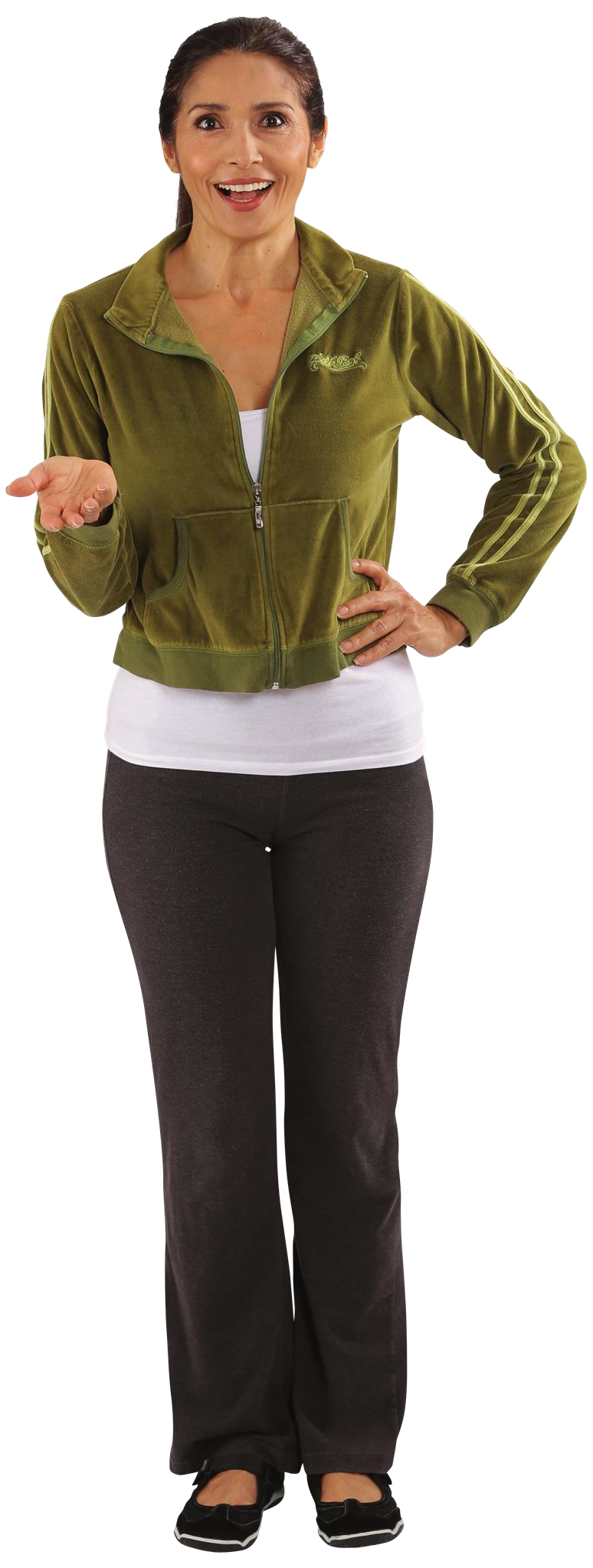

Supplement: Supplementary file 1 — Asthma Module folderTracheostomy Module folderChronic Cough Module folderObstructive Sleep Apnea Module folderPosttest Questions.docxFeedback.docx [file mep_2374-8265.11470-s001.zip › D. Obstructive Sleep Apnea Module/scormcontent/assets/7nn6fW1mU-F6A6DH_047_full.png]

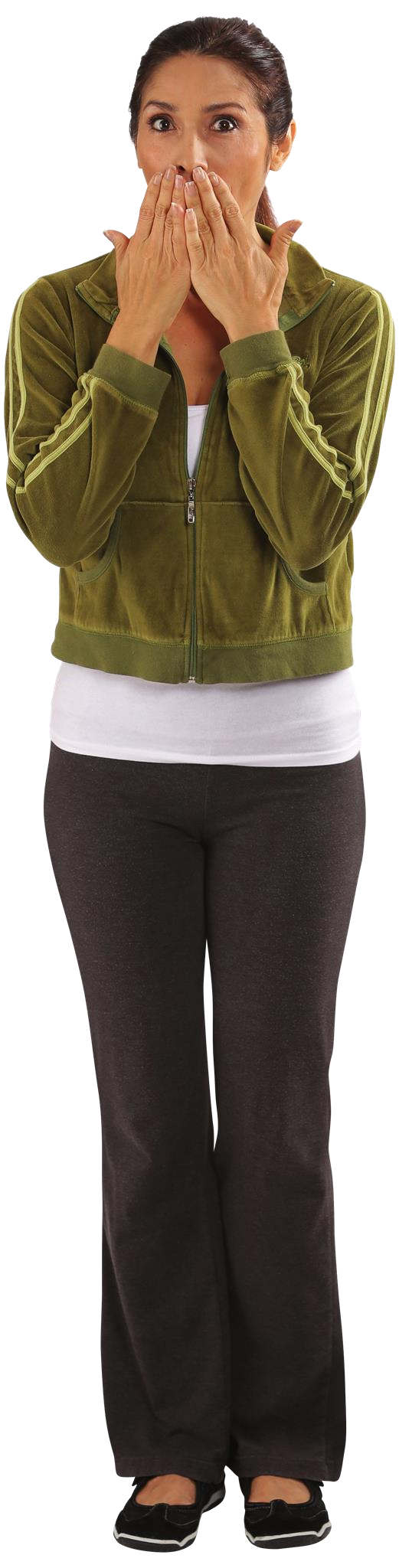

Supplement: Supplementary file 1 — Asthma Module folderTracheostomy Module folderChronic Cough Module folderObstructive Sleep Apnea Module folderPosttest Questions.docxFeedback.docx [file mep_2374-8265.11470-s001.zip › D. Obstructive Sleep Apnea Module/scormcontent/assets/8bICRLpdu6ZiWkMW_104_full.png]

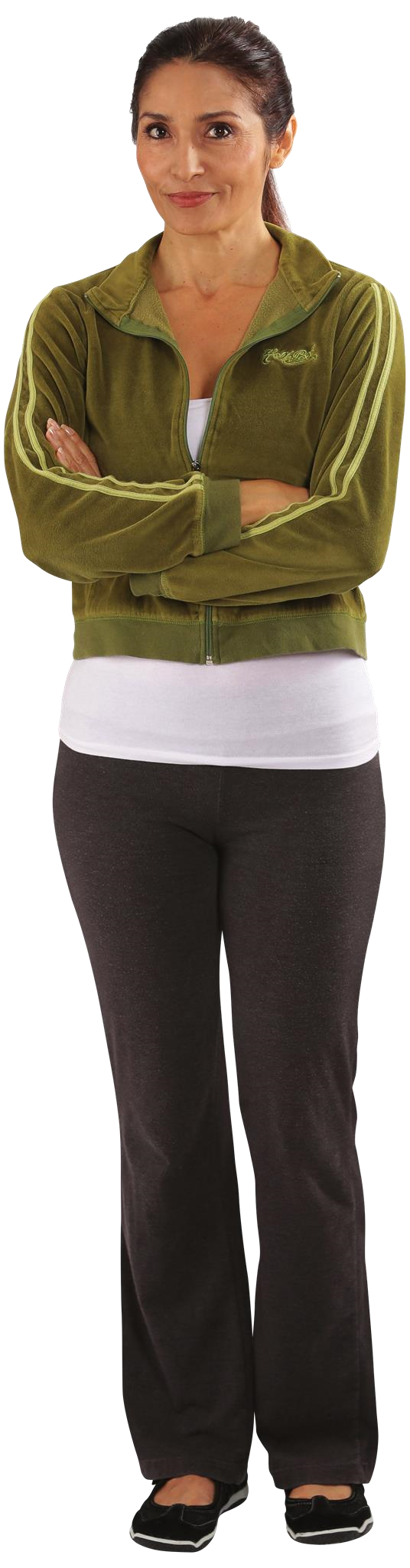

Supplement: Supplementary file 1 — Asthma Module folderTracheostomy Module folderChronic Cough Module folderObstructive Sleep Apnea Module folderPosttest Questions.docxFeedback.docx [file mep_2374-8265.11470-s001.zip › D. Obstructive Sleep Apnea Module/scormcontent/assets/B18Cp-iPjIVnbg6c_114_full.png]

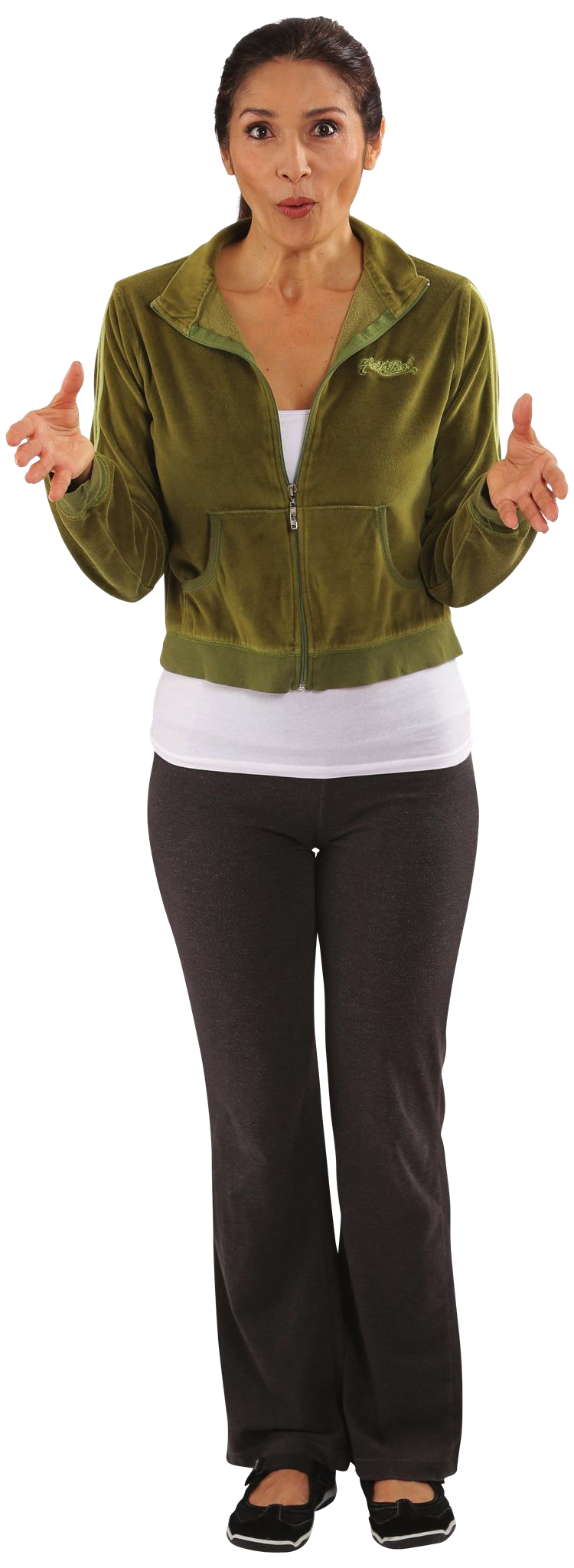

Supplement: Supplementary file 1 — Asthma Module folderTracheostomy Module folderChronic Cough Module folderObstructive Sleep Apnea Module folderPosttest Questions.docxFeedback.docx [file mep_2374-8265.11470-s001.zip › D. Obstructive Sleep Apnea Module/scormcontent/assets/B5hTdJqJm2QwwVSj_053_full.png]

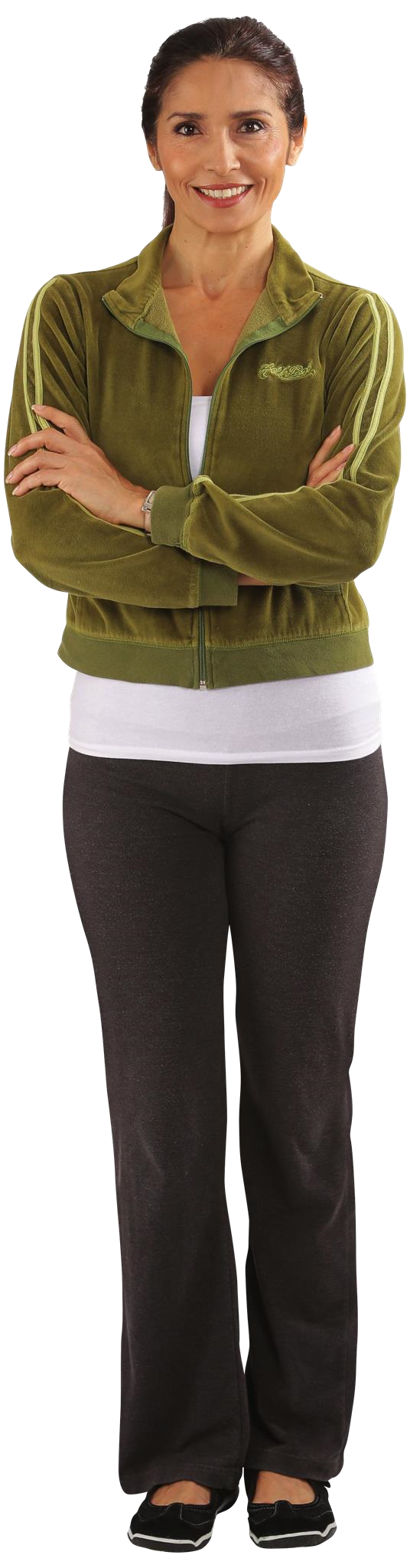

Supplement: Supplementary file 1 — Asthma Module folderTracheostomy Module folderChronic Cough Module folderObstructive Sleep Apnea Module folderPosttest Questions.docxFeedback.docx [file mep_2374-8265.11470-s001.zip › D. Obstructive Sleep Apnea Module/scormcontent/assets/BIe8zpQS0oRoHOsR_008_full.png]

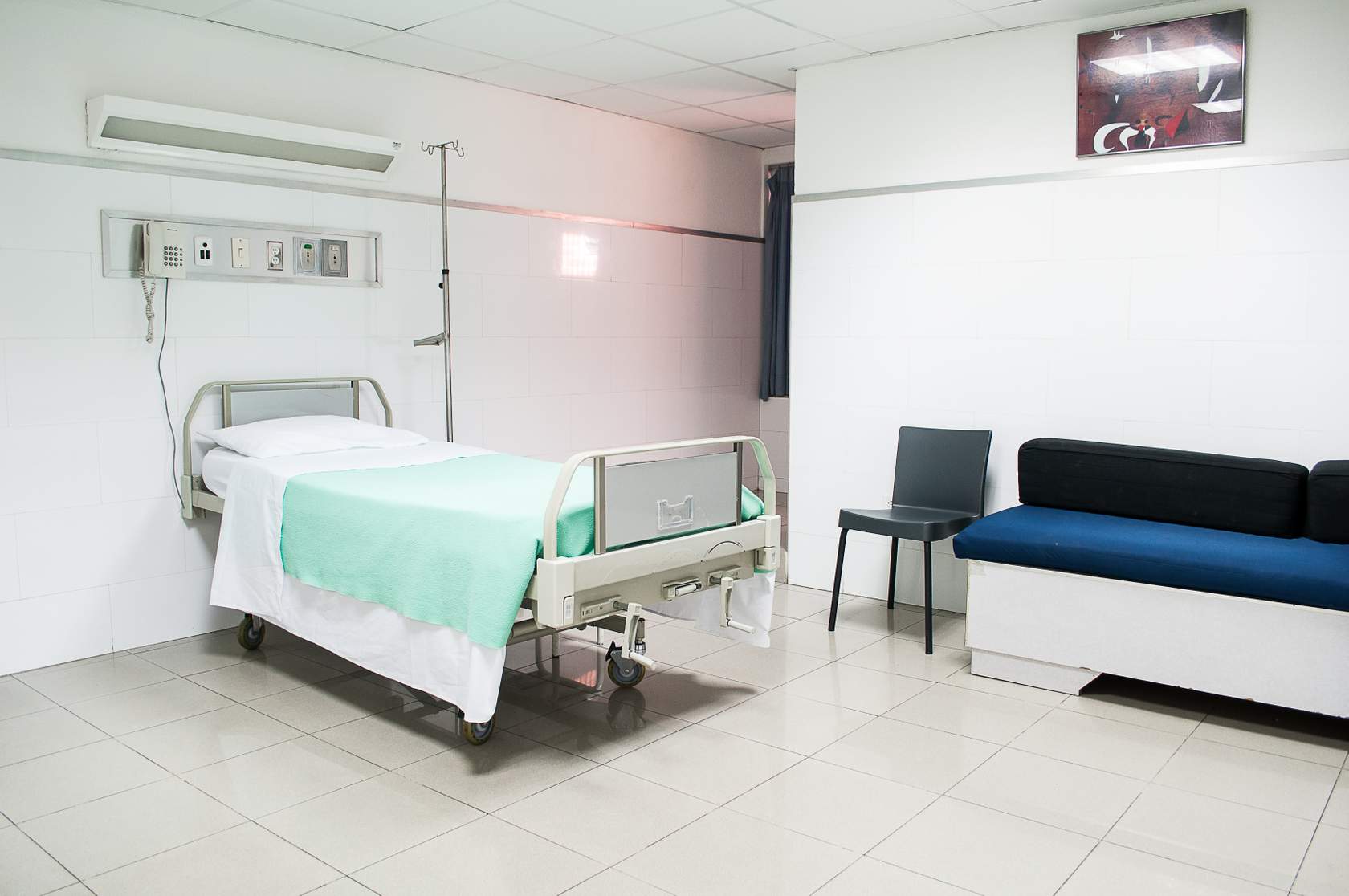

Supplement: Supplementary file 1 — Asthma Module folderTracheostomy Module folderChronic Cough Module folderObstructive Sleep Apnea Module folderPosttest Questions.docxFeedback.docx [file mep_2374-8265.11470-s001.zip › D. Obstructive Sleep Apnea Module/scormcontent/assets/bkiaJC/stock-image.jpg]

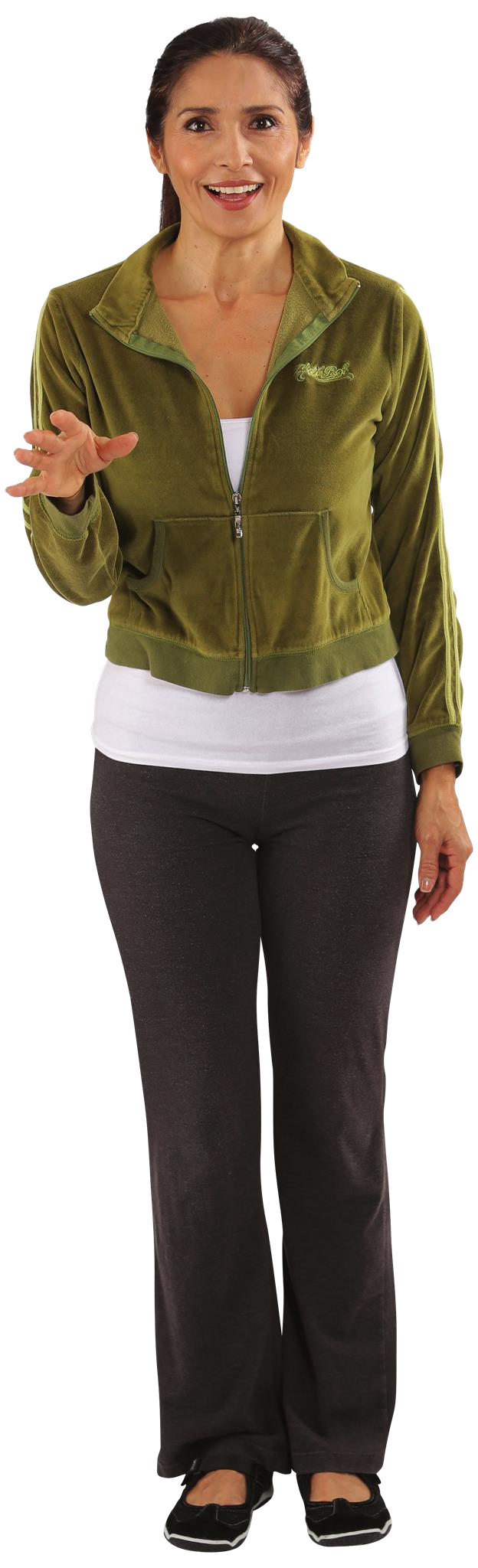

Supplement: Supplementary file 1 — Asthma Module folderTracheostomy Module folderChronic Cough Module folderObstructive Sleep Apnea Module folderPosttest Questions.docxFeedback.docx [file mep_2374-8265.11470-s001.zip › D. Obstructive Sleep Apnea Module/scormcontent/assets/Br0ZGMlWhFbjGUNG_049_full.png]

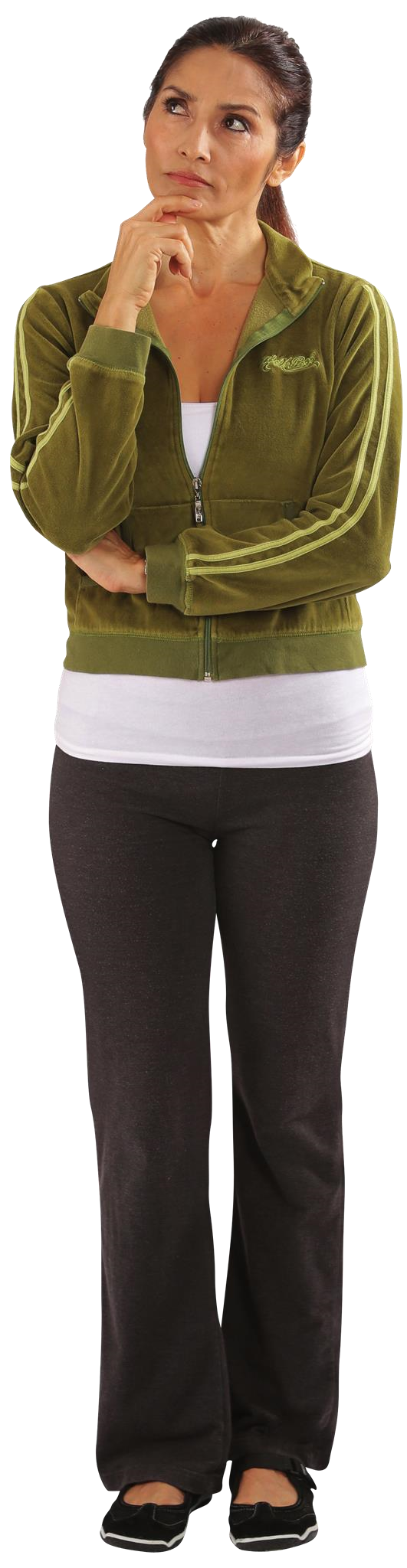

Supplement: Supplementary file 1 — Asthma Module folderTracheostomy Module folderChronic Cough Module folderObstructive Sleep Apnea Module folderPosttest Questions.docxFeedback.docx [file mep_2374-8265.11470-s001.zip › D. Obstructive Sleep Apnea Module/scormcontent/assets/g8vD1WvIcgHp2Dhd_142_full.png]

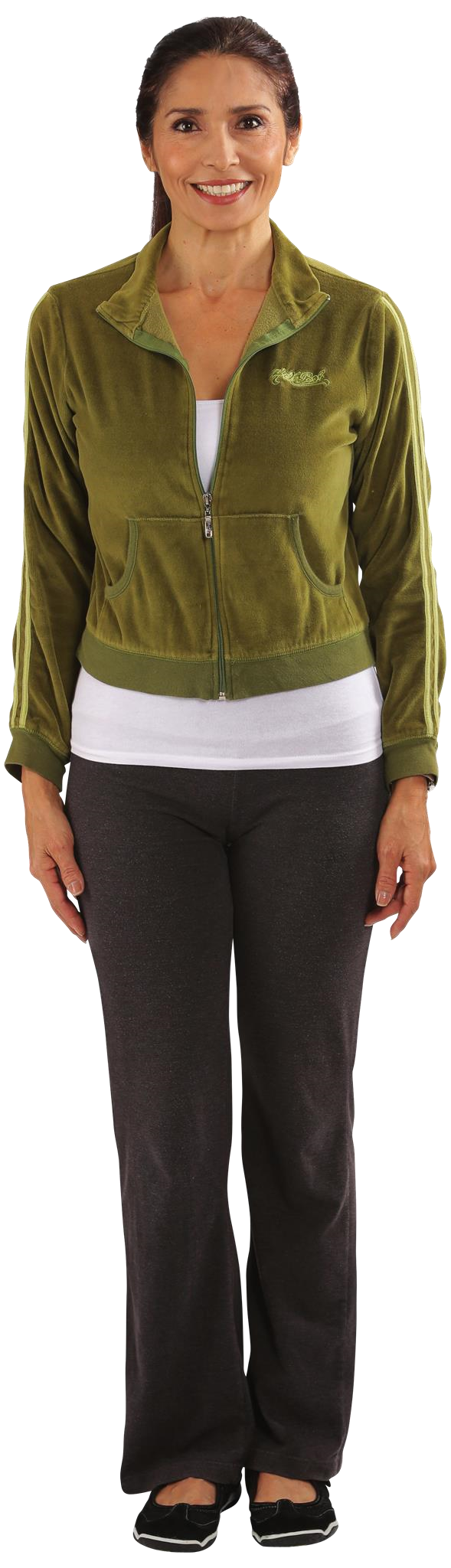

Supplement: Supplementary file 1 — Asthma Module folderTracheostomy Module folderChronic Cough Module folderObstructive Sleep Apnea Module folderPosttest Questions.docxFeedback.docx [file mep_2374-8265.11470-s001.zip › D. Obstructive Sleep Apnea Module/scormcontent/assets/HCAiK1JI9Gw2ABpg_007_full.png]

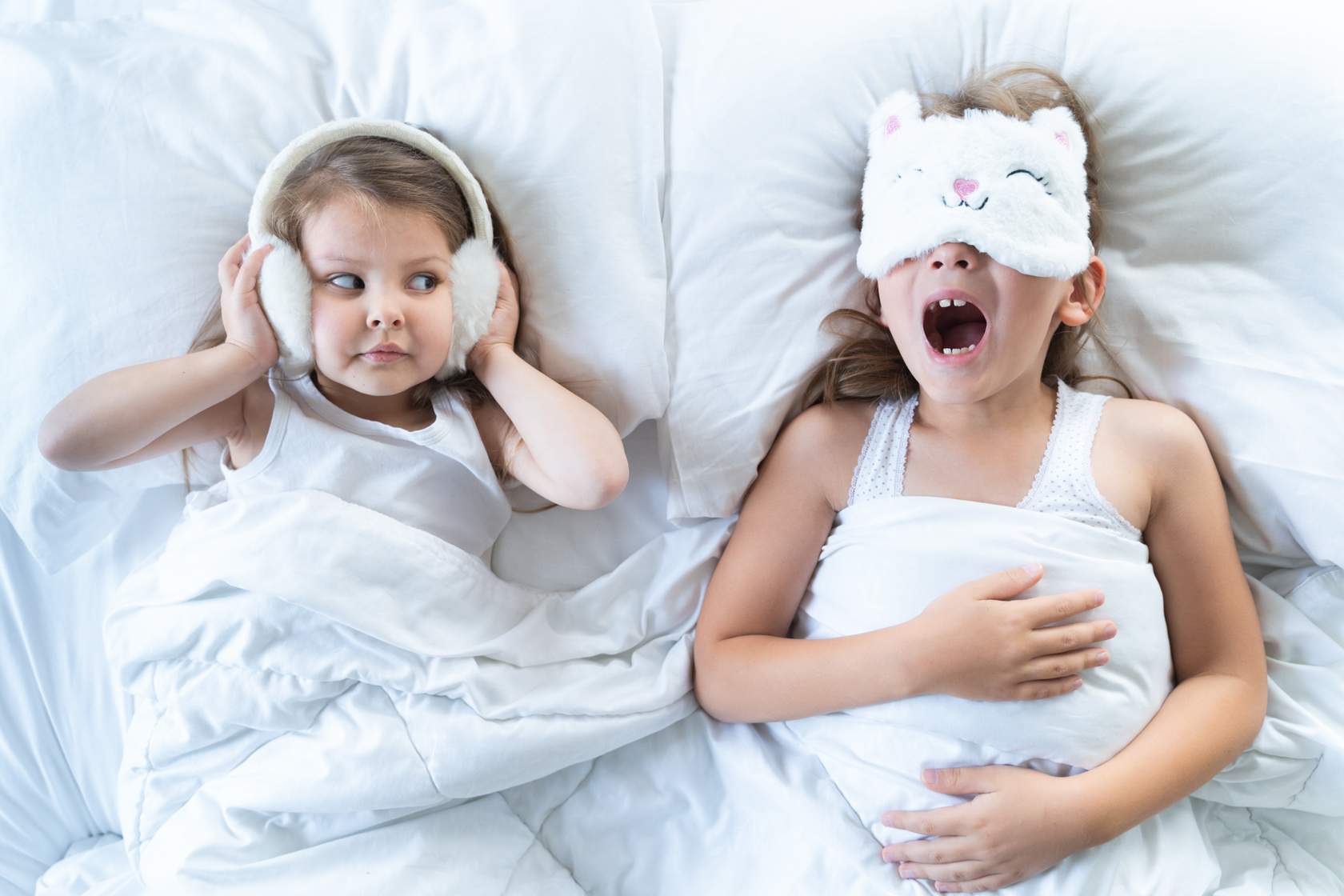

Supplement: Supplementary file 1 — Asthma Module folderTracheostomy Module folderChronic Cough Module folderObstructive Sleep Apnea Module folderPosttest Questions.docxFeedback.docx [file mep_2374-8265.11470-s001.zip › D. Obstructive Sleep Apnea Module/scormcontent/assets/ky0m8zFQRyvgjHTp.jpg]

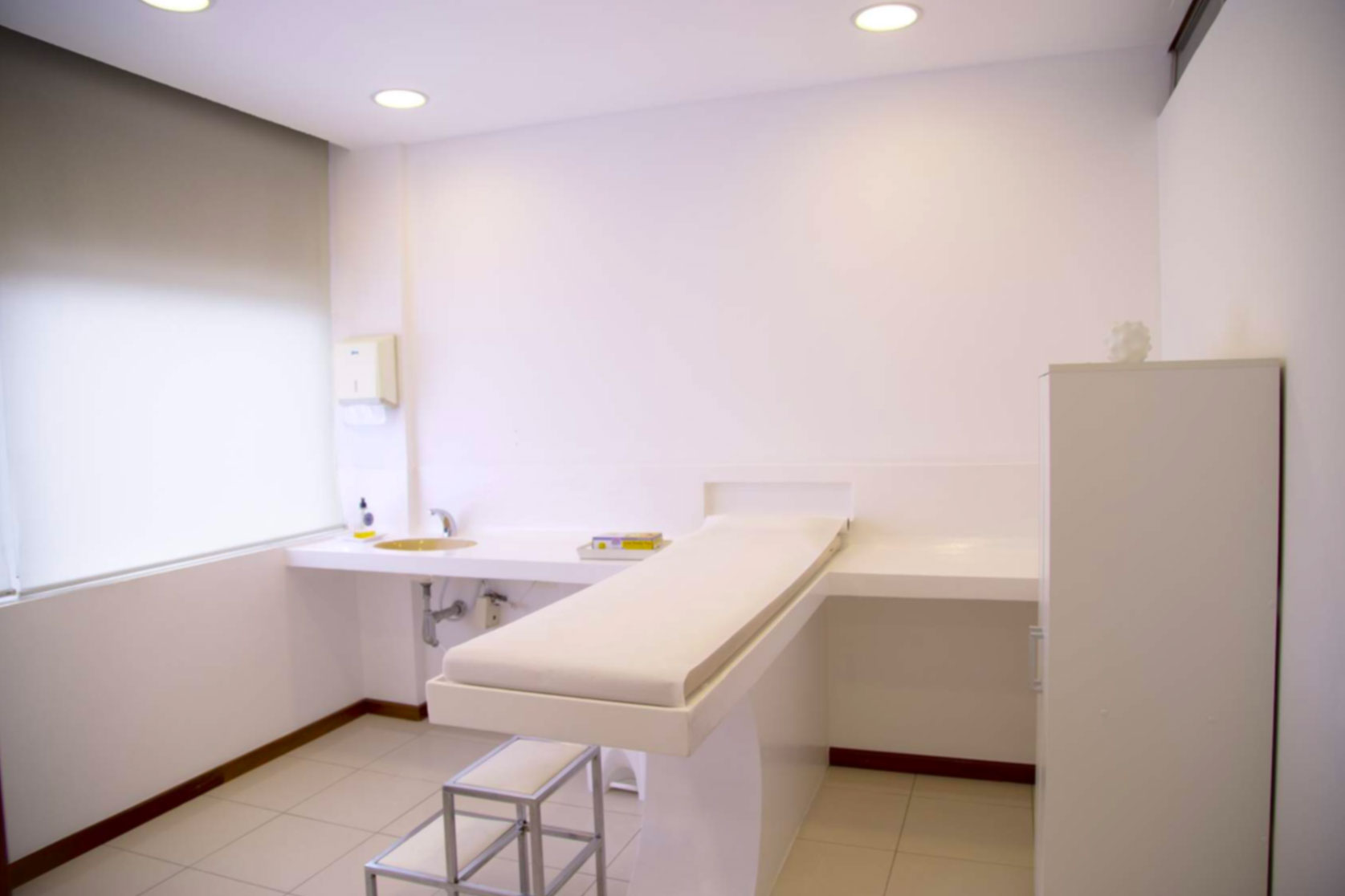

Supplement: Supplementary file 1 — Asthma Module folderTracheostomy Module folderChronic Cough Module folderObstructive Sleep Apnea Module folderPosttest Questions.docxFeedback.docx [file mep_2374-8265.11470-s001.zip › D. Obstructive Sleep Apnea Module/scormcontent/assets/medical.jpg]

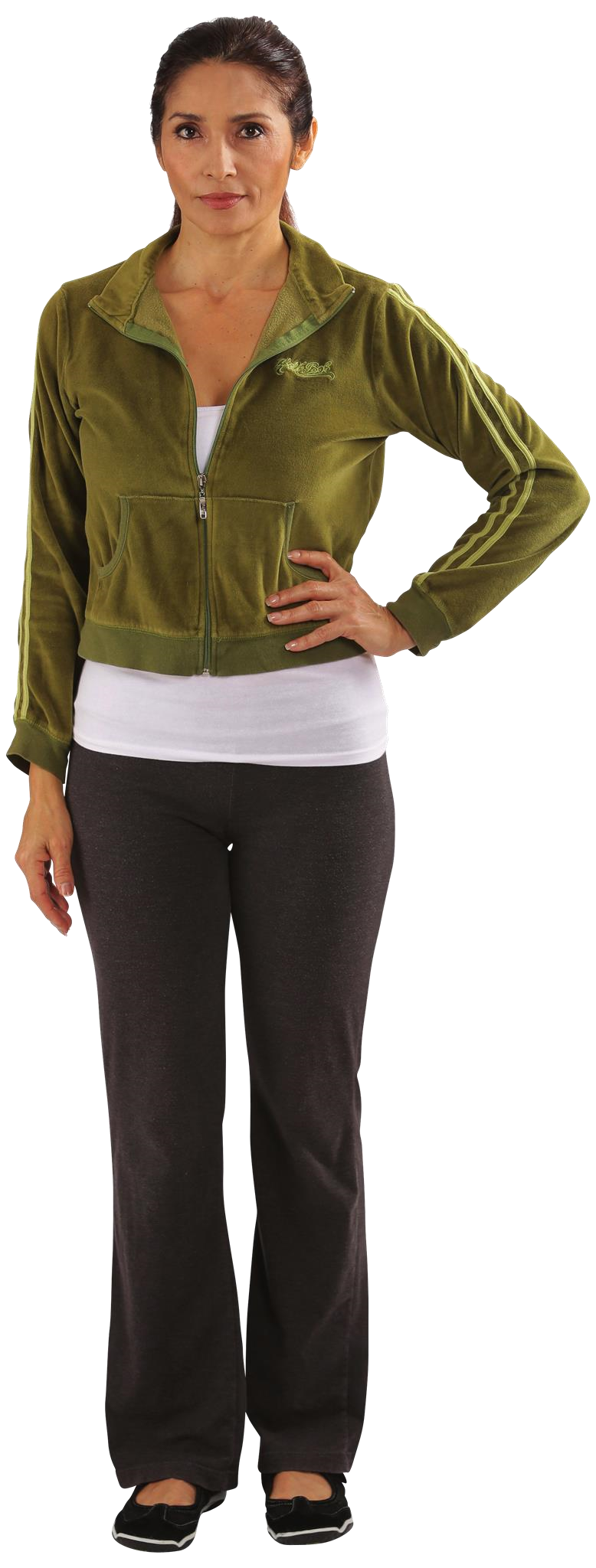

Supplement: Supplementary file 1 — Asthma Module folderTracheostomy Module folderChronic Cough Module folderObstructive Sleep Apnea Module folderPosttest Questions.docxFeedback.docx [file mep_2374-8265.11470-s001.zip › D. Obstructive Sleep Apnea Module/scormcontent/assets/mMf_Eq1JCOew04pv_108_full.png]

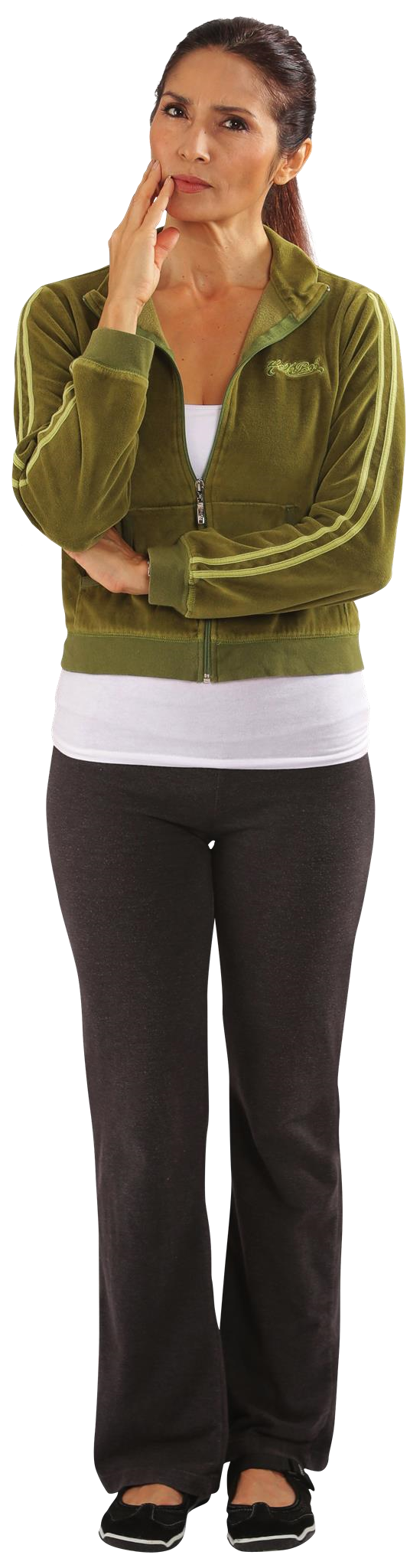

Supplement: Supplementary file 1 — Asthma Module folderTracheostomy Module folderChronic Cough Module folderObstructive Sleep Apnea Module folderPosttest Questions.docxFeedback.docx [file mep_2374-8265.11470-s001.zip › D. Obstructive Sleep Apnea Module/scormcontent/assets/MvoqhBTCUJEqZ-l1_143_full.png]

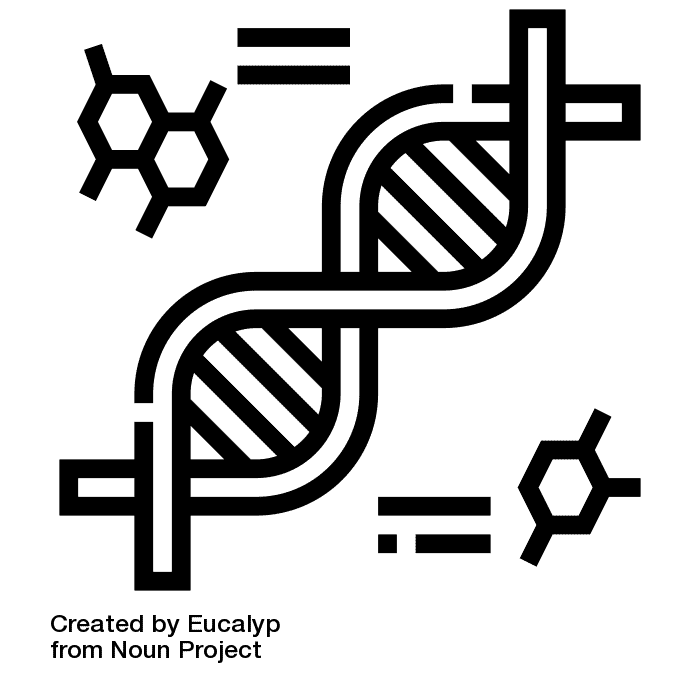

Supplement: Supplementary file 1 — Asthma Module folderTracheostomy Module folderChronic Cough Module folderObstructive Sleep Apnea Module folderPosttest Questions.docxFeedback.docx [file mep_2374-8265.11470-s001.zip › D. Obstructive Sleep Apnea Module/scormcontent/assets/noun_genetic_3218468.png]
