# Supplementary material for: Development and Implementation of a Pediatric Pulmonary–Focused Active Learning Curriculum
Source: MedEdPORTAL. 2024 Dec 6;20:11470. doi: 10.15766/mep_2374-8265.11470 (PMC11621239; doi:10.15766/mep_2374-8265.11470)
Supplement: Supplementary file 1 — Asthma Module folderTracheostomy Module folderChronic Cough Module folderObstructive Sleep Apnea Module folderPosttest Questions.docxFeedback.docx [file mep_2374-8265.11470-s001.zip › E. Posttest Questions.docx]

Asthma Posttest

Q1 A mother brings her 9 year old boy to your clinic because he has been complaining of being tired in physical education class at school for the past few months. When you ask him about his symptoms, he reports having trouble catching his breath after he runs approximately 10 minutes after he starts running. He also has a cough that occurs primarily at night several times a month. He has grown well, and findings on physical examination are normal. Of the following, the MOST likely reason for his exercise intolerance is:

- Cystic fibrosis
- exercise-induced asthma
- iron deficiency anemia
- vocal cord dysfunction
- Wolff-Parkinson-White syndrome

Q1 answer
Answer: exercise induced asthma

Exercise intolerance is the failure to tolerate physical exercise at a level that would be expected for a person’s age and condition, such as described for the boy in the vignette. For the child, it is important to determine whether exercise intolerance is due to a primary pulmonary or extrapulmonary cause. Pulmonary causes include asthma, cystic fibrosis, and acute and chronic infections of the lung.
Among the extrapulmonary causes of exercise intolerance are cardiac disorders such as congestive heart failure, neuromuscular disorders such as muscular dystrophy, anemia, and deconditioning. 

The shortness of breath after running and a nighttime cough described for the boy in the vignette make exercise-induced bronchoconstriction (EIB), also called exercise-induced asthma, the most likely diagnosis. Children who have EIB generally experience shortness of breath, chest tightness, and cough approximately 10 to 15 minutes after beginning exercise. Administration of a short-acting beta2 agonist prior to exercising can help to prevent the symptoms. For patients who have poorly controlled asthma and experience EIB, the most appropriate management is the use of inhaled corticosteroids and possibly other maintenance medications to control overall asthma symptoms.

Vocal cord dysfunction is the paradoxic adduction of the vocal cords during inspiration, causing airway obstruction during exercise. Inspiratory wheezing and throat tightness are common symptoms, but cough at night is not. Cystic fibrosis is unlikely in any child who is growing well and has no extrapulmonary symptoms. Iron deficiency anemia can cause exerciseinduced dyspnea, but the boy’s history is not suggestive of this condition. Wolff-Parkinson-White syndrome causes re-entrant tachycardia; syncope rather than exercise intolerance is the usual clinical manifestation.

Q2 A 9-year-old boy who has mild persistent asthma presents to the urgent care with a 5- day history of cough and shortness of breath that started after a viral upper respiratory tract infection. He has used her beta2 agonist metered dose inhaler (MDI) and inhaled corticosteroids twice each day without significant improvement. On physical examination, he coughs frequently but is sitting comfortably. His vital signs include a temperature of 36.9°C, respiratory rate of 28 breaths/min, heart rate of 120 beats/min, and a pulse oximetry reading of 95% on room air. He has scattered expiratory wheezing on auscultation but no crackles, retractions, or stridor. A chest radiograph shows some mild hyperinflation but also opacification of the right middle lobe. 

Of the following, the MOST appropriate treatment for this boy is to

- admit her to the hospital for intravenous corticosteroids
- continue her current medication
- double her inhaled steroid dose
- start a 3- to 5-day course of oral corticosteroids
- start a 7- to 10-day course of antibiotics

Answer: start a 3- to 5-day course of oral corticosteroids

The boy described in the vignette is suffering an exacerbation of her asthma. Signs and symptoms of asthma exacerbations include cough, wheezing, shortness of breath, hypoxia, tachypnea, and atelectasis. Treatment depends on the age of the patient, current medication use, and severity of the episode. 

For mild exacerbations treated at home, high doses of inhaled corticosteroids may avert the progression of symptoms. For moderate-to-severe exacerbations, as experienced by this patient, oral or intravenous corticosteroids are required. A 3- to 5-day course of oral steroids is adequate for most children, providing a similar outcome compared with intravenous steroids. The need for hospital admission typically is based on the response to therapy in the clinic or emergency department. 

Hospital admission would be considered if a patient presents with cyanosis, apnea, history of previous intensive care unit admission or respiratory failure, peak expiratory flow less than 60% of predicted after beta2 agonist therapy, increased Pco2 on blood gas analysis, or requirement for persistent nebulization therapy or supplemental oxygen. 

Clear manifestations of a bacterial infection should be present before beginning antibiotic therapy during an asthma exacerbation. Because viral infections are the most common trigger in younger children, as described in the vignette, antibiotics are not indicated. 

Chest radiography for the boy in the vignette documents right middle lobe atelectasis, characterized by a wedge-shaped density that extends anteriorly and inferiorly from the hilum of the lung. This finding often is diagnosed inappropriately as pneumonia and treated with antibiotics. Atelectasis due to mucus plugging is not an uncommon finding in children who have asthma exacerbations. 
Right middle lobe atelectasis and other scattered areas of atelectasis usually respond to standard asthma therapy (eg, oral corticosteroids, inhaled asthma medications) and do not require bronchoscopy or chest physiotherapy.

Q3 A 12-year-old girl presents with a 6-month history of increasing cough, dyspnea, and wheezing. She was diagnosed with "asthma" as a child, but reports only using a short-acting beta agonist (SABA). Despite using her SABA three to four times per day, she reports nightly asthma symptoms, significant limitations in physical activity, and frequent asthma symptoms throughout the day. Spirometry performed in the clinic shows significant obstruction, with a forced expiratory volume in 1 second of 55%. 
Of the following, the MOST appropriate asthma medication to consider starting in this child is

- high-dose inhaled steroid and long-acting beta agonist
- a medium-dose inhaled steroid and oral leukotriene antagonist
- an oral leukotriene antagonist only
- omalizumab
- oral theophylline and daily SABA

Answer: high-dose inhaled steroid and long-acting beta agonist

Based on the National Asthma Education and Prevention Program Expert Panel Report 3 Guidelines for the Diagnosis and Management of Asthma, the 12-year-old girl described in the vignette has severe persistent asthma.

A stepwise approach is used to identify the severity of asthma in a new patient and determine the level of control. This stepwise approach accounts for patient’s treatment responses and changes that often occur over time. The choice and schedule of medications is determined by the level of asthma severity and asthma control. Severe persistent asthma in children 12 years of age or older requires the initiation of step 4 or step 5 therapy.

The preferred treatment includes a medium- to high-dose inhaled corticosteroid plus a long-acting beta agonist (LABA). 

Omalizumab is an additional therapy that could be considered for this girl if she had allergic asthma, with evidence of specific sensitization to associated allergens and an appropriate total serum immunoglobulin E concentration.

Q4 You are evaluating a 12-year-old boy for his yearly well child check. He is generally healthy, except for mild seasonal allergic rhinitis that is controlled with an as-needed second-generation antihistamine. However, his mother has noted that over the past 6 months he has awakened coughing 1 to 2 nights a month. In addition, once or twice a month she has noticed that he "wheezes" during physical exertion, although he is still able to continue playing soccer. A pulmonary function test in the office reveals age-appropriate lung function with a normal forced expiratory volume in 1 second (FEV1) (>80%) and normal FEV1/forced vital capacity ratio. You suspect asthma.

Of the following, the MOST appropriate classification for this boy’s asthma is

- exercise-induced
- intermittent
- mild persistent
- moderate persistent
- severe persistent

Answer: Intermittent

The 2007 National Asthma Education and Prevention Program Expert Panel Report 3 for the Diagnosis and Management of Asthma (ERP3) continues to provide a framework for the determination of asthma severity and the initiation of appropriate therapy.

 Based on an assessment of the impairment and risk, the severity of asthma experienced by the 12-year-old boy described in the vignette is categorized as intermittent.
  Characteristics of intermittent asthma, as seen in the child in the vignette, include normal pulmonary function test results, daytime symptoms fewer than 2 days per week, and nighttime symptoms fewer than 2 days per month.    Step 1 therapy for intermittent asthma involves using an as-needed short-acting bronchodilator. Once a child is diagnosed with asthma, the frequency of follow-up can be based on asthma severity and level of control.    Exercise is a common trigger of asthma for children. Approximately 70% to 90% of children who have asthma are believed to have symptoms of exercise-induced asthma (EIA). Although the child in the vignette may have exercise-induced symptoms, a classification of EIA is less accurate than intermittent asthma because the child has symptoms outside of exercise (ie, at night). Because up to 40% of children who have allergic rhinitis have some degree of asthma and 60% to 80% of children who have asthma have allergic rhinitis, asking about other atopic diseases is recommended.

Q5 A medical student you are supervising requests your assistance interpreting the results of spirometry on a patient who has persistent asthma. The student would like to learn about the lung function parameters measured during spirometry that aid in the diagnosis of asthma. You review the interpretation of the spirometry results (ie, forced expiratory volume in 1 second [FEVI] to forced vital capacity [FVC] ratio, FEV, and post bronchodilator reversibility) with the student.

Of the following, the findings MOST consistent with this diagnosis are

- FEV, to FVC ratio < 80% and FEV	(1)
- FEV, to FVC ratio < 80% and FVC < 80%; postbronchodilator reversibility of 12% (2)
- FEV, to FVC ratio > 80% and FVC < 80%; postbronchodilator reversibility of 20% (3)
- FEV, to FVC ratio > 80% and FEV, < 80%; postbronchodilator reversibility of 12% (4)
- FEV, to FVC ratio > 80% and FEV, < 80%; postbronchodilator reversibility of 20% (5)

**Answer: FEV1 to FVC ratio <80% and FEV1 <80% post bronchodilator reversibility of 12%**

The spirometric findings most consistent with the diagnosis of asthma are forced expiratory volume in 1 second [FEV1] to forced vital capacity [FVC] ratio less than 80%, FEV1 less than 80%, and postbronchodilator reversibility of 12%. The 2007 National Heart, Lung, and Blood Institute (NHLBI) Guidelines for asthma recommend performance of spirometry as an essential objective measure to establish the diagnosis of asthma. Spirometry can demonstrate obstruction and assess reversibility in patients at least 5 years of age. Spirometry is also helpful in monitoring the response to long-term therapy and changes in the degree of obstruction over time. The important parameters derived from spirometry include indices of flow, including forced expiratory volume in one second (FEV1), flow between 25% and 75% of the vital capacity (FEF25-75), also known as the maximal mid-expiratory flow rate, and peak expiratory flow rate (PEFR). FEV1, FEF25-75, and PEFR are decreased in obstructive disorders. Indices of volume are also measured, including forced vital capacity (FVC). Forced vital capacity is the volume of air that can be forcibly blown out after full inspiration, measured in liters. FEV1 is the volume of air that can be forcibly blown out in 1 second, after full inspiration. Values from 80% to 120% of the average value are considered normal. FEV1/FVC (FEV1%) is the ratio of FEV1 to FVC. In healthy children, this should be 80% or greater. In obstructive diseases FEV1 is diminished because of increased airway resistance to expiratory flow. In some cases, the FVC may be decreased as well, owing to the premature closure of airway in expiration, but the FEV1 is typically disproportionately affected. In restrictive diseases the FEV1 and FVC are both reduced proportionally and the ratio may be normal or even increased as a result of decreased lung compliance. An FEV1/FVC ratio less than 80% in children 8 to 19 years of age therefore suggests that the observed decrease in airflow is due to airway obstruction rather than due to volume reduction. Reversibility is determined either by an increase in FEV1 of at least 12% from baseline or by an increase of at least 10% of predicted FEV1 after inhalation of a short acting bronchodilator.

Chronic Cough Posttest

Q1 A 3-year-old healthy girl is brought to your office for the second time this week because of a cough and intermittent wheezing that started 1 week ago. A review of her chart shows that your partner prescribed inhaled albuterol for wheezing at her initial visit 5 days ago. Her symptoms have not improved and mom feels like her cough seems to be worsening. This morning she coughed up a small amount of blood. Physical examination reveals a well-nourished, interactive girl who is coughing frequently and has wheezing localized to the right lower lung field. The mother asks you if her daughter's symptoms could indicate a food allergy, since her cough began shortly after she ate peanuts for the first time. 
Of the following, the study MOST likely to confirm the patient's diagnosis is

- bronchoscopy
- pulmonary function testing
- skin testing for peanut allergy
- sputum culture
- sweat chloride test

Answer: A: bronchoscopy 
  
The girl described in the vignette presents with a history and clinical findings that strongly suggest the diagnosis of foreign-body aspiration. Of the diagnostic studies listed, bronchoscopy is most likely to confirm her diagnosis. Furthermore, rigid bronchoscopy is the standard of care for treatment of foreign body aspiration. Foreign-body aspiration should be suspected in any patient presenting with respiratory distress and/or wheezing of sudden onset, even in the absence of a clear history of a choking episode. A detailed history regarding the circumstances surrounding the onset of wheezing is very important. Although a history of choking is highly suggestive of foreign-body aspiration, it is not witnessed or recalled in all cases. Foreign-body aspiration is most common in children younger than 3 years. 
 
The classic triad of coughing, wheezing, and decreased breath sounds is present in only approximately 40% of cases. Wheezing is a common presenting symptom of respiratory disease in children. Although most instances of wheezing arise from infectious causes or asthma, it is crucial for pediatric practitioners to understand the extensive differential diagnosis for wheezing and to appreciate that "all that wheezes is not asthma"
 
Foreign-body aspiration should be suspected in any child with a unilateral monophonic wheeze or asymmetric breath sounds. Regional variation in air entry is an important clue to the diagnosis. In children, an aspirated foreign body may lodge in any bronchus, without predilection to the right side. In some cases, an aspirated foreign body may trigger a generalized irritant response, resulting in diffuse polyphonic wheezes. Other associated symptoms may include persistent cough, hemoptysis, dyspnea, choking, fever, and even cyanosis. If diagnosis is delayed, patients with foreign-body aspiration may develop pneumonia.
 

Q2 You are evaluating a 3 year old child with 10 day history of coughing. The cough is frequently heard during the day and night. The child has had no fever, wheezing, or chest pain. The cough is dry and he has no posttussive emesis. He has been otherwise healthy. There is no history of FB aspiration or choking episodes. However he did have symptoms of viral URI when he began coughing. His physical exam findings are normal except for an occasional dry cough and nasal congestion. 
Of the following, the most appropriate next step in management is:

- Obtain CXR
- Obtain pre/post bronchodilator spirometry
- Reassure the parents that his cough will likely resolve without treatment
- Start treatment with broad spectrum antibiotics

Answer C: Reassure the parents that this cough will likely resolve without treatment

 The most frequent cause of acute coughing in children is acute viral upper respiratory tract infection. The child in this vignette is coughing because of recent URI and needs no further diagnostic or therapeutic intervention. The majority of children with upper respiratory tract viral infections have associated coughing. The cough can outlast the other signs and symptoms of an upper respiratory tract infection and can interfere with sleep and be disruptive to the family and the classroom. 

 Most episodes of acute coughing other than foreign body aspiration resolve on their own within 2-3 weeks without the need for diagnostic or therapeutic interventions. Because this child does not appear ill or has fever, it is not necessary to obtain CXR to rule out pnemonia or treat with antibiotics.

 Most 3 year old children cannot reliably perform spirometry. In addition, this is no history of persistent or recurrent symptoms to indicate underlying asthma. 

Q3 A 9 month old has had a chronic cough for 6 months. The cough is wet and most obvious with oral intake. There is no vomiting with oral intake. He is gaining weight appropriately. He has had 2 episodes of pneumonia that were treated with oral antibiotics. He was born at 30 weeks gestation and developed RDS that required intubation and mechanical ventilation for 6 weeks. He appears well and has normal vital signs. There are transient wheeze following drinking from a bottle. 

Of the following, what is the next best step?

- Chest CT
- Sweat chloride test
- video fluoroscopic swallow study
- 24 hour esophageal impedance monitoring

Answer C : video fluoroscopic swallow study 

This infant most likely to have chronic recurrent aspiration from dysphagia, which is suggested by the wet cough with oral intake an history of pneumonias in the absence of symptoms of gastroesophageal reflux (GER). This condition is best diagnosed by videofluoroscopic swallow study. 

Chest CT can be useful in evaluation of child with aspiration, but findings are typically not diagnostic.

This infant's history of prematurity and history of intubation are risk factors for dysphagia, which can cause chronic aspiration. 

Recurrent aspiration of food, oral/nasal secretions, and gastric contents can lead to both acute and chronic respiratory symptoms. Symptoms include chronic cough, recurrent wheeze, recurrent pneumonia, and apnea. Choking, gagging, and coughing can occur with oral intake. Atelectasis, bronchiectasis, pulmonary abscess, pulmonary fibrosis, bronchiolitis obliterans, failure to thrive, stridor, and hoarse voice can all occur secondary to chronic aspiration. In addition, aspiration can be silent, particularly in neurologically impaired children. 

Q4 A 2 year old presents with chronic cough and nasal congestion. Mom said the cough has been present since he was 3 months old and always sounds wet. He has received multiple rounds of antibiotics which helps, but his cough returns shortly after completing antibiotics.  He also has a history of hearing loss. He is coughing in your office and you hear diffuse crackles throughout on his lung examination. You send him for a chest XR and it shows dextrocardia. 
What is the diagnosis?  

- Cystic fibrosis
- Primary ciliary dyskinesia
- Community acquired pneumonia
- Protracted bacterial bronchitis

Q8 Answer B:  Primary Ciliary Dyskinesia 
 
This child most likely has primary ciliary dyskinesia. Primary ciliary dyskinesia (PCD) is usually an autosomal recessive genetic condition in which the microscopic organelles (cilia) in the respiratory system have defective function. 
 
Symptoms often begin shortly after birth and can include coughing, gagging, choking and lung atelectasis (neonatal respiratory distress). Affected individuals often experience chronic sinus, middle ear and lung infections as well as chronic coughing, excess mucus and hearing loss. The recurring respiratory infections can lead to an irreversible scarring and dilatation in the bronchi (bronchiectasis) and severe lung damage.
 
 Affected males are typically infertile because movement of sperm (motility) is abnormal. PCD may also be associated with infertility and ectopic pregnancy in females. Movement of cilia may also be important in organ placement in the developing embryo. Approximately 50% of individuals with PCD have Kartagener syndrome in which the internal organs including the heart, liver, spleen and intestine are on the opposite side of the body (situs inversus totalis). Some individuals with PCD have a condition called heterotaxy (situs ambiguus) in which internal organs are abnormally positioned and have abnormal structure. Approximately, 12% of the PCD patients present with heterotaxy and a subset of those have congenital heart defects that can be serious and life threatening.
Q5 A 9 year old presents with a 2 year history of chronic cough interspersed with some periods of relief. The cough is harsh and barky. She has been prescribed multiple courses of steroids and antibiotics which have not helped. Mother notes that she does not cough during sleep. On physical exam, she is coughing intermittently. You distract her by talking to her about her favorite movie and notice that the cough is less frequent. Lungs sound clear on exam. 
What is the next step in management?

- spirometry with bronchodilator response
- CXR
- Inhaled daily steroids
- Behavioral therapy
- Reassurance

Answer D: Behavioral therapy 

 There are many causes of chronic cough in childhood. This girl most likely has habit cough and behavioral therapy is the best treatment for it.  The cough usually begins after an upper respiratory infection, but the cough persists long after the other respiratory symptoms resolve. Typically, the cough is harsh, barking, and nonproductive. Characteristically, it disappears during sleep or distraction and is not exacerbated by physical exercise.
 
The diagnosis of habit cough requires a thorough evaluation to rule out other causes of chronic cough. 

 The mean age for this disorder has been identified as 10 years. Eighty-five percent of 120 children at University of Iowa program were between 8 and 14 years of age.

 Various therapeutic approaches have been proposed, including explanation and reassurance, hypnosis, and suggestion therapy. 
 
Habit cough may be associated with psychosocial stressors. The most common are school problems as the frequent loud coughing can be very disruptive in a classroom and may lead to school absenteeism, which can reinforce the cough.

OSA Posttest

Q1 A 5-year-old boy presents with a 2 year history of chronic nasal congestion throughout the year. His parents describe him as a "constant mouth breather," with nightly snoring and occasional episodes where he has stopping breathing. These symptoms have occurred daily and have not improved after a 3 month course of nasal corticosteroids and second-generation oral antihistamine. The child has no ocular symptoms and has little to no sneezing or nasal itching. He is otherwise healthy other than recurrent episodes of otitis media. On physical examination, the child is mouth breathing, but otherwise appears healthy. On evaluation of his head and neck, you note tonsils that extend beyond the folds of the pharyngeal wall but do not touch. 


Of the following, the MOST likely reason for this boy’s symptoms is

- adenoidal hypertrophy
- chronic sinusitis
- nasal polyposis
- perennial allergic rhinitis
- seasonal allergic rhinitis

Answer: Adenoidal hypertrophy 

The clinical history and symptoms described for boy in the vignette are suggestive of adenoidal hypertrophy which is the most common upper airway obstructive lesion in children. 

Adenoidal hypertrophy presents most commonly with nasal obstruction and persistent mouth breathing. Over time, chronic mouth breathing may lead to changes in facial appearance (so-called "adenoidal facies"), obstructive sleep apnea syndrome, snoring, recurrent sinusitis or otitis media, and hyponasal speech. Oftentimes, the tonsillar tissue is enlarged but may appear appropriate in the presence of adenoidal hypertrophy. An adenoidectomy/tonsillectomy usually is recommended in the presence of recurrent sinusitis or otitis media or obstructive sleep apnea syndrome. 

Allergic rhinitis due to perennial allergens (eg, house dust mite, pet dander, molds) can develop within the first postnatal year but typically presents with more classic symptoms (ie, sneezing, rhinorrhea, ocular pruritus) and responds to combinations of oral antihistamines and nasal corticosteroids. Seasonal allergic rhinitis (eg, trees, weeds, grasses) usually does not occur until the fourth or fifth year after birth and also usually responds well to usual allergy medications. The absence of purulent rhinorrhea, facial-dental pain, postnasal drainage, headache, and cough make chronic sinus disease less likely for this boy. 

Nasal polyps in children may cause nasal obstruction and anosmia, but they generally do not cause snoring or sleep apnea symptoms. Most polyps originate from the ethmoid sinuses and appear as gelatinous, pale, grape-like structures that do not change in size or appearance after administration of a nasal decongestant. Children who present with nasal polyposis should be evaluated for cystic fibrosis. Less common causes of chronic nasal symptoms include nonallergic rhinitis, vasomotor rhinitis, cerebrospinal fluid rhinorrhea, nasal sarcoidosis, Wegener granulomatosis, septal deviation, primary ciliary dyskinesia, rebound congestion from chronic nasal decongestant use, and foreign bodies.

Q2 A 7 year old boy has a history of moderate persistent asthma. His mother is concerned that he has been sleeping poorly. He snores loudly with noted pauses and snorting that often wake him up. He has behavior issues at school. His BMI is greater than the 95th percentile and his blood pressure is 128/80. His tonsil are 4+ in size. Vital signs and physical exam are normal. His polysomnography results show an apnea hypopnea index (AHI) of 27 events an hour with an SpO2 nadir of 77% with a normal wake SpO2. 

Of the following, the most accurate statement regarding this boy’s obstructive sleep apnea is:

- An adenotonsillectomy is recommended and he will require inpatient overnight monitoring postop
- Adenotonsillectomy will not improve his symptoms
- He will only need a clinical assessment following Adenotonsillectomy to determine if his sleep apnea has resolved
- Continuous Positive Airway Pressure (CPAP) is indicated as initial management

Answer: Adenotonsillectomy requires inpatient overnight monitoring postop
 
He has severe obstructive sleep apnea that is complicated by his OSA and asthma. He is at high risk for post-op compromise after his T&A based on his polysomnogram findings and of an apnea-hyponea (AHI) index greater than or equal to 24 events/hour and an SpO2 less than 80%. 

 This child in this vignette demonstrates several features of OSA including snoring, observed apnea, and snorting. he is obese with tonsillar hypertrophy and hypertension. 


Although T&A is generally effective in treating OSA, it does not always provide complete resolution, particularly in patients who are obese or have craniofacial abnormalities. 

AAP guidelines consider children younger than 3 years and children with severe OSA, obesity, craniofacial anomlies, neuromuscular disorders, cardiac complications, or failure to thrive as being at higher risk for postsurgical complications including worsening OSA, pulm edema, or death. Therefore guidelines recommends that high risk patients who undergo adenotonsillectomy receive inpatient post-op monitoring.

Q3 You are evaluating a 6 year old boy 1 month after a tonsillectomy and adenoidectomy (T&A). At his first visit 3 months ago, mom described loud snoring with pauses in his breathing and daytime hyperactivity. He had difficulty completing schoolwork. You ordered a polysomnography (PSG) that revealed mild OSA with AHI of 3 events/hr. Today his mother reports quiet breathing and improvement in school. His teacher has also noted an improvement in his behavior. His BMI is 15kg/m2 at the 50th percentile. His lungs are clear to auscultation. 


The medical student who is working with you ask what is the next step in management?

- Weight loss
- Observation is indicated
- Noninvasive positive pressure
- Repeat evaluation by ENT

Answer: Observation is indicated 

The majority of children with OSA experience resolution following T&A with no further testing indicated. 
Patients with severe OSA should have repeat sleep study after full recovery from T&A( typically 2-3 months) to assess of OSA is still present. Additionally those with other comorbidies that may contribute to OSA should undergo repeat study. 
According to a meta analysis of 14 studies, 82.8% of children have normal sleep studies following T&A and most children without obesity of crainofacial abnormalities will have complete resolution.

Q4 A 9 year old presents to his primary care physician for a well child check. Mom tells you he snores at night and is extremely restless. He undergoes a sleep study which reveals mild obstructive sleep apnea. 

Which symptoms or exam findings most likely accompanies this diagnosis?

- Sleep onset insomnia
- Bed-wetting
- Behavior issues, mood swings, attention-deficit/hyperactivity disorder like symptoms
- Excessive daytime sleepiness
- Small tonsil

Answer: Behavior issues, mood swings, attention-deficit/hyperactivity disorder like symptoms
Children, especially children with mild OSA, unlike adults with OSA, are most likely to present with behavioral issues, mood swings, and attention-deficit/hyperactivity disorder (ADHD)-like symptoms.
Adults, not children, usually present with excessive daytime sleepiness.Children with OSA have short sleep latency. The main risk factors for OSA in children are adenotonsillar hypertrophy and obesity.
Children with OSA may present with bed-wetting, but it is not the most common presentation.

Q5 A 5 year old with trisomy 21 presents to the office for a check up. Mom notes that she has very heavy breathing when she is sleeping and frequent night awakenings. On physical exam, mild midface hypoplasia, large tongue and 2+ tonsils. Her lungs are clear. BMI is in the 25itle. 
What is the AAP guideline for referral for polysomnogram for patients with Trisomy 21?

- Only when there are clinical signs of OSA
- At the age of 18
- By the age of 4
- Yearly starting age of 8 years old

Answer:  By the age of 4
 
Children with Down syndrome often have sleep‐disordered breathing. Research suggests that 43%-66% of children with Down syndrome have SDB, a category that encompasses sleep apnea (both obstructive and central) and hypoventilation. Those numbers are several times higher than the prevalence of SDB in children in the general population (1%-5%).

 Anatomy accounts for many of the reasons why there is a higher incidence of obstructive sleep apnea (OSA) in individuals with Down syndrome. Some of those factors include: central apnea, low muscle tone in the mouth and upper airway, poor coordination of airway movements, narrowed air passages in the midface and throat, a relatively large tongue, and hypertrophy (enlargement) of adenoid and tonsillar tissues.  Increased upper airway infections and nasal secretions and a higher incidence of obesity further contribute to collapse and obstruction of both the oropharynx and the hypopharynx when the individual is sleeping.

 Per AAP, "At least once during the first 6 months of life, discuss with parents symptoms of obstructive sleep apnea, including heavy breathing, snoring, uncommon sleep positions, frequent night awakening, daytime sleepiness, apneic pauses, and behavior problems that could be associated with poor sleep. Refer to a physician with expertise in pediatric sleep disorders for examination and further evaluation of a possible sleep disorder if any of the abovementioned symptoms occur." If they have not received a sleep study by the age of 4, all children with DS should be referred for one. 

 As for treatment, adenotonsillectomy was most common, although previous studies have shown that moderate to severe OSA in children with Down syndrome is likely to persist after a tonsillectomy.

**Trach Posttest**

**Q1 A mother calls you about her 3 year old who received mechanical ventilation at home via trach for chronic lung disease. Normally child is well. The mother states that her child has developed “cold” symptoms (coryza, rhinorrhea, and cough) after her older sibling developed similar symptoms. She has required more suctioning during the day and her secretions have changed from clear to creamy. No fever has been noted. O2 sat in RA has been 92% instead of the usual 97%. At night, high pressure alarms of the ventilator have been going off. The mother is concerned and ask about their causes. Of the following.** 

The most likely reason for is the alarm is:

- Central apnea
- Tracheostomy tube occlusion
- Ventilator malfunction
- Worsening hypoxemia

Answer: Tracheostomy tube occlusion 

With upper respiratory tube infections, there is increased number of secretions and mucous which can lead to tracheostomy tube occlusion. The ventilator most likely is sensing increase pressure needed to deliver the set amount of pressure or volume due to a mucous plug at the tip of the tracheostomy tube. 

With any ventilator alarm, it is important to check for position of the trach tube and the patency of it. If there is any resistance with suctioning, it is recommended to change the trach tube immediately. 


**Q2 You are seeing a child in clinic with chronic respiratory failure from severe BPD who is receiving home mechanical ventilation via trach tube. Upon entry into the room, you note that the child pulse oximetry is alarming for saturations of 82% and the heart rate is elevated. The child has a trach tube in place and is retracting and cyanotic. 
Of the following, the best first step in the management of this child is:**

- Administer albuterol via nebulizer
- Provide positive pressure via resuscitation bag
- Reposition pulse ox probe
- Suction the tracheostomy tube

Answer: Suctioning the tracheostomy tube 

First step when seeing a child with a trach in respiratory distress: check position and patency of the trach tube!

Maintaining and ensuring a patent airway by suctioning is a vital component of management for a child with a tracheostomy. Techniques of suctioning are designed to efficiently clear the airway of mucus while avoiding the potential hazards of suctioning.

The technique should also include twirling or rotating the catheter between the fingers and thumb, not stirring the catheter with the entire hand. Twirling the catheter reduces friction, so that the catheter is more easily inserted, and moves the side holes of the catheter in a helix, thereby suctioning secretions off all areas of the tracheostomy tube wall.

 In children with no evidence of secretions, a minimum of suctioning, at morning and bedtime, to check for patency of the tube is recommended.

**Q3 You are working in the NICU and have 4 patients on your team. Which one of these scenarios would a tracheostomy be indicated?**

- 4 month old male with severe subglottic stenosis who remains intubated despite multiple dilations
- 3 month ex-30 week preemie weaning off CPAP
- 4 week female with tachypnea on room air
- 6 week old with post congenital diaphragmatic hernia repair weaning off oxygen

Answer: 4 month old with severe subglottic stenosis who remains intubated despite multiple dilations 

Indications for pediatric tracheotomy primarily include chronic respiratory failure with ventilator dependence, neurologic impairment, congenital airway anomalies, craniofacial anomalies, trauma, and vocal-fold dysfunction.

**Q4 Once a patient is weaned off the ventilator during the day, which device do you recommend to keep on the trach to maintain adequate humidification?**

- Heat Moisture Exchanger (HME) (1)
- Speaking valve (2)
- Plastic enclosed cap (3)
- Nothing is needed (4)

Answer: Heat Moisture Exchanger (HME) 

The air we breathe bypasses the body’s natural way to warm, humidify and filter the air – the nose. With a trach tube, we are no longer utilizing the nose as a humidifier.  

The inside of the nasal cavity and the trachea and down through the bronchi are lined with cells that produces a layer of mucus. This mucus layer traps dust and any particles. Cilia sweep constantly to move the mucus up to the end of the trach tube. This mucus is then coughed up and spit out or swallowed. The airway needs to have good humidification and warmth to keep this layer of mucus thin and allow the cilia to function properly.

 If the air is not humified, it will dry out the mucus in the lungs and the trach tube leading to an occlusion. 

An HME is a small “nose-like” piece of plastic with a paper filter that fits over the trach opening. Each end of the HME is made up of rows and rows of thin filter paper. The HME helps with humidification by trapping some of the warm moist air when your child breaths out through their trach. When your child breathes back in, the air passes through the HME and picks up the moisture from the filter paper. 

**Q5 3-year-old ex-26 preemie who is tracheostomy and ventilator dependent presents to the ER with 3 day history of increased secretions. She started developing rhinorrhea and cough 3 days prior. They are frequently suctioning. Her humidification is working at home. In the ER, her saturations are normal and she does not appear to be in distress.  You obtain a CXR and there is no evidence of pneumonia.  What is the next best step in management?**

- Observation only
- Obtain tracheostomy culture and start empiric antibiotics
- Admit and start IV antibiotics
- Increase airway clearance at home

Answer: Increase airway clearance at home. 

She most likely has a upper respiratory infection and does not require antibiotics for admission as she does not appear ill or require increase ventilator support. 

As there is increase risk for development of lower respiratory tract infection, it would be recommended for this patient to increase her airway clearance regimen.  This is especially for patients with neuromuscular disorders such as hypotonia or cognitive delays leading to inability to manage their secretions. These patients are at risk for development worsening respiratory distress from their viral illnesses due to poor mucous clearance. 

The steps of airway clearance include
1. Bronchodilator such as albuterol to widen and relax the airways. Albuterol has been also shown to improve cilia movement, therefore this will help move the mucus out of the lungs 
2. Hypertonic saline: if secretions are extremely thick, this can be given to loosen up the secretions to make it easier to be expectorated or suctioned 
3. Chest Percussion- this helps loosen the mucus from the airway

Goals of airway clearance is to clear the mucous from the lungs and prevent atelectasis. 

Q5 A 9 year old presents with a 2 year history of chronic cough interspersed with some periods of relief. The cough is harsh and barky. She has been prescribed multiple courses of steroids and antibiotics which have not helped. Mother notes that she does not cough during sleep. On physical exam, she is coughing intermittently. You distract her by talking to her about her favorite movie and notice that the cough is less frequent. Lungs sound clear on exam. 
What is the next step in management?

- spirometry with bronchodilator response
- CXR
- Inhaled daily steroids
- Behavioral therapy
- Reassurance

Answer D: Behavioral therapy 

 There are many causes of chronic cough in childhood. This girl most likely has habit cough and behavioral therapy is the best treatment for it.  The cough usually begins after an upper respiratory infection, but the cough persists long after the other respiratory symptoms resolve. Typically, the cough is harsh, barking, and nonproductive. Characteristically, it disappears during sleep or distraction and is not exacerbated by physical exercise.
 
The diagnosis of habit cough requires a thorough evaluation to rule out other causes of chronic cough. 

 The mean age for this disorder has been identified as 10 years. Eighty-five percent of 120 children at University of Iowa program were between 8 and 14 years of age.

 Various therapeutic approaches have been proposed, including explanation and reassurance, hypnosis, and suggestion therapy. 
 
Habit cough may be associated with psychosocial stressors. The most common are school problems as the frequent loud coughing can be very disruptive in a classroom and may lead to school absenteeism, which can reinforce the cough.

End of Block: Default Question Block
