# Supplementary material for: Development and Implementation of a Pediatric Pulmonary–Focused Active Learning Curriculum
Source: MedEdPORTAL. 2024 Dec 6;20:11470. doi: 10.15766/mep_2374-8265.11470 (PMC11621239; doi:10.15766/mep_2374-8265.11470)
Supplement: Supplementary file 1 — Asthma Module folderTracheostomy Module folderChronic Cough Module folderObstructive Sleep Apnea Module folderPosttest Questions.docxFeedback.docx [file mep_2374-8265.11470-s001.zip › F. Feedback.docx]

**Feedback**

Thank you for taking the time to provide feedback on the modules. Your insights are valuable in helping us improve and enhance the content.

**1. Did you participate in any of the following modules? (Please select all that apply.)**

[ ] OSA (Obstructive Sleep Apnea)

[ ] Tracheostomy

[ ] Chronic Cough

[ ] Asthma

**2. What strengths did you find in the modules you participated in? (Please describe any aspects you particularly liked.)**

**3. Is there any additional information or content you would recommend adding to the modules?**

**4. Was there any part of the modules that you felt could be improved or changed? If so, please specify.**
